# Bromothiolation of Arynes for the Synthesis of 2-Bromobenzenethiol Equivalents

**DOI:** 10.1021/acs.orglett.4c00944

**Published:** 2024-04-30

**Authors:** Shinya Tabata, Suguru Yoshida

**Affiliations:** Department of Biological Science and Technology, Faculty of Advanced Engineering, Tokyo University of Science, 6-3-1 Niijuku, Katsushika-ku, Tokyo 125-8585, Japan

## Abstract

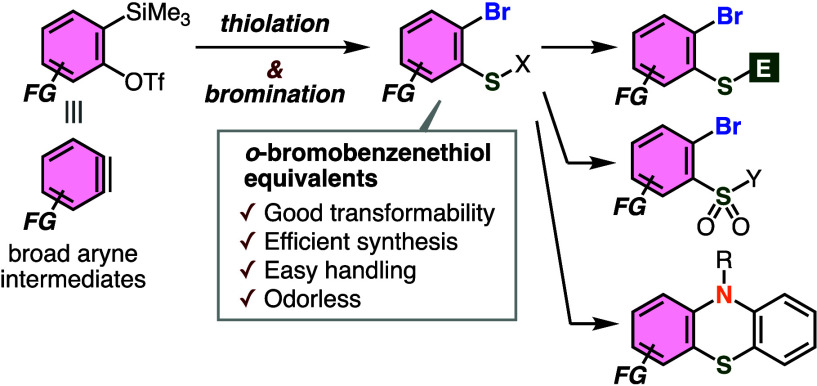

A new method to synthesize *o*-bromobenzenethiol equivalents through aryne intermediates is disclosed. Various *o*-bromobenzenethiol equivalents are prepared by the bromothiolation of aryne intermediates with potassium xanthates. Aryl xanthates serve in the synthesis of diverse organosulfurs involving phenothiazines and thianthrenes by further transformations.

A wide variety of molecules having organosulfur skeletons such as phenothiazine, thianthrene, thienothiophene, and so on play pivotal roles in broad research fields (Figure 1A).^1^ A number of methods to construct these significant organosulfur skeletons from *o*-bromobenzenethiols have been developed by virtue of good reactivities of thiol and bromide moieties.^2^ However, it is not easy to prepare diverse *o*-bromobenzenethiols from the corresponding anilines by conventional methods due to the regioselectivity in *ortho*-bromination of the amino group and poor functional group tolerance in the following diazotization–thiolation through highly reactive diazonium intermediates (Figure 1B).^3^ In addition, high oxidizability under air as well as the bad smell of *o*-bromobenzenethiols makes the synthesis of highly functionalized derivatives difficult. Herein, we disclose a new method to prepare *o*-bromobenzenethiol equivalents by bromothiolation of aryne intermediates (Figure 1C).

**Figure 1:**
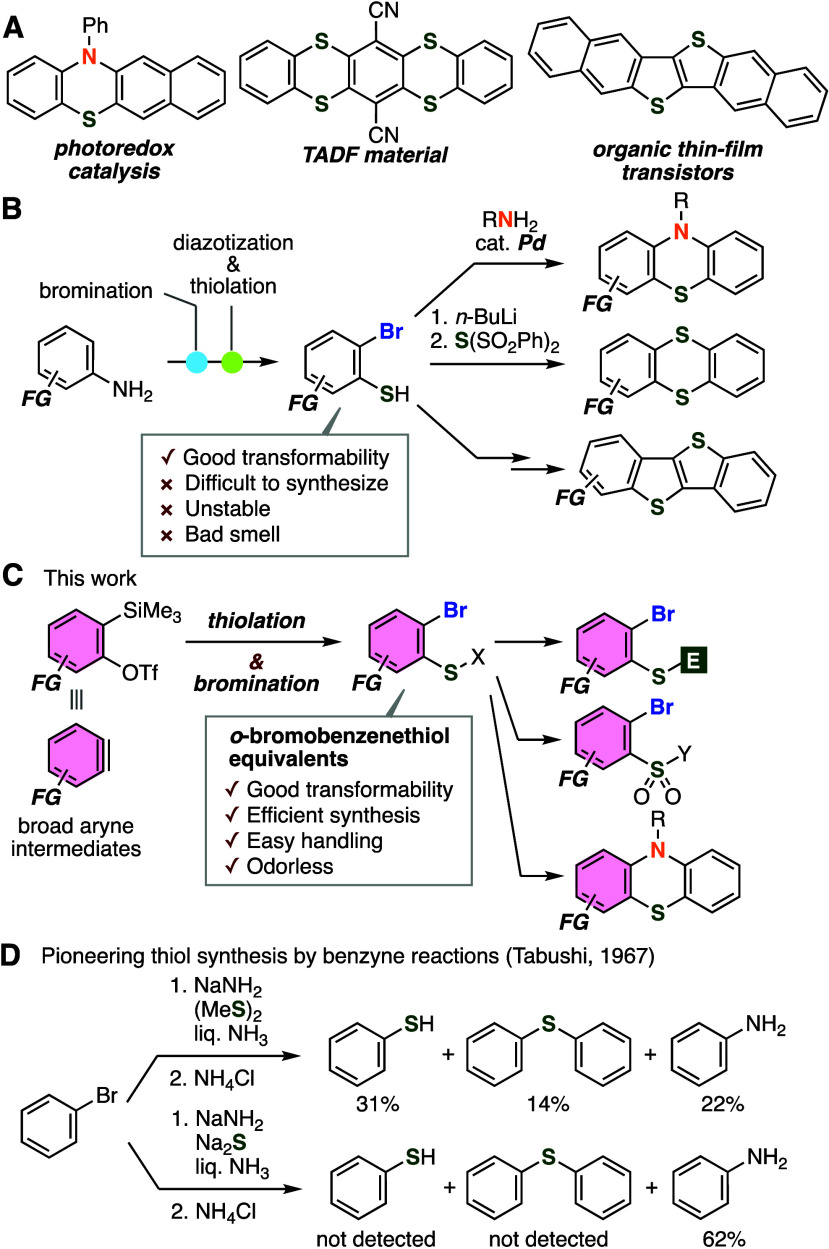
(A) Important organosulfur skeletons. (B) Conventional synthesis using *o*-bromobenzenethiols. (C) Overview of this work. (D) Pioneering study for thiol synthesis via benzyne.

Difunctionalizations of aryne intermediates have gained attention as fascinating methods due to the rapidly growing synthetic aryne chemistry.^4^ Recently, regioselective transformations of a wide range of aryne intermediates have enabled us to synthesize diverse highly functionalized aromatic compounds. Despite the emerging achievements in aryne chemistry, *o*-bromobenzenethiol synthesis by bromothiolation of aryne intermediates is challenging since diverse organosulfurs show high reactivity toward arynes.^5,6^ Indeed, a pioneering study for thiol synthesis by thiolation of benzyne using dimethyl disulfide or sodium sulfide did not accomplish efficient preparation of benzenethiol, in which further arylation of benzenethiol with benzyne easily took place (Figure 1D).^7^ To address this issue, we conceived an idea that stable *o*-bromobenzenethiol equivalents can be synthesized by bromothiolation of aryne intermediates with an appropriate hydrogen sulfide equivalent and an electrophilic brominating reagent without further arylation of products by virtue of the controlled reactivity at the sulfur atom (Figure 1C). Further C–S bond formations of *o*-bromobenzenethiol equivalents through the in situ preparation of thiols will allow us to synthesize diverse organosulfurs. In addition, the good stability of *o*-bromobenzenethiol equivalents would serve in the organosulfur synthesis with easy handling in an odorless manner.

First, sulfur surrogates were screened for the preparation of benzenethiol equivalents by the reaction of an aryne generated from *o*-silylaryl triflate **1a** (Figure 2A). As a result, we found that the treatment of potassium xanthate **2a** with *o*-silylaryl triflate **1a** in the presence of potassium fluoride and 18-crown-6 in acetonitrile afforded aryl xanthate **3a** quantitatively. In this reaction, thiolation followed by protonation efficiently took place to furnish aryl xanthate **3a**, which can serve as a thiol equivalent by hydrolysis under basic conditions. When the reaction was performed on a 1 mmol scale, we also achieved the synthesis of thiolated product **3a** in high yield, clearly showing good practicality. Yields of aryl xanthates **4–6** by the hydrothiolation of 4,5-dimethoxybenzyne were significantly decreased when using other sulfur surrogates **2b**–**2d**. Then, after extensive screening of brominating reagents instead of protonation,^8^ we found that efficient bromothiolation of the aryne intermediate proceeded smoothly to provide *o*-bromobenzenethiol equivalent **8a** in good yield when *o*-silylaryl triflate **1a** was treated with potassium fluoride and 18-crown-6 in the presence of potassium xanthate **2a** and pentafluorophenyl bromide (**7a**)^8a^ in 1,2-dimethyoxyethane (Figure 2B). In contrast, the efficiency of bromination was remarkably diminished in the case of tetrabromomethane (**7b**) or *N*-bromosuccinimide (**7c**) instead of pentafluorophenyl bromide (**7a**). The bromothiolation also occurred to afford **8a** when using bromoalkyne **7d** or **7e**.^8b^ In addition, bromination did not proceed when 4-fluorophenyl bromide (**7f**) was used as a brominating reagent, clearly indicating that fluoro groups significantly enhanced the reactivity of **7a** in the electrophilic bromination. Furthermore, we succeeded in the iodothiolation furnishing *o*-iodobenzenethiol equivalent **10** in high yield when iodoalkyne **9** was used as an iodine source (Figure 2C).

**Figure 2:**
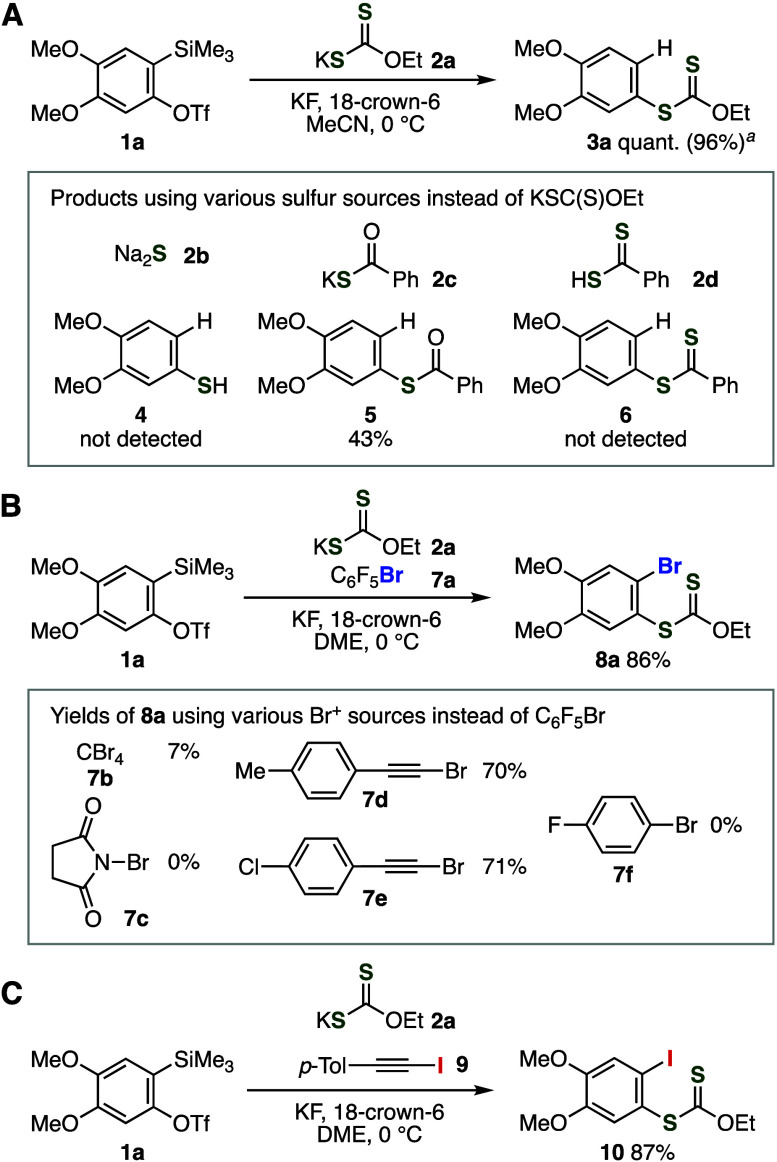
(A) Syntheses of aryl xanthate **3a**. (B) Syntheses of aryl xanthate **8a**. (C) Syntheses of aryl xanthate **10**. ^*a*^The reaction was conducted on a 1 mmol scale.

A broad range of aryl xanthates **3** and **8** were synthesized by aryne reactions of potassium xanthate **2a** using various *o*-silylaryl triflates **1** (Figure 3). First, hydrothiolation of aryne intermediates realized the efficient synthesis of highly functionalized aryl xanthates by method A (Figure 3A and 3B). Symmetric arynes smoothly reacted with potassium xanthate **2a** to afford aromatic xanthates **3b**–**3d** in excellent yields without damaging benzodioxole and fluoro moieties. We realized the preparation of adducts **3e**–**3j** through 3-substituted arynes in good yields without regioisomers. In the case of hydrothiolation of 3-methoxybenzyne generated from 3-methoxy-2-(trimethylsilyl)phenyl triflate (**1e**), *m*-methoxyphenyl xanthate **3e** was obtained via regioselective C–S bond formation. Of note, we succeeded in the selective synthesis of aryl xanthate **3f** in good yield without triazole formation at the azido group, which demonstrates higher reactivity of xanthate ion toward aryne intermediate than that of the azido group.^9,10^ Regioselective hydrothiolation of 3-bromo- and 3-chlorobenzyne also took place smoothly to provide aryl xanthates **3g** and **3h** in high yields.^11^ Furthermore, aryl xanthates **3i** and **3j** having morpholino and 4-methoxyphenylthio groups were successfully synthesized by the regioselective hydrothiolation of the corresponding aryne intermediates without further arylation of a nitrogen or sulfur atom. Since the synthesis of *o*-silylaryl triflates with morpholino and 4-methoxyphenylthio groups was achieved by amino- and thiosilylation of 3-(triflyloxy)benzyne generated from 1,3-bis(triflyloxy)-2-iodobenzene under the mild conditions according to our recently developed methods, a wide variety of 3-amino- and 3-sulfanyl-substituted aryl xanthates can be prepared in a modular synthetic manner.^12^ Naphthyl xanthate **3k** was efficiently synthesized in a good yield through 2,3-naphthalyne. When 2-(trimethylsilyl)-3-pyridyl triflate or *N*-methyl-7-(trimethylsilyl)-6-indolyl triflate was used as a 2,3-pyridyne or 6,7-indolyne precursor, regioselective hydrothiolation proceeded efficiently to furnish 3-pyridyl or 6-indolyl xanthate **3l** or **3m**, respectively.^13,14^

**Figure 3:**
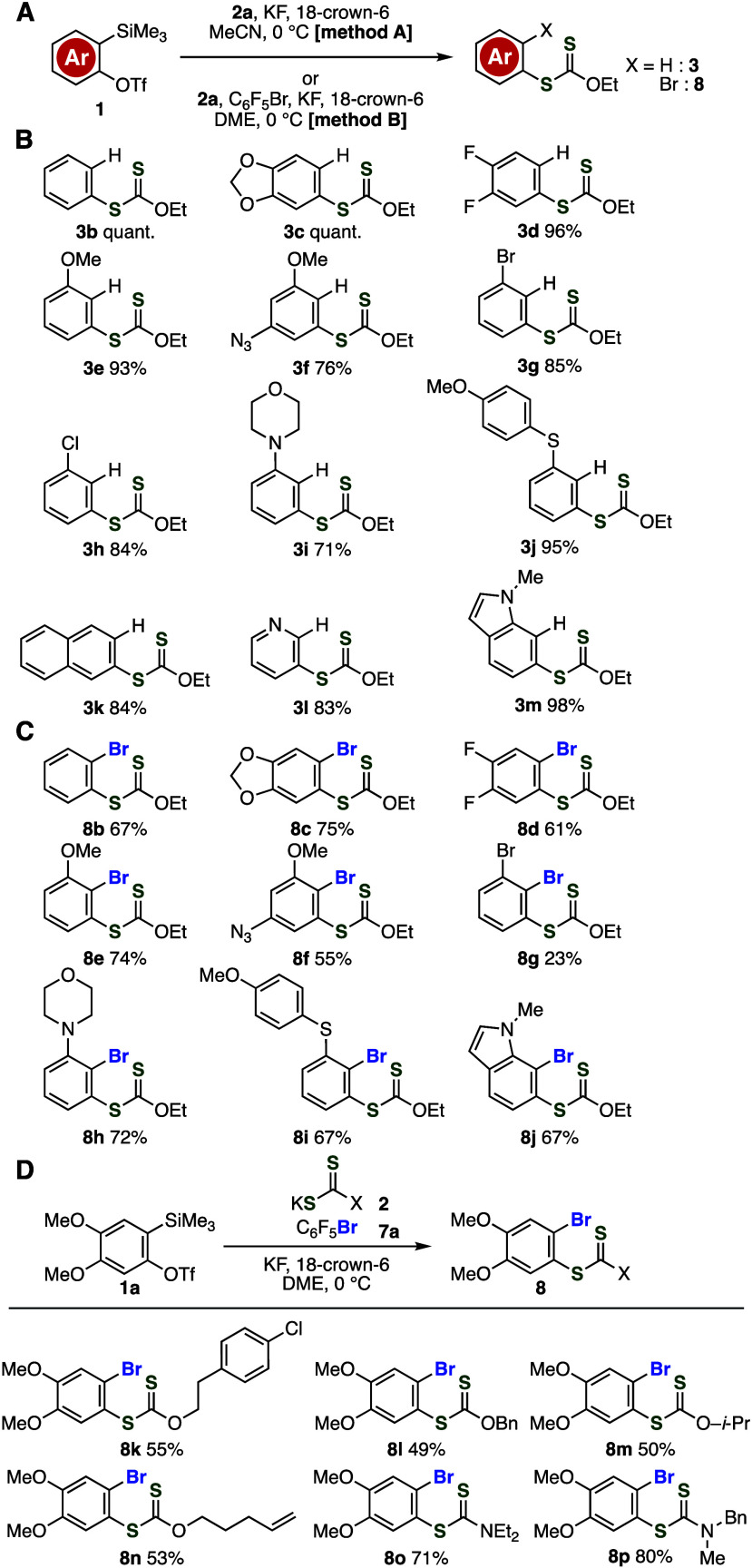
(A) General scheme. (B) Syntheses of various aryl xanthates **3**. (C) Syntheses of various aryl xanthates **8**. (D) Syntheses of various aryl xanthates **8** using various potassium xanthates **2**. See the Supporting Information for details.

We succeeded in the synthesis of *o*-bromobenzenethiol derivatives **8** bearing a wide range of functional groups by bromothiolation of aryne intermediates by method B (Figure 3A and 3C).^15^ Difunctionalization of symmetric arynes resulted in the efficient preparation of *o*-bromoaryl xanthates **8b**–**8d**. Regioselective synthesis of 1,2,3-substituted benzene **8e** was accomplished, in which C–S bond formation at the 1-position and C–Br bond formation at the 2-position of 3-methoxybenzyne proceeded. It is worth noting that preparation of tetrasubstituted benzene **8f** was realized by the regioselective bromothiolation leaving bromo, xanthate, methoxy, and azide moieties untouched. In the case of 3-bromobenzyne, 2,3-dibromophenyl xanthate **8g** was obtained in a low yield. We achieved the efficient synthesis of highly functionalized aryl xanthates **8h** and **8i** by bromothiolation without the formation of regioisomers. Moreover, regioselective bromothiolation of 6,7-indolyne took place smoothly to provide heteroaromatic xanthate **8j** in a good yield. These results clearly show the good functional group tolerance in bromothiolation of aryne intermediates, allowing us to synthesize diverse aryl xanthates from highly functionalized arynes involving hetarynes by virtue of recent remarkable achievements in the synthetic aryne chemistry.

Various potassium xanthates or dithiocarbamates successfully participated in the bromothiolation of aryne intermediates (Figure 3D). For instance, we accomplished the synthesis of aryl xanthates **8k**–**8n** using potassium xanthates bearing a 2-(4-chlorophenyl)ethyl, benzyl, isopropyl, or 4-pentenyl group in moderate yields. Dithiocarbamates **8o** and **8p** possessing ethyl, methyl, and benzyl groups were efficiently synthesized by bromothiolation of 4,5-dimethoxybenzyne using the corresponding potassium xanthates.

To obtain insight into the reaction mechanism, we performed several control experiments for the aryne reactions using potassium xanthate (Figures 4A–4D). We examined the reactivity of aryl xanthate **3a** toward aryne intermediates from *o*-silylaryl triflate **1a** in the presence of activators resulting in the partial consumption of aryl xanthate (30%), suggesting higher reactivity of xanthate anion than that of products led to good efficiency of the aryl xanthate synthesis (Figure 4A). When we used sodium xanthate **2a′** instead of potassium xanthate **2a**, the yield of **3a** was slightly decreased (Figure 4B). This result indicates that counter cations are related to the nucleophilicity of xanthate anions in the presence of 18-crown-6 for the complexation of potassium cation. Then, we succeeded in the deuteration experiment for the hydrothiolation of aryne intermediates using acetonitrile-*d*_3_, clearly showing that acetonitrile used as a solvent facilitated the protonation of aryl anions generated by the nucleophilic addition of xanthate anions to aryne intermediates (Figure 4C). Control experiments for reactions of **1a** with potassium fluoride after pretreatment of pentafluorophenyl bromide (**7a**) or NBS (**7c**) with potassium xanthate **2a** in the presence of 18-crown-6 suggested the reaction mechanism (Figure 4D). Aryl xanthate **8a** was synthesized in good yield when pentafluorophenyl bromide (**7a**) was used (Figure 4D, upper). This result shows the good stability of reagents used, in which nucleophilic reagent **2a** did not react with electrophilic bromide source **7a**.^16^ In the case of using NBS (**7c**) instead of **7a**, products derived from the reaction of potassium xanthate **2a** with NBS were not obtained, where we found that the formation of *o*-bromoaryl triflate **11** took place by the reaction between aryne precursor **1a** with NBS (Figure 4D, lower). Thus, a key to the success in the bromothiolation of arynes lies in the mild reactivity of electrophilic bromide source **7a**, which does not react with both *o*-silylaryl triflate **1a** and potassium xanthate **2a**.

**Figure 4:**
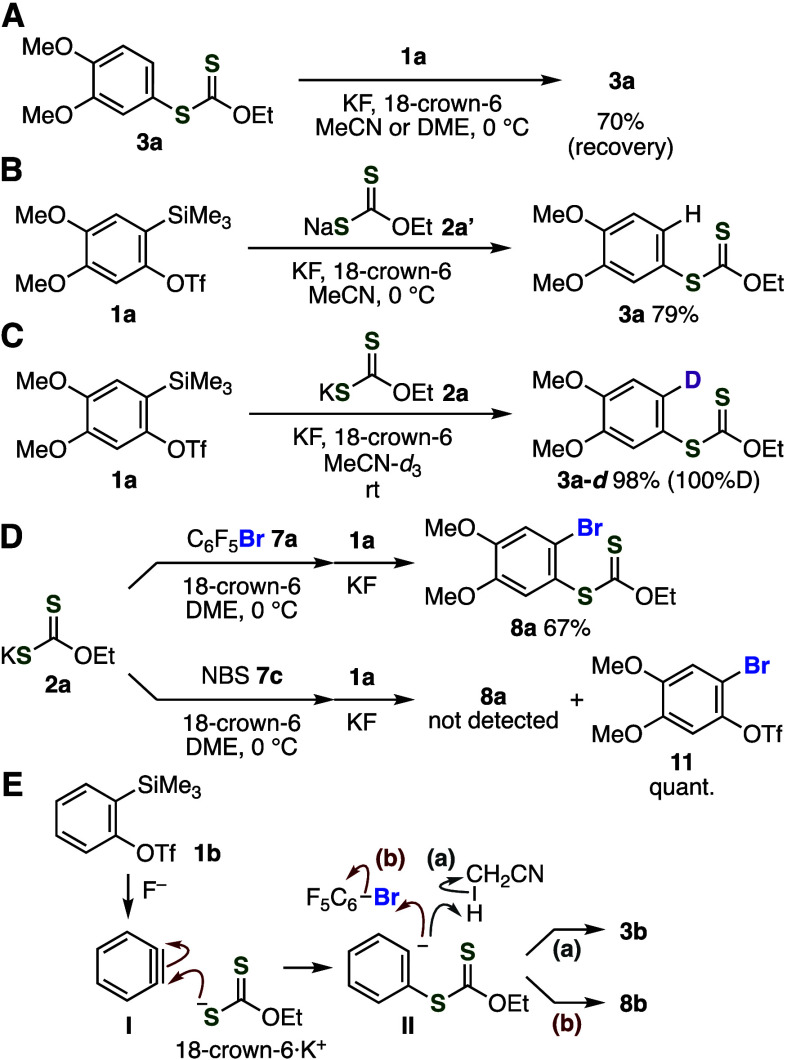
(A) Stability of **3a** under the optimized conditions. (B) Synthesis of **3a** using sodium xanthate **2a′**. (C) Synthesis of **3a**-*d* using MeCN-*d*_3_. (D) Reactions of **1a** after pretreatment of potassium xanthate **2a** with bromide **7a** or **7c**. (E) Plausible reaction mechanisms.

On the basis of control experiments, a plausible reaction mechanism is shown in Figure 4E. First, nucleophilic attack of xanthate anion to benzyne (**I**) generated from **1b** led to the formation of aryl anion **II**. Then, the protonation of aryl anion **II** from acetonitrile affords aryl xanthate **3b** as a hydrothiolation product (path a). In the bromothiolation, pentafluorophenyl bromide (**7a**) serves as an electrophilic bromide source, leading to *o*-bromobenzenethiol equivalent **8b**.

A wide variety of organosulfur compounds were easily synthesized from aryl xanthate **3a** (Figure 5A). For example, hydrolysis of aryl xanthate **3a** smoothly took place under the basic conditions and following oxidation of the resulting thiol under air to afford disulfide **12** in an excellent yield, which also shows the air-sensitive nature of electron-rich thiols.^17^ Then, we achieved the synthesis of sulfide **13a** or **13b** by hydrolysis under basic conditions followed by *S*-alkylation with iodomethane or *S*-arylation with *o*-fluoronitrobenzene in a one-pot manner. Oxidation of aryl xanthate **3a** with *N*-chlorosuccinimide (NCS) under acidic conditions led to the formation of sulfonyl chloride **14a** in high yield.^18^ Furthermore, we succeeded in the preparation of sulfonyl fluoride **14b** from aryl xanthate **3a** with potassium hydrogenfluoride and Selectfluor.^17^ Since benzenethiol equivalent synthesis through aryne intermediates and subsequent oxidation enable us to prepare diverse aryl sulfonyl fluorides which are useful for the sulfur exchange (SuFEx) reactions, aryl xanthate formation developed in this study will serve in broad research fields due to the significant importance of the click chemistry.^19^

**Figure 5:**
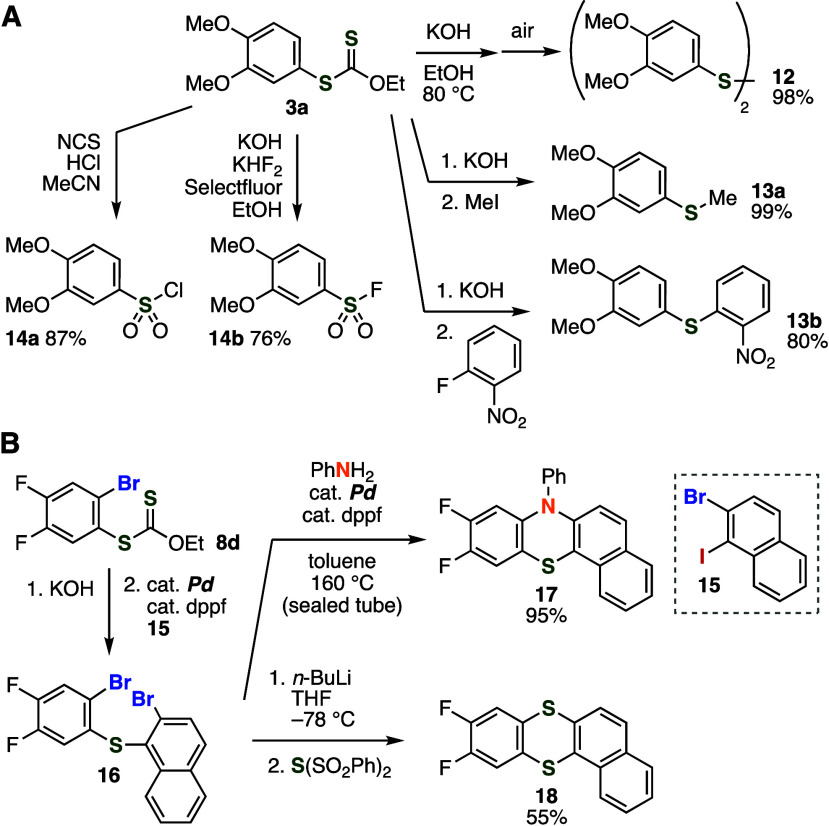
(A) Transformations of **3a**. (B) Construction of organosulfur skeletons using **8d**. See the Supporting Information for the details.

We showcased the *o*-bromobenzenethiol equivalent synthesis by further transformations for the construction of fused organosulfur skeletons (Figure 5B). First, hydrolysis of aryl xanthate **8d** and subsequent palladium-catalyzed *S*-arylation with aryl iodide **15** proceeded efficiently to furnish diaryl sulfide **16**. Second, ring-closure transformations of diaryl sulfide **16** allowed us to form tetracyclic scaffolds through the cleavage of two C–Br bonds.^2e^ For instance, treatment of diaryl sulfide **16** with aniline under palladium catalysis provided phenothiazine **17** leaving fluoro- and phenyl groups intact. In addition, we accomplished the synthesis of thianthrene **18** via double lithiation of dibromide **16** by *n*-butyllithium and double thiolation using bis(phenylsulfonyl) sulfide without damaging fluoro groups.^20^ Owing to the good accessibility of aryne precursors and diverse transformations using *o*-bromobenzenethiols, a wide variety of organosulfur compounds having highly fused organosulfur skeletons will be prepared through the bromothiolation of aryne intermediates.

In conclusion, we have developed a new method to synthesize *o*-bromobenzenethiol equivalents via aryne intermediates. Potassium xanthates showed good reactivity in aryne reactions. The resulting aryl xanthates served in the preparation of diverse highly functionalized organosulfurs involving phenothiazines and thianthrenes by simple protocols in an odorless fashion. Further studies such as the expansion of scope and applications to synthesize bioactive organosulfur compounds are underway in our laboratory.

## Data Availability

The data underlying this study are available in the published article and its Supporting Information.

## Supporting Information Available

Experimental procedures, characterization for new compounds including NMR spectra (PDF)

## References

[ref1] aWang C.; Dong H.; Hu W.; Liu Y.; Zhu D. Semiconducting π-Conjugated Systems in Field-Effect Transistors: A Material Odyssey of Organic Electronics. Chem. Rev. 2012, 112, 2208 10.1021/cr100380z. 22111507

[ref2] aDahl T.; Tornøe C. W.; Bang-Andersen B.; Nielsen P.; Jørgensen M. Palladium-Catalyzed Three-Component Approach to Promazine with Formation of One Carbon-Sulfur and Two Carbon-Nitrogen Bonds. Angew. Chem., Int. Ed. 2008, 47, 1726 10.1002/anie.200705209. 18213669

[ref3] aCampaigne E.; Osborn S. Improved Procedure for the Preparation of Aromatic Thiols. J. Org. Chem. 1957, 22, 561 10.1021/jo01356a602.

[ref4] aTadross P. M.; Stoltz B. M. A Comprehensive History of Arynes in Natural Product Total Synthesis. Chem. Rev. 2012, 112, 3550 10.1021/cr200478h. 22443517

[ref5] aMatsuzawa T.; Yoshida S.; Hosoya T. Recent advances in reactions between arynes and organosulfur compounds. Tetrahedron Lett. 2018, 59, 4197 10.1016/j.tetlet.2018.10.031.

[ref6] aLin W.; Sapountzis I.; Knochel P. Preparation of Functionalized Aryl Magnesium Reagents by the Addition of Magnesium ArylThiolates and Amides to Arynes. Angew. Chem., Int. Ed. 2005, 44, 4258 10.1002/anie.200500443. 15940729

[ref7] Tabushi I.; Okazaki K.; Oda R. Reaction of benzyne with disulfides. Tetrahedron Lett. 1967, 8, 3591 10.1016/S0040-4039(01)89802-6.

[ref8] aYoshida H.; Asatsu Y.; Mimura Y.; Ito Y.; Ohshita J.; Takaki K. Three-Component Coupling of Arynes and Organic Bromides. Angew. Chem., Int. Ed. 2011, 50, 9676 10.1002/anie.201104858. 21887828

[ref9] Yoshida S.; Shimomori K.; Nonaka T.; Hosoya T. Facile Synthesis of Diverse Multisubstituted *ortho*-Silylaryl Triflates via C-H Borylation. Chem. Lett. 2015, 44, 1324 10.1246/cl.150535.

[ref10] Shi F.; Waldo J. P.; Chen Y.; Larock R. C. Benzyne Click Chemistry: Synthesis of Benzotriazoles from Benzynes and Azides. Org. Lett. 2008, 10, 2409 10.1021/ol800675u. 18476707 PMC3750119

[ref11] Medina J. M.; Mackey J. L.; Garg N. K.; Houk K. N. The Role of Aryne Distortions, Steric Effects, and Charges in Regioselectivities of Aryne Reactions. J. Am. Chem. Soc. 2014, 136, 15798 10.1021/ja5099935. 25303232 PMC4221504

[ref12] aYoshida S.; Nakamura Y.; Uchida K.; Hazama Y.; Hosoya T. Aryne Relay Chemistry en Route to Aminoarenes: Synthesis of 3-Aminoaryne Precursors via Regioselective Silylamination of 3-(Triflyloxy)arynes. Org. Lett. 2016, 18, 6212 10.1021/acs.orglett.6b03304. 27934384

[ref13] Fang Y.; Larock R. C. Nucleophilic addition to 2,3-pyridyne and synthesis of benzonaphthyridinones. Tetrahedron Lett. 2012, 68, 2819 10.1016/j.tet.2012.02.002. PMC3600655 23519554

[ref14] Im G-Y. J.; Bronner S. M.; Goetz A. E.; Paton R. S.; Cheong P. H. Y.; Houk K. N.; Garg N. K. Indolyne Experimental and Computational Studies: Synthetic Applications and Origins of Selectivities of Nucleophilic Additions. J. Am. Chem. Soc. 2010, 132, 17933 10.1021/ja1086485. 21114321 PMC3075889

[ref15] In the case of bromothiolation of 2,3-naphthalyne, a small amount of sideproduct through the hydrothiolation was inseparable with the desired product.

[ref16] aSilva-Cuevas C.; Perez-Arrieta C.; Polindara-García L. A.; Lujan-Montelongo J. A. Sulfonyl halide synthesis by thiol oxyhalogenation using NBS/NCS - *i*PrOH. Tetrahedron Lett. 2017, 58, 2244 10.1016/j.tetlet.2017.04.087.

[ref17] Wu S.; Wong T. H.-F.; Righi P.; Melchiorre P. Photochemical Organocatalytic Synthesis of Thioethers from Aryl Chlorides and Alcohols. J. Am. Chem. Soc. 2024, 146, 2907 10.1021/jacs.3c13900. 38265336

[ref18] Bugaenko D. I.; Volkov A. A.; Andreychev V. V.; Karchava A. V. Reaction of Diaryliodonium Salts with Potassium Alkyl Xanthates as an Entry Point to Accessing Organosulfur Compounds. Org. Lett. 2023, 25, 272 10.1021/acs.orglett.2c04143. 36594721

[ref19] Dong J.; Krasnova L.; Finn M. G.; Sharpless K. B. Sulfur(VI) Fluoride Exchange (SuFEx): Another Good Reaction for Click Chemistry. Angew. Chem., Int. Ed. 2014, 53, 9430 10.1002/anie.201309399. 25112519

[ref20] Dötze M.; Klar G. Organometalloidal Compounds with o-Phenylenesubstituents, Part XXVII.1 Synthesis, Characterization and Structure Determination of 2,3,7,8-Tetrakis(methylthio)- and 2,3;7,8-Bis(Ethylenedithio)Thianthrene. Phosphorus, Sulfur, Silicon Relat. Elem. 1993, 84, 95 10.1080/10426509308034319.

