# Supplementary material for: Bromothiolation of Arynes for the Synthesis of 2-Bromobenzenethiol Equivalents
Source: Org Lett. 2024 Apr 30;26(18):3816–21. doi: 10.1021/acs.orglett.4c00944 (PMC11091890; doi:10.1021/acs.orglett.4c00944)

Supporting Information

Bromothiolation of Arynes for the Synthesis of 2-Bromobenzenethiol Equivalents

Shinya Tabata and Suguru Yoshida*

Department of Biological Science and Technology, Faculty of Advanced Engineering, Tokyo University of Science, 6-3-1 Niijuku, Katsushika-ku Tokyo 125-8585
E-mail: s-yoshida@rs.tus.ac.jp

Contents

General Information **S2**
Structures of Aryne Precursors 1 and Sulfur Surrogates 2 **S3**
Experimental Procedures **S4**
Characterization Data of New Compounds **S9**
References for Supporting Information **S17**
^{1}H and ^{13}C NMR Spectra of New Compounds **S18**
^{1}H NMR Spectra of Reported Compounds **S51**

General Information

All reactions were performed with dry glassware under atmosphere of argon, unless otherwise noted. Analytical thin-layer chromatography (TLC) was performed on precoated (0.25 mm) silica-gel plates (Merck Chemicals, Silica Gel 60 F254, Cat. No. 1.05715). Column chromatography was conducted using silica-gel (Kanto Chemical Co., Inc., Silica Gel 60N, spherical neutral, particle size 40–50 µm, Cat. No. 37562-85 or particle size 63–210 µm, Cat. No. 37565-85). Preparative TLC (PTLC) was performed on silica gel (Wako Pure Chemical Industries Ltd., Wakogel B-5F, Cat. No. 230-00043). Melting points (Mp) were measured on an OptiMelt MPA100 (Stanford Research Systems), and are uncorrected. ^{1}H NMR spectra were obtained with a Bruker AVANCE 400 spectrometer at 400 MHz. ^{13}C NMR spectra were obtained with a Bruker AVANCE 400 spectrometer at 101 MHz. ^{19}F NMR spectra were obtained with a Bruker AVANCE 400 spectrometer at 376 MHz. All NMR measurements were carried out at 25 ºC. $CDCl_3$, acetone-d_6, or DMSO-d_6 was used as a solvent for obtaining NMR spectra. Chemical shifts (δ) are given in parts per million (ppm) downfield from the solvent peak (δ 7.26 for ^{1}H NMR in $CDCl_3$, δ 77.0 for ^{13}C NMR in $CDCl_3$; δ 2.09 for ^{1}H NMR in acetone-d_6, δ 30.6 for ^{13}C NMR in acetone-d_6; δ 2.54 for ^{1}H NMR in DMSO-d_6, δ 40.5 for ^{13}C NMR in DMSO-d_6) as an internal reference with coupling constants (*J*) in hertz (Hz). The abbreviations s, d, t, q, and m signify singlet, doublet, triplet, quartet, and multiplet, respectively. IR spectra were measured on a Shimadzu IRSpirit spectrometer with the absorption band given in cm^{-1}. High-resolution mass spectra (HRMS) were measured on a JEOL JMS-T100CS "AccuTOF CS" mass spectrometer under positive electrospray ionization (ESI^+) conditions or negative electrospray ionization (ESI^-) conditions, or JMS-700 (JEOL, Tokyo, Japan) mass spectrometer under electron impact ionization (EI) conditions.

Unless otherwise noted, materials obtained from commercial suppliers were used without further purification. 4,5-Dimethoxy-2-(trimethylsilyl)phenyl trifluoromethanesulfonate (**1a**),[S1] 6-(trimethylsilyl)benzo[*d*][1,3]dioxol-5-yl trifluoromethanesulfonate (**1c**),[S2] 4,5-difluoro-2-(trimethylsilyl)phenyl trifluoromethanesulfonate (**1d**),[S3] 3-methoxy-2-(trimethylsilyl)phenyl trifluoromethanesulfonate (**1e**),[S4] 5-azido-3-methoxy-2-(trimethylsilyl)phenyl trifluoromethanesulfonate (**1f**),[S5] 2-bromo-3-(trimethylsilyl)phenyl trifluoromethanesulfonate (**1g**),[S6] 3-morpholino-2-(trimethylsilyl)phenyl trifluoromethanesulfonate (**1i**),[S7] 3-((4-methoxyphenyl)thio)-2-(trimethylsilyl)phenyl trifluoromethanesulfonate (**1j**),[S8] 3-(trimethylsilyl)pyridin-2-yl trifluoromethanesulfonate (**1l**),[S9] 1-methyl-7-(trimethylsilyl)-1*H*-indol-6-yl trifluoromethanesulfonate (**1m**),[S10] potassium *O*-benzyl carbonodithioate (**2f**),[S11] potassium *O*-isopropyl carbonodithioate (**2g**),[S11] potassium diethylcarbamodithioate (**2i**),[S11] potassium (benzyl)methylcarbamodithioate (**2j**),[S11] 1-(bromoethynyl)-4-methylbenzene (**7d**),[S12] 1-(bromoethynyl)-4-chlorobenzene (**7e**),[S13] 1-(iodoethynyl)-4-methylbenzene (**9**),[S14] and 2-bromo-1-iodonaphthalene (**15**)[S15] were prepared according to the reported methods.

Structures of Aryne Precursors 1 and Sulfur Surrogates 2

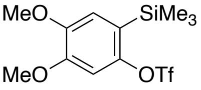

1a

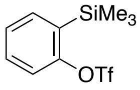

1b

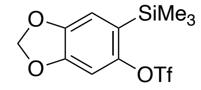

1c

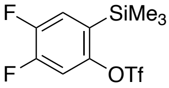

1d

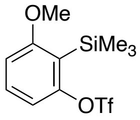

1e

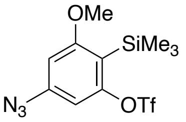

1f

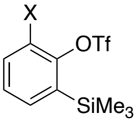

X = Br : **1g**
Cl : **1h**

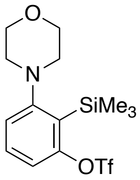

1i

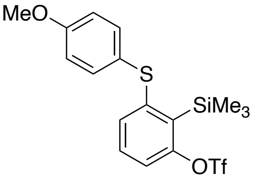

1j

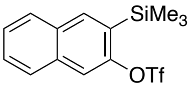

1k

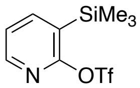

1l

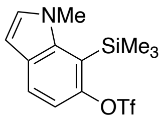

1m

Na_2S **2b**

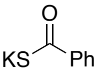

2c

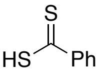

2d

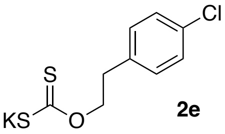

2e

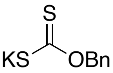

2f

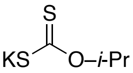

2g

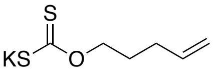

2h

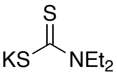

2i

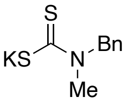

2j

Experimental Procedures

A typical procedure for the thiolation of arynes using potassium xanthate

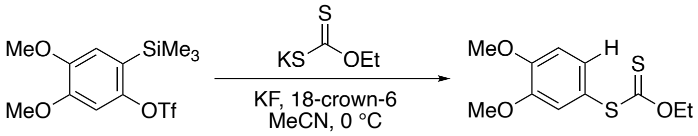

In a 5 mL screw-top V-vial® with a solid-top cap (Sigma–Aldrich, Cat. No. Z115118), to a mixture of 4,5-dimethoxy-2-trimethylsilylphenyl triflate (**1a**) (135 mg, 0.375 mmol, 1.5 equiv) and potassium *O*-ethyl dithiocarbonate (**2a**) (39.7 mg, 0.248 mmol) dissolved in acetonitrile (3.3 mL) were added 18-crown-6-ether (270 mg, 1.02 mmol, 4.1 equiv) and potassium fluoride (41.5 mg, 0.714 mmol, 2.9 equiv) at room temperature. After stirring for 24 h at 0 ºC, the mixture was warmed to room temperature. The mixture was filtered with a short pad of celite. The filtrate was concentrated under reduced pressure. The residue was purified by preparative TLC (*n*-hexane/EtOAc =4/1) to give *S*-(3,4-dimethoxyphenyl) *O*-ethyl carbonodithioate (**3a**) (64.1 mg, 0.248 mmol, quantitative) as a colorless oil.

Similarly, *S*-aryl *O*-ethyl carbonodithioates **3b**–**m** were prepared from the corresponding *o*-silylaryl triflates.

Thiolation of arynes using potassium xanthate in 1 mmol scale

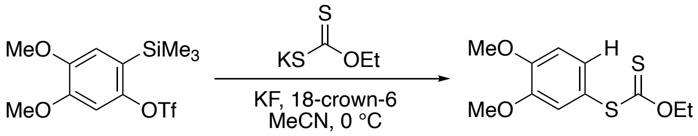

To a mixture of 4,5-dimethoxy-2-trimethylsilylphenyl triflate (**1a**) (538 mg, 1.50 mmol, 1.5 equiv) and potassium *O*-ethyl dithiocarbonate (**2a**) (161 mg, 1.00 mmol) dissolved in acetonitrile (13.3 mL) were added 18-crown-6-ether (1.06 g, 4.01 mmol, 4.0 equiv) and potassium fluoride (174 mg, 3.00 mmol, 3.0 equiv) at room temperature. After stirring for 24 h at 0 ºC, the mixture was warmed to room temperature. The mixture was filtered with a short pad of celite. The filtrate was concentrated under reduced pressure. The residue was purified by column chromatography (silica-gel 30 g, *n*-hexane/EtOAc =4/1) to give *S*-(3,4-dimethoxyphenyl) *O*-ethyl carbonodithioate (**3a**) (250 mg, 0.962 mmol, 96%) as a colorless oil.

A typical procedure for the bromothiolation of arynes using potassium xanthate and bromopentafluorobenzene

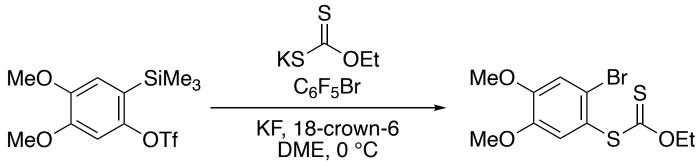

In a 5 mL screw-top V-vial® with a solid-top cap (Sigma–Aldrich, Cat. No. Z115118), to a mixture of 4,5-dimethoxy-2-trimethylsilylphenyl triflate (**1a**) (26.7 mg, 74.5 µmol, 1.5 equiv) and potassium *O*-ethyl dithiocarbonate (**2a**) (8.0 mg, 50 µmol) dissolved in 1,2-dimethoxyethane (0.67 mL) were added bromopentafluorobenzene (31.6 µL, 0.250 mmol, 5.0 equiv), 18-crown-6-ether (52.8 mg, 0.200 mmol, 4.0 equiv), and potassium fluoride (8.7 mg, 0.15 mmol, 3.0 equiv) at room temperature. After stirring for 24 h at 0 °C, the mixture was warmed to room temperature. The mixture was filtered with a short pad of celite. The filtrate was concentrated under reduced pressure. The residue was purified by preparative TLC (*n*-hexane/EtOAc = 4/1) to give *S*-(2-bromo-4,5-dimethoxyphenyl) *O*-ethyl carbonodithioate (**8a**) (14.6 mg, 43.2 µmol, 86%) as a colorless solid.

Similarly, *S*-aryl *O*-alkyl carbonodithioates **8b**–**n** and *S*-aryl *N*-alkyl carbonodithioates **8o-p** were prepared the corresponding *o*-silylaryl triflates.

*Preparation of potassium O-(pent-4-en-1-yl) carbonodithioate (**2h**)*

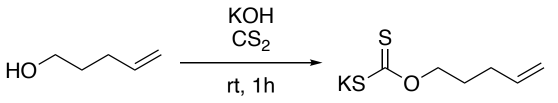

To a round-bottom flask were added KOH (55.8 mg, 0.994 mmol, 1.0 equiv) and 4-penten-1-ol (152 µL, 1.50 mmol, 1.5 equiv) at room temperature. After stirring for 30 min at the same temperature, to the mixture was added dropwise CS_2 (600 µL, 9.93 mmol, 10 equiv) at 0 °C. After stirring for 1 h at room temperature, the mixture was concentrated under reduced pressure. The residue was washed with Et_2O (15 mL) to give potassium *O*-(pent-4-en-1-yl) carbonodithioate (**2h**) (162.5 mg, 0.811 mmol, 82%) as a colorless solid, which was used without further purification.

Similarly, potassium xanthates **2e**–**g**, **2i**, and **2j** were prepared from the corresponding alcohols or amines.

Synthesis of bis(3,4-dimethoxyphenyl) disulfide (12)

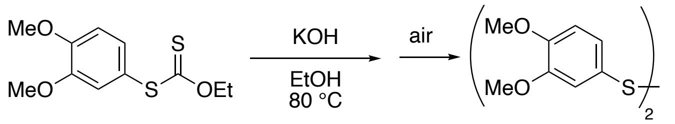

In a 5 mL screw-top V-vial® with a solid-top cap (Sigma–Aldrich, Cat. No. Z115118), to a mixture of *S*-(3,4-dimethoxyphenyl) *O*-ethyl carbonodithioate (**3a**) (35.5 mg, 0.134 mmol) and ethanol (0.400 mL) was added KOH (32.3 mg, 0.576 mmol, 4.3 equiv) at room temperature. The mixture was stirred at 80 °C (oil bath, bath temp. 80 °C) for 12 h. The resulting mixture was cooled to room temperature and acidified with 1 M aqueous HCl solution (4 mL). The mixture was extracted with EtOAc (5 mL × 3). The combined organic extract was washed with brine (10 mL). The extract was then dried with Na_2SO_4. After filtration, the filtrate was concentrated under reduced pressure to afford a mixture of 4,5-dimethoxybenzenethiol and bis(3,4-dimethoxyphenyl) disulfane (**12**). After the mixture was placed under air for 19 h, bis(3,4-dimethoxyphenyl) disulfane (**12**) (44.4 mg, 0.134 mmol, 98%) was obtained as a colorless solid.

*Synthesis of 3,4-dimethoxyphenyl methyl sulfide (**13a**)*

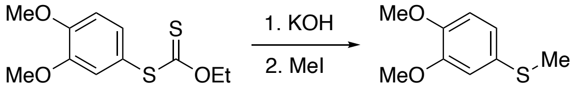

In a 5 mL screw-top V-vial® with a solid-top cap (Sigma–Aldrich, Cat. No. Z115118), to a mixture of *S*-(3,4-dimethoxyphenyl) *O*-ethyl carbonodithioate (**3a**) (33.3 mg, 0.129 mmol) and ethanol (0.400 mL) was added KOH (32.3 mg, 0.576 mmol, 4.5 equiv) at room temperature. The mixture was stirred at 80 °C (oil bath, bath temp. 80 °C) for 12 h. The resulting mixture was cooled to room temperature. To the mixture was added methyl iodide (25.0 µL, 402 µmol, 3.0 equiv) at room temperature. After stirring for 3 h at the same temperature, to the mixture was added ice-water (5 mL) and EtOAc (5 mL). The mixture was extracted with EtOAc (5 mL × 3). The combined organic extract was washed with brine (5 mL). The extract was dried with Na_2SO_4. After filtration, the filtrate was concentrated under reduced pressure to afford 3,4-dimethoxyphenyl methyl sulfide (**13a**) (23.4 mg, 127 µmol, 99%) as a colorless oil.

*Synthesis of 3,4-dimethoxyphenyl 2-nitrophenyl sulfide (**13b**)*

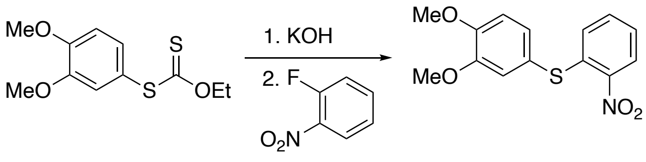

In a 5 mL screw-top V-vial® with a solid-top cap (Sigma–Aldrich, Cat. No. Z115118), to a mixture of *S*-(3,4-dimethoxyphenyl) *O*-ethyl carbonodithioate (**3a**) (33.9 mg, 0.129 mmol) and ethanol (0.400 mL) was added KOH (32.3 mg, 0.576 mmol, 4.3 equiv) at room temperature. After stirring for 24 h at 80 ºC (oil bath, bath temp. 80 °C), the mixture was cooled to room temperature. To the mixture was added 2-fluoronitrobenzene (42.3 µL, 402 µmol, 3.1 equiv) at room temperature. After stirring for 12 h at 100 ºC (oil bath, bath temp. 100 °C), the mixture was cooled to room temperature and was add water (5 mL) and EtOAc (5 mL). The mixture was extracted with EtOAc (5 mL × 3). The combined organic extract was washed with brine (5 mL). The extract was dried with Na_2SO_4. After filtration, the filtrate was concentrated under reduced pressure. The residue was purified by preparative TLC (*n*-hexane/EtOAc = 4/1) to give 3,4-dimethoxyphenyl 2-nitrophenyl sulfide (**13b**) (30.4 mg, 104 µmol, 80%) as a yellow solid.

*Synthesis of 3,4-dimethoxybenzenesulfonyl chloride (**14a**)*

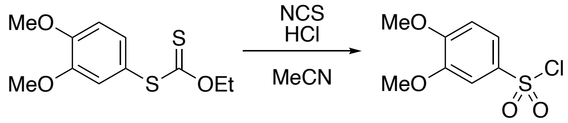

In a 5 mL screw-top V-vial® with a solid-top cap (Sigma–Aldrich, Cat. No. Z115118), to a mixture of 2 M aqueous HCl solution (72 µL) and CH_3CN (1.8 mL) was added *N*-chlorosuccinimide (72.5 mg, 0.54 mmol, 4.0 equiv) at room temperature. After the mixture was cooled to 10 °C, a solution of *S*-(3,4-dimethoxyphenyl) *O*-ethyl carbonodithioate (**3a**) (17.4 mg, 0.135 mmol) in CH_3CN (72 µL) was added dropwise to the mixture at the same temperature. After stirring for 1 h at the same temperature, to the mixture were added methyl *tert*-butyl ether (MTBE) (5 mL) and H_2O (5 mL). The mixture was extracted with MTBE (5 mL × 3). The combined organic extract was washed with H_2O (5 mL × 3). The extract was dried with Na_2SO_4. After filtration, the filtrate was concentrated under reduced pressure. The residue was purified by preparative TLC (*n*-hexane/EtOAc = 4/1) to give 3,4-dimethoxybenzenesulfonyl chloride (**14a**) (27.9 mg, 118 µmol, 87%) as a colorless solid.

*Synthesis of 3,4-dimethoxybenzenesulfonyl fluoride (**14b**)*

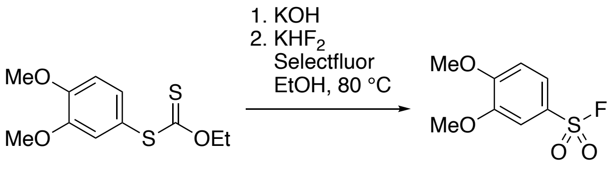

In a 5 mL screw-top V-vial® with a solid-top cap (Sigma–Aldrich, Cat. No. Z115118), to a mixture of *S*-(3,4-dimethoxyphenyl) *O*-ethyl carbonodithioate (**3a**) (28.4 mg, 0.110 mmol) and KOH (20.0 mg, 0.356 mmol, 3.2 equiv) was added ethanol (0.70 mL) at room temperature. The reaction mixture was stirred at 60 ºC (oil bath, bath temp. 60 °C) for 2 h. The mixture was cooled to room temperature. To the mixture was slowly added KHF_2 (26.0 mg, 0.330 mmol, 3.0 equiv) at room temperature. After stirring for 30 min at the same temperature, the solvent was removed under reduced pressure. Then, acetonitrile (0.50 mL), water (50 µL), and Selectfluor™ (127 mg, 0.358 mmol, 3.3 equiv) were added to the residue at room temperature. After stirring for 3 h at 80 °C (oil bath, bath temp. 80 °C), to the mixture was added water (2.5 mL). The mixture was extracted with EtOAc (5 mL × 3). The combined organic extract was washed with brine (5 mL), and dried over anhydrous Na_2SO_4. After filtration, the filtrate was concentrated under reduced pressure. The residue was purified by preparative TLC (*n*-hexane/EtOAc = 4/1) to give 3,4-dimethoxybenzenesulfonyl fluoride (**14b**) (18.4 mg, 83.5 µmol, 76%) as a colorless solid.

*Synthesis of 2-bromo-4,5-difluorophenyl 2-bromonaphthalen-1-yl sulfide (**16**)*

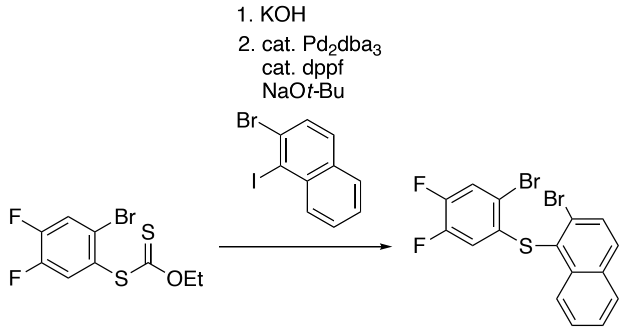

In a 5 mL screw-top V-vial® with a solid-top cap (Sigma–Aldrich, Cat. No. Z115118), to a mixture of S-(2-bromo-4,5-difluorophenyl) *O*-ethyl carbonodithioate (**8d**) (157 mg, 0.502 mmol, 1.0 equiv) and KOH (121 mg, 2.15 mmol, 4.3 equiv) was added ethanol (0.40 mL) at room temperature. After stirring 16 h at 80 ºC (oil bath, bath temp. 80 °C), the mixture was cooled to room temperature. To the mixture were added water (10 mL) and EtOAc (10 mL). The mixture was extracted with water (5 mL × 3). The combined aqueous extract was acidified with 1 M aqueous HCl solution (10 mL). The mixture was extracted with EtOAc (5 mL × 3). The combined organic extract was washed with brine (5 mL). The extract was dried with Na_2SO_4. After filtration, the filtrate was concentrated under reduced pressure. The crude product was used without purification.

In a 5 mL screw-top V-vial® with a solid-top cap (Sigma–Aldrich, Cat. No. Z115118), to the crude product in toluene (3.0 mL) were added Pd_2dba_3 (4.6 mg, 5.0 µmol, 1 mol %), dppf (55.2 mg, 0.10 mmol, 20 mol %), NaO*t*-Bu (57.7 mg, 0.600 mmol, 1.2 equiv), 2-bromo-1-iodonaphthalene (**15**) (165 mg, 0.495 mmol, 1.0 equiv) at room temperature. After stirring 6 h at 160 ºC (aluminium heating block), water (2.5 mL) was added to the resulting mixture. The mixture was extracted with EtOAc (5 mL × 3). The combined organic extract was washed with brine (5 mL). The extract was dried with Na_2SO_4. After filtration, the filtrate was concentrated under reduced pressure. The residue was purified by column chromatography (*n*-hexane/EtOAc = 4/1) to give 2-bromo-4,5-difluorophenyl 2-bromonaphthalen-1-yl sulfide (**16**) (168 mg, 0.391 mmol, 79%) as a colorless solid.

*Synthesis of 9,10-difluoro-7-phenyl-7H-benzo[c]phenothiazine (**17**)*

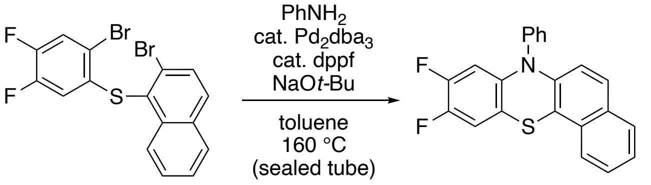

In a 5 mL screw-top V-vial® with a solid-top cap (Sigma–Aldrich, Cat. No. Z115118), a mixture of 2-bromo-4,5-difluorophenyl 2-bromonaphthalen-1-yl sulfide (**16**) (81.2 mg, 189 µmol), aniline (17.8 mg, 191 µmol, 1.0 equiv), Pd_2dba_3 (8.8 mg, 9.6 µmmol, 5 mol %), dppf (21.4 mg, 38.6 µmol, 20 mol %), and NaO*t*-Bu (74.5 mg, 0.775 mmol, 4.1 equiv) was added toluene (1.0 mL) at room temperature. After stirring 16 h at 160 ºC (aluminium heating block), water (2.5 mL) was added to the resulting mixture. The mixture was extracted with EtOAc (5 mL × 3). The combined organic extract was washed with brine (5 mL). The extract was dried with Na_2SO_4. After filtration, the filtrate was concentrated under reduced pressure. The residue was purified by column chromatography (*n*-hexane/EtOAc = 4/1) to give 9,10-difluoro-7-phenyl-7*H*-benzo[*c*]phenothiazine (**17**) (64.5 mg, 178 µmol, 95%) as a yellow solid.

*Synthesis of 9,10-difluorobenzo[a]thianthrene (**18**)*

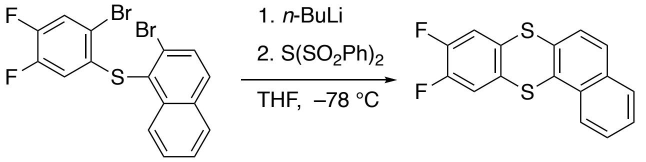

To (2-bromo-4,5-difluorophenyl)(2-bromonaphthalen-1-yl)sulfane (**16**) (20.1 mg, 46.0 µmol) was added THF (3.0 mL) at room temperature. After stirring for 15 min at –78 °C, to this was slowly added *n*-BuLi (1.6 M, THF solution, 63.3 µL, 101 µmol, 2.2 equiv) at –78 °C. After stirring for 1 h at the same temperature, to this was slowly added benzenesulfonic thioanhydride (19.0 mg, 60.4 µmol, 1.3 equiv). After stirring for 15 min at the same temperature, the mixture was warmed to room temperature. After stirring for 2 h at room temperature, to the mixture was slowly added water (5 mL). The mixture was extracted with EtOAc (5 mL × 3). The combined organic extract was washed with brine (5 mL). The extract was dried with Na_2SO_4. After filtration, the filtrate was concentrated under reduced pressure. The residue was purified by preparative TLC (*n*-hexane/EtOAc = 4/1) to afford 9,10-difluorobenzo[*a*]thianthrene (**18**) (7.6 mg, 25 µmol, 55%) as a colorless solid.

Characterization Data of New Compounds

O-Ethyl *S*-phenyl carbonodithioate (**3b**),[S16] *O*-ethyl *S*-(3-methoxyphenyl) carbonodithioate (**3e**),[S16] *S*-(2-bromophenyl) *O*-ethyl carbonodithioate (**8b**),[S16] bis(3,4-dimethoxyphenyl) disulfide (**12**),[S17] 3,4-dimethoxyphenyl methyl sulfide (**13a**),[S18] 3,4-dimethoxybenzenesulfonyl chloride (**14a**),[S19] and 3,4-dimethoxybenzenesulfonyl fluoride (**14b**)[S20] were identical in spectra data with those reported in the literature.

S-(3,4-Dimethoxyphenyl) *O*-ethyl carbonodithioate (**3a**)

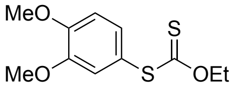

Yield: quant. (12.5 mg, 48.3 μmol); Colorless oil; TLC R_f 0.37 (*n*-hexane/EtOAc = 5/1); ^{1}H NMR ($CDCl_3$, 400 MHz): δ 7.09 (dd, 1H, *J* = 8.4, 2.0 Hz), 7.00 (d, 1H, *J* = 2.0 Hz), 6.91 (d, 1H, *J* = 8.4 Hz), 4.62 (q, 2H, *J* = 7.2 Hz), 3.92 (s, 3H), 3.89 (s, 3H), 1.35 (t, 3H, *J* = 7.2 Hz); ^{13}C{^{1}H} NMR ($CDCl_3$, 101 MHz): δ 214.4, 150.6, 149.0, 128.3, 122.1, 117.8, 111.2, 70.3, 56.0, 55.9, 13.7; IR (NaCl, cm^{-1}) 806, 850, 880, 1026, 1110, 1139, 1179, 1256, 1332, 1364, 1439, 1504, 1585, 2837, 2956; HRMS (ESI) *m/z*: [M+Na]$^+$ Calcd for $C_{11}H_{14}NaO_3S_2^+$ 281.0282; Found 281.0284.

S-(Benzo[*d*][1,3]dioxol-5-yl) *O*-ethyl carbonodithioate (**3c**)

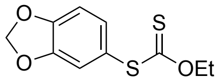

Yield: quant. (12.5 mg, 51.6 μmol); Pale yellow oil; TLC R_f 0.66 (*n*-hexane/EtOAc = 4/1); ^{1}H NMR ($CDCl_3$, 400 MHz): δ 7.00 (ddd, 1H, *J* = 8.0, 1.7 Hz), 6.95 (d, 1H, *J* = 1.7 Hz), 6.85 (d, 1H, *J* = 8.0 Hz), 6.04 (s, 2H), 4.62 (q, 2H, *J* = 7.1 Hz), 1.35 (t, 3H, *J* = 7.1 Hz);^{13}C{^{1}H} NMR ($CDCl_3$, 101 MHz): δ 214.4, 149.4, 148.1, 129.7, 122.2, 115.3, 108.9, 101.7, 70.4, 13.7; IR (NaCl, cm^{-1}) 801, 847, 936, 999, 1107, 1152, 1235, 1369, 1474, 1551, 2895, 2982; HRMS (ESI) *m/z*: [M+Na]$^+$ Calcd for $C_{10}H_{10}NaO_3S_2^+$ 264.9969; Found 264.9970.

S-(3,4-Difluorophenyl) *O*-ethyl carbonodithioate (**3d**)

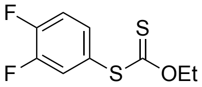

Yield: 96% (11.2 mg, 47.8 μmol); Pale yellow oil; TLC R_f 0.76 (*n*-hexane/EtOAc = 4/1); ^{1}H NMR ($CDCl_3$, 400 MHz): δ 7.36 (dddd, 1H, *J* = 8.6, 8.6, 1.2, 1.2 Hz), 7.27–7.19 (m, 2H), 4.62 (q, 2H, *J* = 7.1 Hz), 1.35 (t, 3H, *J* = 7.1 Hz); ^{13}C{^{1}H} NMR ($CDCl_3$, 101 MHz):δ 211.9, 151.7 (dd, *J* = 256.7, 11.6 Hz), 149.7 (dd, *J* = 254.7, 13.5 Hz), 131.9 (dd, *J* = 6.5 Hz), 126.3 (d, *J* = 6.5 Hz), 124.4 (dd, *J* = 18.6, 1.4 Hz), 118.0 (d, *J* = 17.7 Hz), 70.7, 13.6; ^{19}F{^{1}H} NMR ($CDCl_3$, 377 MHz): δ –134.1 (d, 1F, *J* = 20.7 Hz), –135.5 (d, 1F, *J* = 20.7 Hz); IR (NaCl, cm^{-1}) 816, 904, 1042, 1112, 1149, 1205, 1232, 1275, 1368, 1407, 1504, 1605, 2986; HRMS (EI) *m/z*: [M]$^{•+}$ Calcd for $C_9H_8F_2OS_2^{•+}$ 233.9985; Found 233.9983.

S-(3-Azido-5-methoxyphenyl) *O*-ethyl carbonodithioate (**3f**)

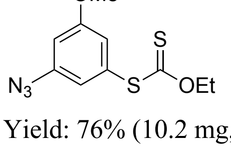

Yield: 76% (10.2 mg, 37.9 μmol); Brown oil; TLC R_f 0.68 (*n*-hexane/Ethyl acetate = 4/1); ^{1}H NMR ($CDCl_3$, 400 MHz): δ 6.84–6.80 (m, 2H), 6.61 (dd, 1H, *J* = 2.1, 2.1 Hz), 4.63 (q, 2H, *J* = 7.1 Hz), 3.82 (s, 3H), 1.36 (t, 3H, *J* = 7.1 Hz); ^{13}C{^{1}H} NMR ($CDCl_3$, 101 MHz): δ 211.8, 160.8, 141.9, 132.3, 117.6, 116.8, 106.8, 70.5, 55.7, 13.6; IR (NaCl, cm^{-1}) 847, 931, 990, 1002, 1040, 1110, 1122, 1142, 1232, 1322, 1366, 1459, 1589, 2109, 2962; HRMS (ESI) *m/z:* [M+Na]$^+$ Calcd for $C_{10}H_{11}N_3NaO_2S_2^+$ 292.0190; Found 292.0190.

S-(3-Bromophenyl) *O*-ethyl carbonodithioate (**3g**)

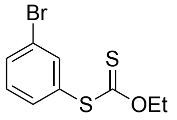

Yield: 85% (11.5 mg, 41.5 µmol); Pale brown oil; TLC R_f 0.78 (*n*-hexane/EtOAc = 4/1); ^{1}H NMR ($CDCl_3$, 400 MHz): δ 7.67 (dd, 1H, J = 1.6, 1.6 Hz), 7.58 (ddd, 1H, J = 8.0, 1.6, 1.2 Hz), 7.44 (ddd, 1H, J = 8.0, 1.6, 1.2 Hz), 7.31 (dd, 1H, J = 8.0, 8.0 Hz), 4.62 (q, 2H, J = 7.2 Hz), 1.35 (t, 3H, J = 7.2 Hz); ^{13}C{^{1}H} NMR ($CDCl_3$, 101 MHz): δ 211.7, 137.6, 133.6, 133.0, 132.0, 130.5, 122.6, 70.5, 13.5; IR (NaCl, cm^{-1}) 851, 954, 996, 1040, 1113, 1148, 1231, 1291, 1365, 1401, 1445, 1564, 2925; HRMS (EI) *m/z*: $[M]^{\bullet+}$ Calcd for $C_9H_9BrOS_2^{\bullet+}$ 275.9278; Found 275.9277.

S-(3-Chlorophenyl) *O*-ethyl carbonodithioate (**3h**)

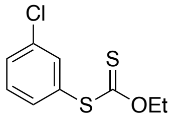

Yield: 84% (9.8 mg, 42 µmol); Colorless oil; TLC R_f 0.83 (*n*-hexane/Ethyl acetate = 4/1); ^{1}H NMR ($CDCl_3$, 400 MHz): δ 7.52 (d, 1H, J = 1.2 Hz), 7.45–7.34 (m, 3H), 4.62 (q, 2H, J = 7.2 Hz), 1.34 (t, 3H, J = 7.2 Hz); ^{13}C{^{1}H} NMR ($CDCl_3$, 101 MHz): δ 211.8, 134.8, 134.6, 133.1, 131.7, 130.2, 130.1, 70.5, 13.5; IR (NaCl, cm^{-1}) 996, 1040, 1072, 1085, 1110, 1120, 1149, 1229, 1291, 1366, 1403, 1462, 1567, 1574, 1733, 2982; HRMS (EI) *m/z*: $[M]^{\bullet+}$ Calcd for $C_9H_9ClOS_2^{\bullet+}$ 231.9783; Found 231.9784.

O-Ethyl *S*-(3-morpholinophenyl) carbonodithioate (**3i**)

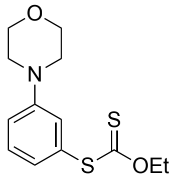

Yield: 71% (10.3 mg, 36.3 µmol); Yellow oil; TLC R_f 0.43 (*n*-hexane/EtOAc = 4/1); ^{1}H NMR ($CDCl_3$, 400 MHz): δ 7.32 (dd, 1H, J = 8.0, 8.0 Hz), 7.05–6.96 (m, 3H), 4.62 (q, 2H, J = 7.1 Hz), 3.89–3.83 (AA'BB', 4H), 3.21–3.15 (AA'BB', 4H), 1.34 (t, 3H, J = 7.1 Hz); ^{13}C{^{1}H} NMR ($CDCl_3$, 101 MHz): δ 213.4, 151.8, 130.8, 129.7, 126.1, 121.9, 117.0, 70.2, 66.7, 48.9, 13.6; IR (NaCl, cm^{-1}) 947, 987, 1002, 1040, 1122, 1148, 1232, 1288, 1365, 1379, 1484, 1590, 2827, 2855, 2962; HRMS (ESI) *m/z*: $[M+H]^+$ Calcd for $C_{13}H_{18}NO_2S_2^+$ 284.0779; Found 284.0779.

O-Ethyl *S*-(3-((4-methoxyphenyl)thio)phenyl) carbonodithioate (**3j**)

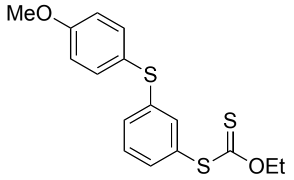

Yield: 95% (15.9 mg, 47.2 µmol); Pale yellow oil; TLC R_f 0.57 (*n*-hexane/EtOAc = 4/1); ^{1}H NMR ($CDCl_3$, 400 MHz): δ 7.46–7.41 (AA'BB', 2H), 7.30–7.18 (m, 4H), 6.95–6.89 (AA'BB', 2H), 4.58 (q, 2H, J = 7.1 Hz), 3.83 (s, 3H), 1.31 (t, 3H, J = 7.1 Hz); ^{13}C{^{1}H} NMR ($CDCl_3$, 101 MHz): δ 212.4, 160.1, 140.3, 135.8, 133.7, 131.9, 130.8, 129.4, 129.1,123.0, 115.1, 70.3, 55.4, 13.6; IR (NaCl, cm^{-1}) 828, 994, 1006, 1029, 1109, 1143, 1173, 1228, 1246, 1389, 1462, 1494, 1581, 1593; HRMS (ESI) *m/z*: $[M+Na]^+$ Calcd for $C_{16}H_{16}NaO_2S_3^+$ 359.0210; Found 359.0210.

O-Ethyl *S*-(naphthalen-2-yl) carbonodithioate (**3k**)

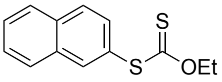

Yield: 84% (13.7 mg, 83.7 μmol); Colorless solid; TLC R_f 0.73 (*n*-hexane/EtOAc = 4/1); Mp 56–57 ºC; ^{1}H NMR ($CDCl_3$, 400 MHz): δ 7.81–7.71 (m, 4H), 7.49–7.39 (m, 3H), 3.05 (q, 2H, *J* = 7.4 Hz), 1.37 (t, 3H, *J* = 7.4 Hz); $^{13}C\{^1H\}$ NMR ($CDCl_3$, 101 MHz): δ 188.6, 134.1, 133.8, 131.6, 128.3, 127.7, 127.2, 127.0, 126.5 (two signals overlapped), 125.5, 27.5, 14.3; IR (NaCl, cm^{-1}) 850, 863, 959, 1052, 1109, 1148, 1223, 1246, 1366, 3010; HRMS (EI) *m/z*: $[M]^{\bullet+}$ Calcd for $C_{13}H_{12}OS_2^{\bullet+}$ 248.0330; Found 248.0330.

O-Ethyl *S*-(pyridin-2-yl) carbonodithioate (**3l**)

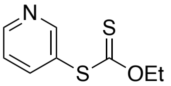

Yield: 83% (8.3 mg, 42 μmol); Brown oil; TLC R_f 0.43 (*n*-hexane/Ethyl acetate = 4/1); ^{1}H NMR ($CDCl_3$, 400 MHz): δ 8.68 (d, 1H, *J* = 4.8 Hz), 7.77 (ddd, 1H, *J* = 7.5, 7.5, 1.6 Hz), 7.65 (d, 1H, *J* = 7.6 Hz), 7.37–7.32 (m, 1H), 4.62 (q, 2H, *J* = 7.2 Hz), 1.34 (t, 3H, *J* = 7.2 Hz); $^{13}C\{^1H\}$ NMR ($CDCl_3$, 101 MHz): δ 211.0, 152.7, 150.6, 137.4, 131.0, 124.0, 70.4, 13.5; IR (NaCl, cm^{-1}) 850, 989, 1000, 1036, 1087, 1109, 1123, 1149, 1229, 1273, 1421, 1449, 1573, 2358, 2982; HRMS (ESI) *m/z*: $[M+Na]^+$ Calcd for $C_8H_9NNaOS_2^+$ 222.0023; Found 222.0024.

O-Ethyl *S*-(1-methyl-1*H*-indol-6-yl) carbonodithioate (**3m**)

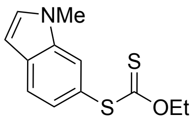

Yield: 98% (6.8 mg, 27 μmol); Pale brown oil; TLC R_f 0.53 (*n*-hexane/Ethyl acetate = 4/1); ^{1}H NMR ($CDCl_3$, 400 MHz): δ 7.65 (d, 1H, *J* = 8.2 Hz), 7.50 (s, 1H), 7.22 (dd, 1H, *J* = 8.2, 1.2 Hz), 7.14 (d, 1H, *J* = 3.1 Hz), 6.52 (dd, 1H, *J* = 3.1, 1.2 Hz), 4.62 (q, 2H, *J* = 7.1 Hz), 3.81 (s, 3H), 1.32 (t, 3H, *J* = 7.1 Hz); $^{13}C\{^1H\}$ NMR ($CDCl_3$, 101 MHz): δ 215.3, 136.7, 130.8, 129.6, 126.0, 122.0, 121.4, 116.4, 101.3, 70.2, 33.0, 13.6; IR (NaCl, cm^{-1}) 808, 853, 906, 1003, 1082, 1145, 1235, 1322, 1338, 1418, 1505, 1607; HRMS (ESI) *m/z*: $[M+Na]^+$ Calcd for $C_{12}H_{13}NNaOS_2^+$ 274.0336; Found 274.0338.

S-(2-Bromo-4,5-dimethoxyphenyl) *O*-ethyl carbonodithioate (**8a**)

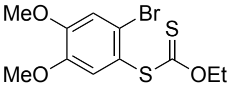

Yield: 86% (14.6 mg, 43.2 μmol); Colorless solid; Mp 75–76 ºC; TLC R_f 0.48 (*n*-hexane/EtOAc = 4/1); ^{1}H NMR ($CDCl_3$, 400 MHz): δ 7.17 (s, 1H), 7.07 (s, 1H), 4.63 (q, 2H, *J* = 7.0 Hz), 3.92 (s, 3H), 3.87 (s, 3H), 1.35 (t, 3H, *J* = 7.1 Hz); $^{13}C\{^1H\}$ NMR ($CDCl_3$, 101 MHz): δ 211.6, 151.2, 148.5, 122.2, 121.8, 118.9, 115.8, 70.5, 56.2 (two signals overlapped), 13.7; IR (NaCl, cm^{-1}) 854, 909, 1043, 1148,1179, 1211, 1229, 1289, 1355, 1464, 1500, 1584, 2959; HRMS (ESI) *m/z*: $[M+Na]^+$ Calcd for $C_{11}H_{13}BrNaO_3S_2^+$ 358.9387; Found 358.9385.

S-(6-Bromobenzo[*d*][1,3]dioxol-5-yl) *O*-ethyl carbonodithioate (**8c**)

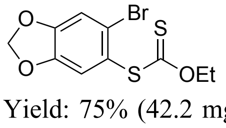

Yield: 75% (42.2 mg, 150 μmol); Colorless oil; TLC R_f 0.61 (*n*-hexane/EtOAc = 4/1); ^{1}H NMR ($CDCl_3$, 400 MHz): δ 7.16 (s, 1H), 7.05 (s, 1H), 6.06 (s, 2H), 4.62 (q, 2H, *J* = 7.1 Hz), 1.36 (t, 3H, *J* = 7.1 Hz); $^{13}C\{^1H\}$ NMR ($CDCl_3$, 101 MHz): δ 211.5, 150.3, 147.6, 123.2, 122.7, 116.1, 113.4, 102.5, 70.5, 13.6; IR (NaCl, cm^{-1}) 807, 891, 934, 1003, 1037, 1107, 1150, 1172, 1236, 1296, 1422, 1478, 1502, 2898; HRMS (ESI) *m/z*: $[M+Na]^+$ Calcd for $C_{10}H_9BrNaO_3S_2^+$ 342.9074; Found 342.9075.

S-(3-Bromonaphthalen-2-yl) *O*-ethyl carbonodithioate (**8d**)

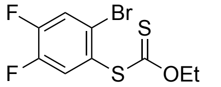

Yield: 61% (19.2 mg, 61.3 μmol); Colorless oil; TLC R_f 0.81 (*n*-hexane/EtOAc = 4/1); ^{1}H NMR ($CDCl_3$, 400 MHz): δ 7.56 (dd, 1H, *J* = 9.5, 7.4 Hz), 7.49 (dd, 1H, *J* = 9.5, 7.4 Hz), 4.62 (q, 2H, *J* = 7.2 Hz), 1.36 (t, 3H, *J* = 7.2 Hz); ^{13}C{^{1}H} NMR ($CDCl_3$, 101 MHz): δ 209.2, 151.5 (dd, *J* = 259.1, 13.3 Hz), 149.3 (dd, *J* = 254.1, 13.0 Hz), 127.9 (dd, *J* = 5.8, 4.6 Hz), 125.6 (*J* = 18.4 Hz), 125.1 (dd, *J* = 7.4, 3.8 Hz),122.4 (d, *J* = 20.4 Hz), 70.9, 13.6; ^{19}F{^{1}H} NMR ($CDCl_3$, 377 MHz): δ –136.4 (d, 1F, *J* = 22.6 Hz), –130.7 (d, 1F, *J* = 22.6 Hz); IR (NaCl, cm^{-1}) 813, 854, 873, 1042, 1076, 1112, 1150, 1178, 1203, 1239, 1275, 1368, 1399, 1404, 1457, 1475, 1504, 1594, 1601; HRMS (EI) *m/z*: $[M]^{\bullet+}$ Calcd for $C_9H_8F_2OS_2^{\bullet+}$ 233.9983; Found 233.9983.

S-(2-Bromo-3-methoxyphenyl) *O*-ethyl carbonodithioate (**8e**)

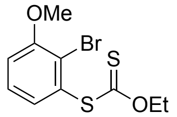

Yield: 74% (23.3 mg, 75.7 μmol); Colorless solid; Mp 82–83 ºC; TLC R_f 0.55 (*n*-hexane/EtOAc = 4/1); ^{1}H NMR ($CDCl_3$, 400 MHz): δ 7.33 (dd, 1H, *J* = 8.0, 8.0 Hz), 7.25 (dd, 1H, *J* = 8.0, 1.2 Hz), 6.99 (dd, 1H, *J* = 8.0, 1.2 Hz), 4.61 (q, 2H, *J* = 7.2 Hz), 3.94 (s, 3H), 1.32 (t, 3H, *J* = 7.2 Hz); ^{13}C{^{1}H} NMR ($CDCl_3$, 101 MHz): δ 210.6, 157.0, 132.9, 128.8, 128.3, 119.9, 113.4, 70.4, 56.6, 13.6; IR (NaCl, cm^{-1}) 1020, 1040, 1110, 1150, 1185, 1189, 1226, 1265, 1418, 1424, 1431, 1435, 1457, 1462, 1558, 1564, 1568, 1575; HRMS (ESI) *m/z*: $[M+Na]^+$ Calcd for $C_{10}H_{11}BrNaO_2S_2^+$ 328.9282; Found 328.9282.

S-(5-Azido-2-bromo-3-methoxyphenyl) *O*-ethyl carbonodithioate (**8f**)

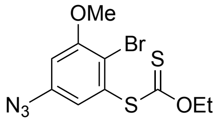

Yield: 55% (19.0 mg, 54.6 μmol); Brown solid; Mp 82–83 ºC; TLC R_f 0.70 (*n*-hexane/EtOAc = 4/1); ^{1}H NMR ($CDCl_3$, 400 MHz): δ 6.98 (d, 1H, *J* = 2.4 Hz), 6.58 (d, 1H, *J* = 2.4 Hz), 4.62 (q, 2H, *J* = 7.2 Hz), 3.92 (s, 3H), 1.34 (t, 3H, *J* = 7.2 Hz); ^{13}C{^{1}H} NMR ($CDCl_3$, 101 MHz): δ 209.5, 157.8, 140.5, 133.9, 118.5, 115.5, 104.6, 70.6, 56.7, 13.6; IR (NaCl, cm^{-1}) 846, 962, 1045, 1128, 1249, 1311, 1407, 1424, 1577, 2109; HRMS (ESI) *m/z*: $[M+Na]^+$ Calcd for $C_{10}H_{10}BrN_3NaO_2S_2^+$ 369.9290; Found 369.9294.

S-(2,3-Dibromophenyl) *O*-ethyl carbonodithioate (**8g**)

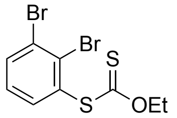

Yield: 23% (8.3 mg, 23 μmol); Colorless oil; TLC R_f 0.75 (*n*-hexane/EtOAc = 4/1); ^{1}H NMR ($CDCl_3$, 400 MHz): δ 7.33 (dd, 1H, *J* = 8.0, 8.0 Hz), 7.25 (dd, 1H, *J* = 8.0, 1.2 Hz), 6.99 (dd, 1H, *J* = 8.0, 1.2 Hz), 4.61 (q, 2H, *J* = 7.2 Hz), 1.32 (t, 3H, *J* = 7.2 Hz); ^{13}C{^{1}H} NMR ($CDCl_3$, 101 MHz): δ 209.8, 135.9, 135.5, 133.9, 132.9, 128.7, 126.6, 70.7, 13.6; IR (NaCl, cm^{-1}) 838, 990, 1018, 1107, 1128, 1196, 1251, 1385, 1427, 1471, 1563, 2921, 2976; HRMS (EI) *m/z*: $[M]^{\bullet+}$ Calcd for $C_9H_8Br_2OS_2^{\bullet+}$ 354.8462; Found 354.8461.

S-(2-Bromo-3-((4-methoxyphenyl)thio)phenyl) *O*-ethyl carbonodithioate (**8h**)

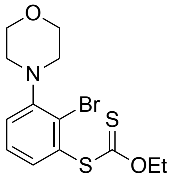

Yield: 72% (26.2 mg, 72.3 μmol); Pale yellow oil; TLC R_f 0.26 (*n*-hexane/EtOAc = 4/1); ^{1}H NMR ($CDCl_3$, 400 MHz): δ 7.39–7.32 (m, 2H), 7.15 (dd, 1H *J* = 7.2, 2.0 Hz), 4.60 (q, 2H, *J* = 7.2 Hz), 3.90–3.84 (AA'BB', 4H),

3.18–3.12 (AA'BB', 4H), 1.32 (t, 3H, J = 7.2 Hz); $^{13}C\{^{1}H\}$ NMR ($CDCl_3$, 101 MHz): δ 210.8, 152.1, 133.3, 131.8, 128.3, 128.3, 122.7, 70.3, 67.1, 52.2, 13.6; IR (NaCl, cm^{-1}) 947, 987, 1002, 1120, 1147, 1229, 1364, 1378, 1448, 1484, 1588, 2827, 2962; HRMS (ESI) *m/z*: $[M+Na]^+$ Calcd for $C_{13}H_{16}BrNNaO_2S_2^+$ 383.9698; Found 383.9702.

S-(2-Bromo-3-((4-methoxyphenyl)thio)phenyl) *O*-ethyl carbonodithioate (**8i**)

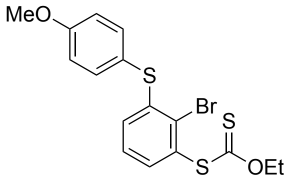

Yield: 67% (14.1 mg, 33.9 μmol); Pale yellow solid; Mp 96–99 °C; TLC R_f 0.45 (*n*-hexane/EtOAc = 4/1); ^{1}H NMR ($CDCl_3$, 400 MHz): δ 7.52–7.47 (AA'BB', 2H), 7.34 (dd, 1H, J = 8.0, 1.2 Hz), 7.12 (dd, 1H, J = 8.0, 8.0 Hz), 7.01–6.96 (AA'BB', 2H), 6.80 (dd, 1H, J = 8.0, 1.2 Hz), 4.62 (q, 2H, J = 7.2 Hz), 3.87 (s, 3H), 1.34 (t, 3H, J = 7.2 Hz); $^{13}C\{^{1}H\}$ NMR ($CDCl_3$, 101 MHz): δ 210.4, 160.9, 143.8, 137.3, 133.0, 132.4, 128.4, 127.8, 127.5, 121.8, 115.5, 70.5, 55.4, 13.6; IR (NaCl, cm^{-1}) 828, 994, 1006, 1109, 1142, 1182, 1225, 1289, 1462, 1494, 1593, 2982; HRMS (ESI) *m/z*: $[M+Na]^+$ Calcd for $C_{16}H_{15}BrNaO_2S_3^+$ 436.9315; Found 436.9318.

S-(7-Bromo-1-methyl-1*H*-indol-6-yl) *O*-ethyl carbonodithioate (**8j**)

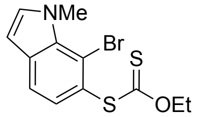

Yield: 67% (11.3 mg, 34.2 μmol); Colorless solid; Mp 104–105 ºC; TLC R_f 0.53 (*n*-hexane/EtOAc = 5/1); ^{1}H NMR ($CDCl_3$, 400 MHz): δ 7.55 (d, 1H, J = 8.1 Hz), 7.30 (d, 1H, J = 8.1 Hz), 7.10 (d, 1H, J = 3.1 Hz), 6.49 (d, 1H, J = 3.1 Hz), 4.62 (q, 2H, J = 7.1 Hz), 4.21 (s, 3H), 1.32 (t, 3H, J = 7.1 Hz); $^{13}C\{^{1}H\}$ NMR ($CDCl_3$, 101 MHz): δ 212.9, 134.2, 132.9, 127.8, 124.5, 120.5, 118.6, 113.0, 101.3, 70.3, 37.9, 13.7; IR (NaCl, cm^{-1}) 924, 1040, 1130, 1314, 1410, 1457, 1472, 1507, 1541, 2991; HRMS (ESI) *m/z*: $[M+Na]^+$ Calcd for $C_{12}H_{12}BrNNaOS_2^+$ 351.9441; Found 351.9440.

S-(2-Bromo-4,5-dimethoxyphenyl) *O*-(4-chlorophenethyl) carbonodithioate (**8k**)

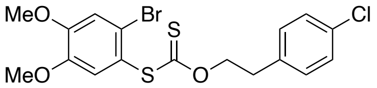

Yield: 55% (12.3 mg, 27.5 μmol); Colorless solid; Mp 74–76 ºC; TLC R_f 0.52 (*n*-hexane/EtOAc = 4/1); ^{1}H NMR ($CDCl_3$, 400 MHz): δ 7.19–7.14 (m, 3H), 6.98 (s, 1H), 6.92–6.89 (AA'BB', 2H), 4.67 (t, 2H, J = 6.4 Hz), 3.95 (s, 3H), 3.83 (s, 3H), 2.95 (t, 2H, J = 6.4 Hz); $^{13}C\{^{1}H\}$ NMR ($CDCl_3$, 101 MHz): δ 211.1, 151.3, 148.5, 135.6, 132.4, 130.1, 128.5, 121.7, 121.7, 118.7, 115.7, 74.0, 56.3, 56.2, 33.8; IR (NaCl, cm^{-1}) 813, 854, 873, 1042, 1076, 1112, 1150, 1178, 1239, 1275, 1399, 1404, 1475, 1504, 1594; HRMS (ESI) *m/z*: $[M+Na]^+$ Calcd for $C_{17}H_{16}BrClNaO_3S_2^+$ 468.9311; Found 468.9312.

O-Benzyl *S*-(2-bromo-4,5-dimethoxyphenyl) carbonodithioate (**8l**)

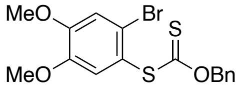

Yield: 49% (9.7 mg, 24 μmol); Colorless oil; TLC R_f 0.39 (*n*-hexane/EtOAc = 4/1); ^{1}H NMR ($CDCl_3$, 400 MHz): δ 7.36–7.31 (m, 3H), 7.30–7.26 (AA'BB'C, 2H), 7.15 (s, 1H), 7.06 (s, 1H), 5.59 (s, 2H), 3.91 (s, 3H), 3.81 (s, 3H); $^{13}C\{^{1}H\}$ NMR ($CDCl_3$, 101 MHz): δ 211.4, 151.3, 148.5, 134.3, 128.6, 128.5, 128.2, 122.0, 121.6, 119.0, 115.8, 75.4, 56.3, 56.2; IR (NaCl, cm^{-1}) 857, 909, 1047, 1126, 1179, 1211, 1253, 1325, 1355, 1437, 1455, 1464, 1584, 2932; HRMS (ESI) *m/z*: $[M+Na]^+$ Calcd for $C_{16}H_{15}BrNaO_3S_2^+$ 420.9544; Found 420.9543.

S-(2-Bromo-4,5-dimethoxyphenyl) *O*-isopropyl carbonodithioate (**8m**)

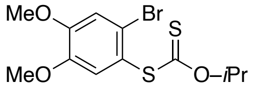

Yield: 50% (9.1 mg, 26 μmol);Yellow oil; TLC R_f 0.41 (*n*-hexane/EtOAc = 4/1); ^{1}H NMR ($CDCl_3$, 400 MHz): δ 7.16 (s, 1H), 7.01 (s, 1H), 5.69 (septet, 1H, *J* = 6.4 Hz), 3.92 (s, 3H), 3.87 (s, 3H), 1.33 (d, 6H, *J* = 6.4 Hz); ^{13}C{^{1}H} NMR ($CDCl_3$, 101 MHz): δ 210.8, 151.1, 148.4, 122.2, 121.8, 118.8, 115.7, 78.7, 56.2 (two peaks overlapped), 21.2; IR (NaCl, cm^{-1}) 804, 827, 876, 891, 899, 1020, 1090, 1107, 1239, 1368, 1392, 1441, 1455, 1472, 1501; HRMS (ESI) *m/z*: [M+H]$^+$ Calcd for $C_{12}H_{16}BrO_3S_2$ 350.9719; Found 350.9730.

S-(2-Bromo-4,5-dimethoxyphenyl) *O*-(pent-4-en-1-yl) carbonodithioate (**8n**)

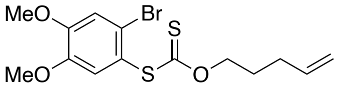

Yield: 53% (20.3 mg, 53.8 μmol);Colorless oil; TLC R_f 0.41 (*n*-hexane/EtOAc = 4/1); ^{1}H NMR ($CDCl_3$, 400 MHz): δ 7.17 (s, 1H), 7.08 (s, 1H), 5.79–5.67 (m, 1H), 5.02–4.94 (m, 2H), 4.55 (t, 2H, *J* = 6.4 Hz), 3.92 (s, 3H), 3.88 (s, 3H), 2.07–2.00 (m, 2H), 1.83–1.74 (m, 2H); ^{13}C{^{1}H} NMR ($CDCl_3$, 101 MHz): δ 211.5, 151.2, 148.5, 137.1, 122.1, 121.8, 118.8, 115.74, 115.66, 73.7, 56.32, 56.28, 29.9, 27.4; IR (NaCl, cm^{-1}) 857, 910, 1046, 1126, 1179, 1211, 1252, 1314, 1354, 1437, 1464, 1500, 1584; HRMS (ESI) *m/z*: [M+Na]$^+$ Calcd for $C_{14}H_{17}BrNaO_3S_2^+$ 398.9700; Found 398.9704.

S-(2-Bromo-4,5-dimethoxyphenyl) *N*,*N*-diethylcarbamodithioate (**8o**)

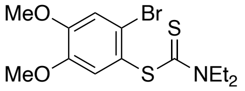

Yield: 71% (13.0 mg, 35.6 μmol); Colorless solid; Mp 106–108 ºC; TLC R_f 0.62 (*n*-hexane/CH_2Cl_2 = 1/1); ^{1}H NMR ($CDCl_3$, 400 MHz): δ 7.18 (s, 1H), 7.07 (s, 1H), 4.04 (q, 2H, *J* = 4.8 Hz), 3.92–3.83 (m, 8H), 1.43 (t, 3H, *J* = 7.0 Hz), 1.30 (t, 3H, *J* = 7.0 Hz); ^{13}C{^{1}H} NMR ($CDCl_3$, 101 MHz): δ 194.1, 151.1, 148.4, 123.5, 123.3, 120.9, 115.8, 56.2, 56.1, 49.9, 47.4, 12.9, 11.6; IR (NaCl, cm^{-1}) 910, 979, 1026, 1178, 1209, 1253, 1268, 1355, 1417, 1437, 1462, 1495, 1584, 2933; HRMS (ESI) *m/z*: [M+Na]$^+$ Calcd for $C_{13}H_{18}BrNNaO_2S_2^+$ 385.9860; Found 385.9860.

2-Bromo-4,5-dimethoxyphenyl benzyl(methyl)carbamodithioate (**8p**)

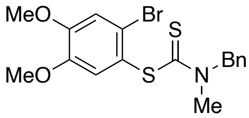

Yield: 80 % (16.8 mg, 40.7 μmol); Colorless oil; TLC R_f 0.48 (*n*-hexane/CH_2Cl_2 = 1/1); A mixture of rotational isomers was observed in ^{1}H and ^{13}C NMR spectra: ^{1}H NMR ($CDCl_3$, 400 MHz):δ 7.48–7.30 (m, 5H), 7.22 (s, 1H), 7.13–7.06 (m, 1H), 5.40 and 5.14 (two br s, total 2H), 3.96–3.88 (m, 6H), 3.50 and 3.42 (two br s, total 3H); ^{13}C{^{1}H} NMR ($CDCl_3$, 101 MHz): δ 197.4, 196.2, 151.3, 148.5, 135.5, 134.6, 129.0, 128.8, 128.1, 127.8, 123.7, 123.6, 123.4, 123.2, 120.9, 120.7, 115.8, 59.8, 58.4, 56.23, 56.18, 43.6, 39.2 (almost twice ^{13}C signals were observed due to the presence of a rotational isomer); IR (NaCl, cm^{-1}) 986 , 1026, 1178, 1209, 1253, 1324, 1654, 1385, 1435, 1464, 1470, 1481, 1488, 1495, 1584; HRMS (ESI) *m/z*: [M+Na]$^+$ Calcd for $C_{17}H_{18}BrNNaO_2S_2^+$ 433.9860; Found 433.9861.

O-Ethyl *S*-(2-iodo-4,5-dimethoxyphenyl) carbonodithioate (**10**)

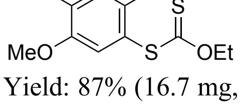

Yield: 87% (16.7 mg, 43.5 μmol); Pale brown oil; TLC R_f 0.45 (*n*-hexane/EtOAc = 4/1); ^{1}H NMR ($CDCl_3$, 400 MHz): δ 7.37 (s, 1H), 7.12 (s, 1H), 4.63 (q, 2H, *J* = 7.2 Hz), 3.91 (s, 3H), 3.87 (s, 3H), 1.36 (t, 3H, *J* = 7.2 Hz); ^{13}C{^{1}H} NMR ($CDCl_3$, 101 MHz): δ 211.8, 150.7, 149.4, 126.8, 121.9, 118.3, 97.5, 70.5, 56.2, 56.1, 13.7; IR (NaCl, cm^{-1}) 856, 1042, 1110, 1143, 1179, 1309, 1231, 1252, 1319, 1348, 1435, 1462, 1495, 1580; HRMS (ESI) *m/z*: [M+Na]$^+$ Calcd for $C_{11}H_{13}INaO_3S_2^+$ 406.9249; Found 406.9245.

3,4-Dimethoxyphenyl 2-nitrophenyl sulfide (**13b**)

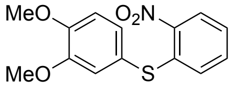

Yield: 80% (30.4 mg, 104 μmol); Pale yellow solid; Mp 139–140 °C; TLC R_f 0.36 (*n*-hexane/CH_2Cl_2 = 1/1); ^{1}H NMR ($CDCl_3$, 400 MHz): δ 8.23 (dd, 1H, *J* = 7.2, 1.3 Hz), 7.35 (ddd, 1H, *J* = 7.2, 7.2, 1.3 Hz), 7.24–7.17 (m, 2H), 7.00 (d, 1H, *J* = 1.8 Hz), 6.97 (d, 1H, *J* = 8.2 Hz), 6.86 (dd, 1H, *J* = 8.2, 1.8 Hz), 3.95 (s, 3H), 3.87 (s, 3H); ^{13}C{^{1}H} NMR ($CDCl_3$, 101 MHz): δ 150.8, 149.9, 144.5, 140.5, 133.4, 129.4, 127.8, 125.7, 124.7, 121.3, 118.2, 112.1, 56.05, 55.97; IR (NaCl, cm^{-1}) 853, 1026, 1103, 1139, 1233, 1256, 1336, 1451, 1462, 1504, 1585; HRMS (ESI) *m/z*: $[M+Na]^+$ Calcd for $C_{14}H_{13}NNaO_4S^+$ 314.0463; Found 314.0461.

2-Bromo-4,5-difluorophenyl 2-bromonaphthalen-1-yl sulfide (**16**)

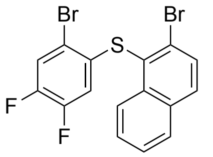

Yield: 79% (168.3 mg, 391 μmol); Colorless solid; Mp 71–73 °C; TLC R_f 0.65 (*n*-hexane/CH_2Cl_2 = 6/1); ^{1}H NMR ($CDCl_3$, 400 MHz): δ 8.44–8.38 (m, 1H), 7.96–7.81 (m, 3H), 7.63–7.54 (m, 2H), 7.43 (dd, 1H, *J* = 9.4, 7.4 Hz), 6.10 (dd, 1H, *J* = 10.8, 7.8 Hz); ^{13}C{^{1}H} NMR ($CDCl_3$, 101 MHz): δ 150.2 (dd, *J* = 229.0, 11.6 Hz), 147.7 (dd, *J* = 230.9, 12.9 Hz), 136.0, 134.9 (d, *J* = 4.3 Hz), 133.1, 132.4, 132.1, 130.7, 128.82, 128.78, 128.76, 127.0, 126.5, 121.7 (d, *J* = 20.5 Hz), 115.0 (d, *J* = 20.5 Hz), 113.8 (d, *J* = 3.8 Hz); ^{19}F{^{1}H} NMR ($CDCl_3$, 377 MHz): δ –137.1 (d, 1F, *J* = 20.9 Hz), –139.5 (d, 1F, *J* = 20.9 Hz); IR (NaCl, cm^{-1}) 811, 867, 934, 1092, 1175, 1215, 1255, 1201, 1368, 1281, 1450, 1554, 1581, 1592, 2925, 3063; HRMS (EI) *m/z*: $[M]^{\bullet+}$ Calcd for $C_{16}H_8Br_2F_2S^{\bullet+}$ 427.8682; Found 427.8684.

9,10-Difluoro-7-phenyl-7*H*-benzo[*c*]phenothiazine (**17**)

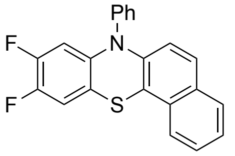

Yield: 95% (64.5 mg, 178 μmol); Yellow solid; TLC R_f 0.58 (*n*-hexane/CH_2Cl_2 = 5/1) Mp 126–127 ºC; ^{1}H NMR (Acetone-d_6, 400 MHz): δ 8.03 (d, 1H, *J* = 8.5 Hz), 7.87–7.73 (m, 3H), 7.68–7.56(m, 5H), 7.49–7.43 (AA’BB’C, 1H), 7.28 (ddd, 1H, *J* = 9.3, 9.3, 1.6 Hz), 6.71(d, 1H, *J* = 9.0 Hz), 6.32 (dd, 1H, *J* = 13.0, 7.2 Hz); ^{13}C{^{1}H} NMR (Acetone-d_6, 101 MHz): δ 150.5 (dd, *J* = 243.9, 13.3 Hz), 147.6 (dd, *J* = 243.1, 13.7 Hz), 143.9 (dd, *J* = 7.9, 2.6 Hz), 142.8, 142.6, 132.8, 132.1, 132.02, 131.99, 130.4, 129.9, 128.91, 128.88, 126.3, 123.9, 119.3, 118.1 (dd, *J* = 6.3, 3.5 Hz), 117.1 (d, *J* = 20.5 Hz), 114.0, 107.4 (d, *J* = 23.1 Hz); ^{19}F{^{1}H} NMR (Acetone-d_6, 377 MHz); δ –140.6 (d, 1F, *J* = 21.5 Hz), –148.0 (d, 1F, *J* = 21.5 Hz); IR (NaCl, cm^{-1}) 804, 880, 1153, 1239, 1275, 1399, 1457, 1469, 1472, 1504, 1593, 1603, 3057; HRMS (EI) *m/z*: $[M]^{\bullet+}$ Calcd for $C_{22}H_{13}F_2NS^{\bullet+}$ 361.0731; Found 361.0731.

9,10-Difluorobenzo[*a*]thianthrene (**18**)

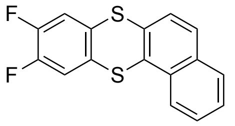

Yield: 55% (7.6 mg, 25 μmol); Colorless solid; TLC R_f 0.81 (*n*-hexane/EtOAc = 4/1) Mp 126–128 ºC; ^{1}H NMR ($CDCl_3$, 400 MHz): δ 8.42 (d, 1H, *J* = 8.3 Hz), 7.85 (d, 1H, *J* = 8.0 Hz), 7.76 (d, 1H, *J* = 8.5 Hz), 7.61 (ddd, 1H, *J* = 8.4, 8.4, 1.4 Hz), 7.57–7.50 (m, 2H), 7.44 (dd, 1H, *J* = 9.7, 7.4 Hz), 7.37 (dd, 1H, *J* = 9.7, 7.4 Hz);^{13}C{^{1}H} NMR (Acetone-d_6, 101 MHz): δ 150.0 (dd, *J* = 245.2, 6.0 Hz), 149.9 (dd, *J* = 245.2, 6.0 Hz), 133.3 (dd, *J* = 3.5, 3.5 Hz), 133.2, 132.9, 132.03, 131.98, 131.6 (d, *J* = 3.5, 3.5 Hz), 128.4, 128.2, 127.4, 126.6, 126.1, 124.1, 117.7 (d, *J* = 19.5 Hz), 117.1 (d, *J* = 19.5 Hz); ^{19}F{^{1}H} NMR ($CDCl_3$, 377 MHz): δ –137.5 (d, 1F, *J* = 20.8 Hz), –137.9

(d, 1F, J = 20.8 Hz); IR (NaCl, cm^{-1}) 867, 1027, 1046, 1057, 1073, 1082, 1125, 1280, 1468, 1720, 1757, 2347, 2922; HRMS (EI) *m/z*: $[M]^{\bullet+}$ Calcd for $C_{16}H_8F_2S_2^{\bullet+}$ 302.0036; Found 302.0037.

Potassium *O*-(4-chlorophenethyl) carbonodithioate (**2e**)

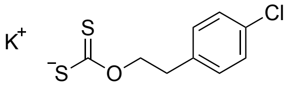

Yield: 50% (301 mg, 996 µmol); Colorless solid; Mp 192–194 ºC; ^{1}H NMR (DMSO-d_6, 400 MHz): δ 7.39–7.32 (m, 4H), 4.41 (t, 2H, J = 7.2 Hz), 2.94 (t, 2H, J = 7.2 Hz); ^{13}C{^{1}H} NMR (DMSO-d_6, 101 MHz): δ 230.4, 139.1, 131.7, 131.6, 129.1, 71.7, 35.0; IR (NaCl, cm^{-1}) 1019, 1025, 1066, 1086, 1110, 1221, 1376, 1461, 1492, 3039; HRMS (ESI) *m/z*: $[M]^-$ Calcd for $C_9H_8ClOS_2^-$ 230.9705 Found 230.9701.

Potassium *O*-(pent-4-en-1-yl) carbonodithioate (**2h**)

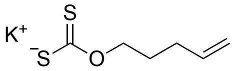

Yield: 82% (163 mg, 816 µmol); Colorless solid; Mp 200–201 ºC; ^{1}H NMR (DMSO-d_6, 400 MHz): δ 5.92–5.81 (m, 1H), 5.12–4.96 (m, 2H), 4.22 (t, 2H, J = 6.8 Hz), 2.14–2.07 (m, 2H), 1.75–1.66 (m, 2H); ^{13}C{^{1}H} NMR (DMSO-d_6, 101 MHz) δ 230.9, 139.3, 115.9, 70.9, 30.9, 28.7; IR (NaCl, cm^{-1}) 913, 1070, 1135, 1265, 1304, 1376, 1462, 1634; HRMS (ESI) *m/z*: $[M]^-$ Calcd for $C_6H_9OS_2^-$ 161.0095 Found 161.0097.

References for Supporting Information

S1 B. S. Shaibu, R. K. Kawade, R. S. Liu, *Org. Biomol. Chem*. **2012**, *10*, 6834.

S2 W. Bao, Z.-P. Gao, D.-P. Jin, C.-G. Xue, H. Liang, L.-S. Lei, X.-T. Xu, K. Zhang, S.-H. Wang, *Chem. Commun*. **2020**, *56*, 7641.

S3 C. Shen, G. Yang, W. Zhang, *Org. Lett*. **2013**, *15*, 5722.

S4 X. Li, Y. Sun, X. Huang, L. Zhang, L. Kong, B. Peng, *Org. Lett*. **2017**, *19*, 838.

S5 S. Yoshida, K. Shimomori, T. Nonaka, T. Hosoya, *Chem. Lett.* **2015**, *44*, 1324.

S6 Jose M. Medina, Joel L. Mackey, Neil K. Garg, K. N. Houk*, J. Am. Chem. Soc.* **2014**, *136*, 15798.

S7 S. Yoshida, Y. Nakamura, K. Uchida, Y. Hazama, T. Hosoya, *Org. Lett.* **2016***, 18*, 6212.

S8 Y. Nakamura, Y. Miyata, K. Uchida, S. Yoshida, T. Hosoya, *Org. Lett.* **2019***, 21,* 5252.

S9 F. I. Carroll, T. P. Robinson, L. E. Brieaddy, R. N. Atkinson, S. W. Mascarella, M. I. Damaj, B. R.Martin, H. A. Navarro, *J. Med. Chem.* **2007**, *50*, 6383.

S10 P. H.-Y. Cheong, R. S. Paton, S. M. Bronner, G-Y. J. Im, N. K. Garg, K. N. Houk, *J. Am. Chem. Soc*. **2010**, *132*, 1267.

S11 Ö. Güzel, A. Salman, *Bioorg. Med. Chem*. **2006**, *14*, 7804.

S12 C. Zhengwang, J. Huanfeng, L. Yibiao, Q. Chaorong, *Chem. Commun*. **2010**, *46*, 8049.

S13 Y. Gao, M. Yin, W. Wu, H. Huang, H. Jiang, *Adv. Synth. Catal.* **2013**, *355*, 2263.

S14 Y. Gao, G. Wu, Q. Zhou, J. Wang, *Angew. Chem., Int. Ed.* **2018**, *57*, 2716.

S15 A. Jančařík, J. Rybáček, K. Cocq, J. V. Chocholoušová, J. Vacek, R. Pohl, L. Bednárová, P. Fiedler, I. Císařová, I. G. Stará, I. Starý, *Angew. Chem., Int. Ed.* **2013**, *52,* 9970.

S16 D. I. Bugaenko, A. A. Volkov, V. V. Andreychev, A. V. Karchava, *Org. Lett.* **2023**, *25*, 272.

S17 G. Wang, J. Jia, Y. He, D. Wei, M. Song, L. Zhang, G. Li, H. Li, B. Yuan, *RSC Adv*. **2022**, *12*, 18407.

S18 B. Zhang, Z. Fan, Z. Guo, C. Xi, *J. Org. Chem*. **2019**, *84*, 8661.

S19 S. Madabhushi, R. Jillella, V. Sriramojua, R. Singh, *Green Chem*. **2014**, *16*, 3125.

S20 Y. Liu, D. Yu, Y. Guo, J.-C. Xiao, Q.-Y. Chen, C. Liu, *Org. Lett*. **2020**, *22*, 2281.

^{1}H and ^{13}C NMR Spectra of New Compounds

^{1}H NMR (400 MHz) and ^{13}C NMR (101 MHz) spectra of *S*-(3,4-dimethoxyphenyl) *O*-ethyl carbonodithioate (**3a**) ($CDCl_3$)

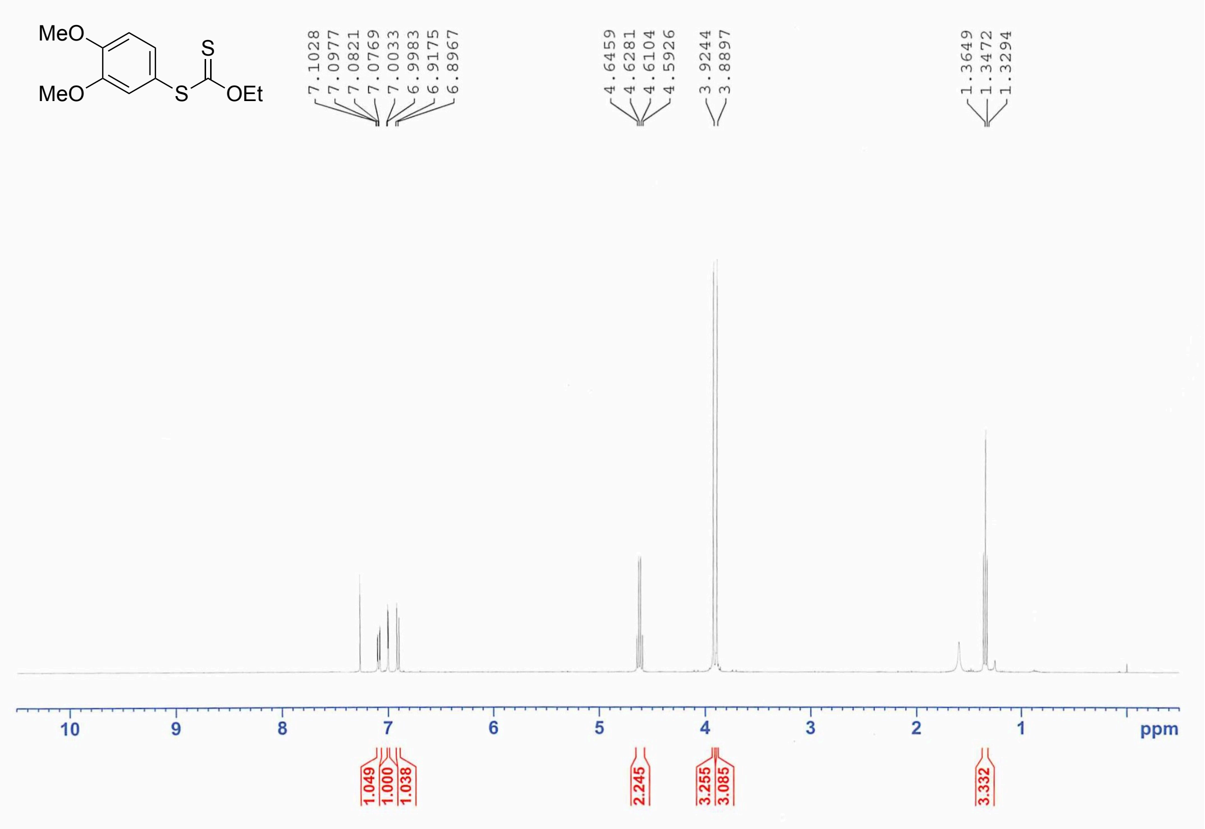

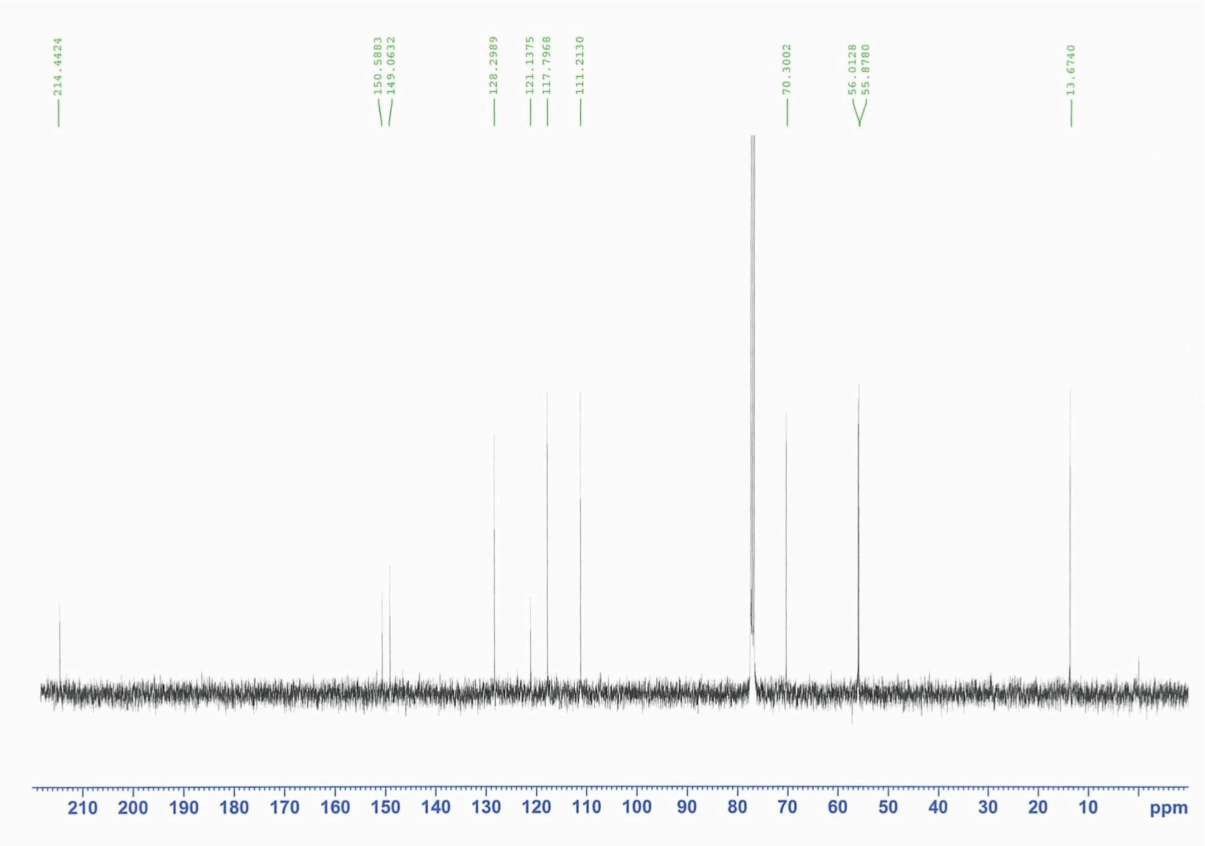

^{1}H NMR (400 MHz) and ^{13}C NMR (101 MHz) spectra of *S*-(benzo[*d*][1,3]dioxol-5-yl) *O*-ethyl carbonodithioate (**3c**) ($CDCl_3$)

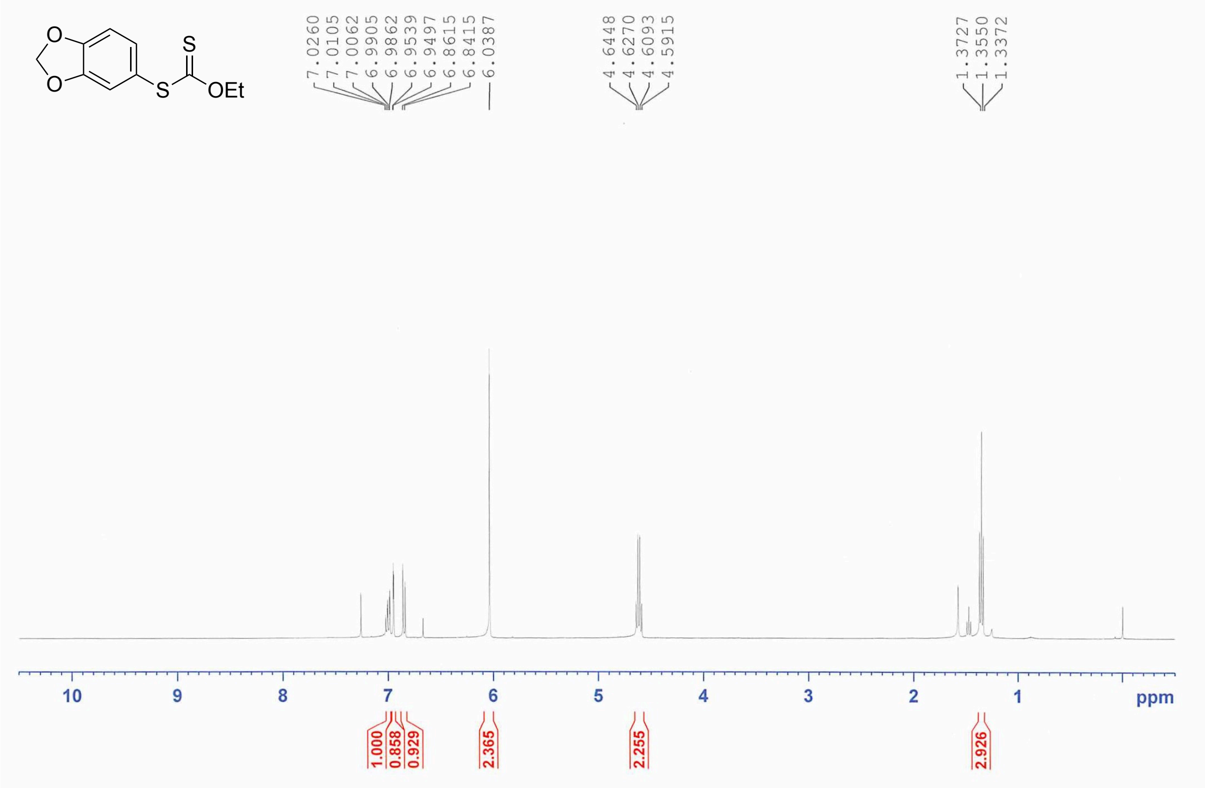

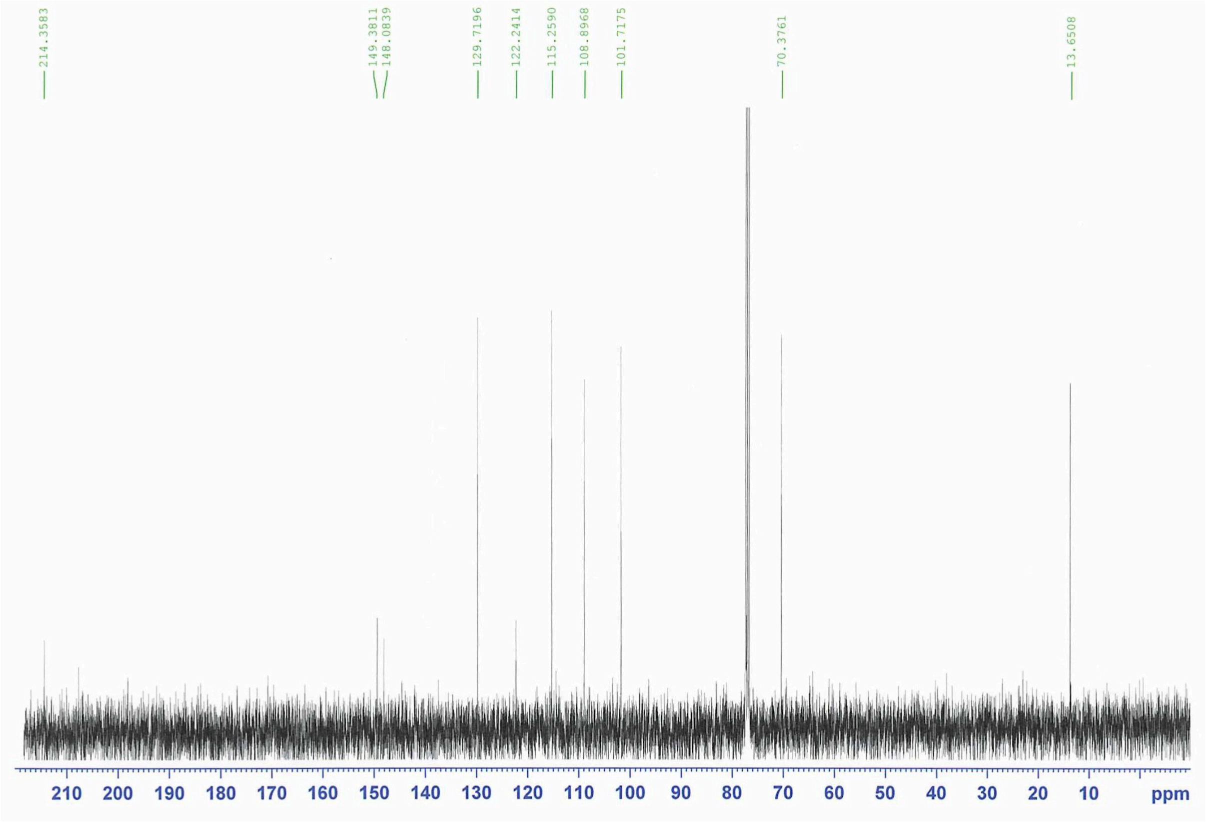

^{1}H NMR (400 MHz) and ^{13}C NMR (101 MHz) spectra of *S*-(3,4-difluorophenyl) *O*-ethyl carbonodithioate (**3d**) ($CDCl_3$)

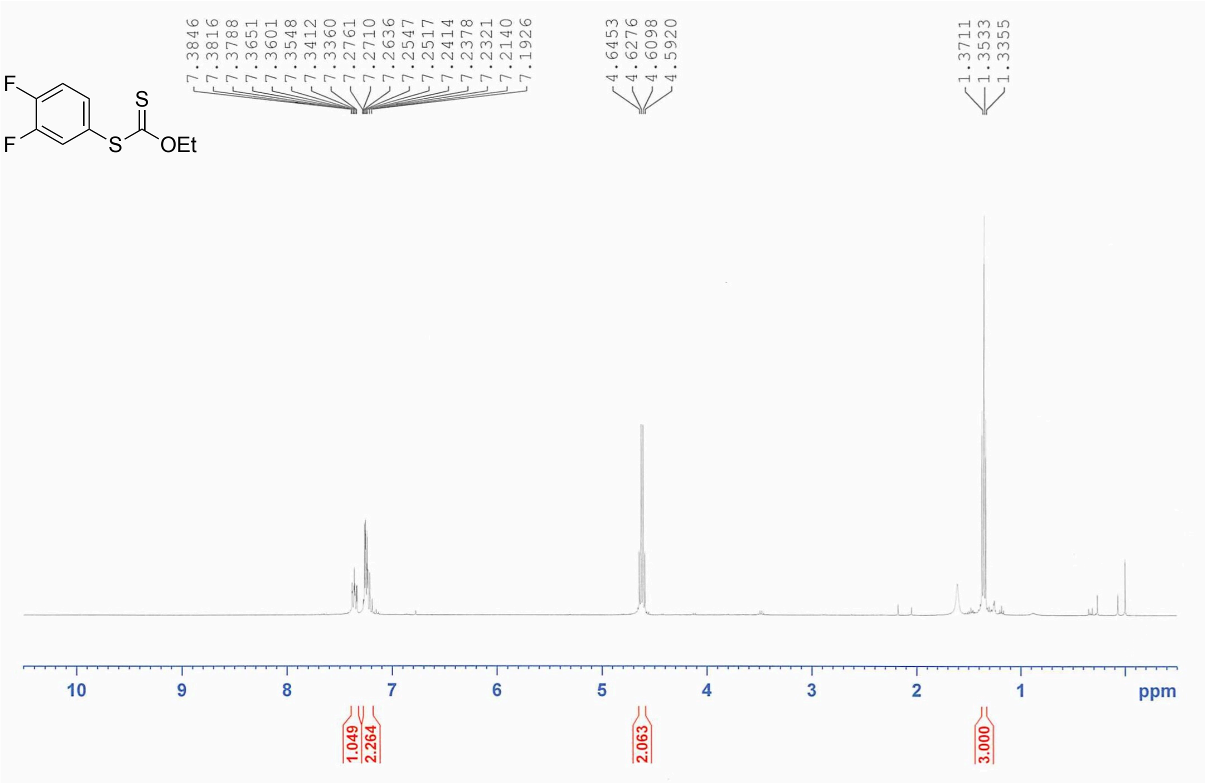

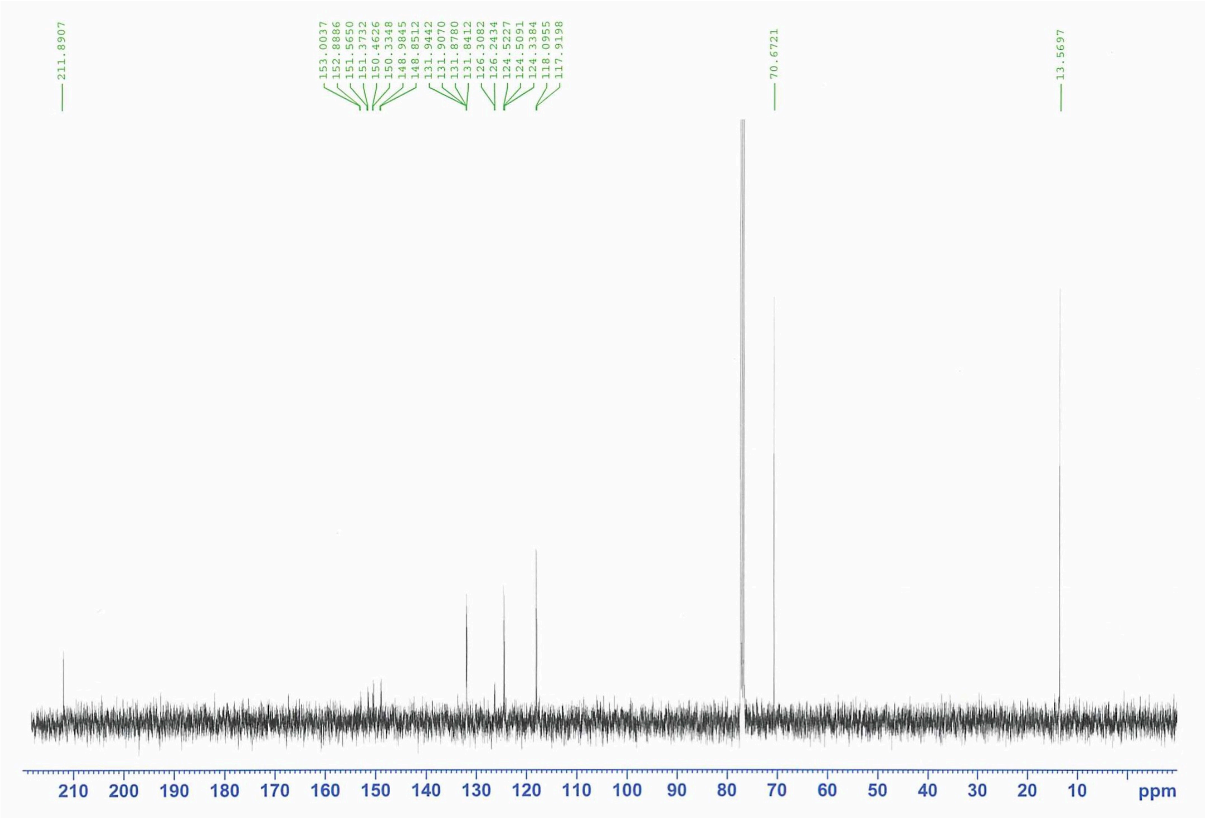

^{1}H NMR (400 MHz) and ^{13}C NMR (101 MHz) spectra of *S*-(3-azido-5-methoxyphenyl) *O*-ethyl carbonodithioate (**3f**) ($CDCl_3$)

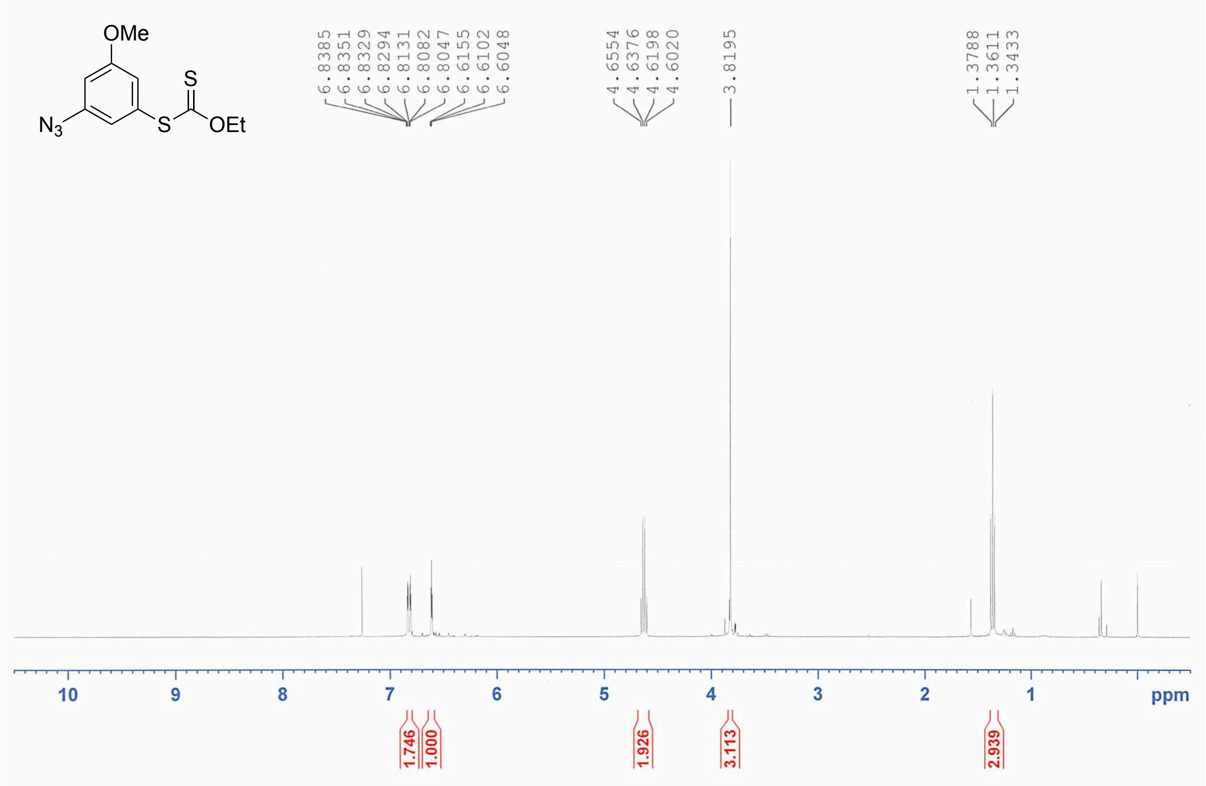

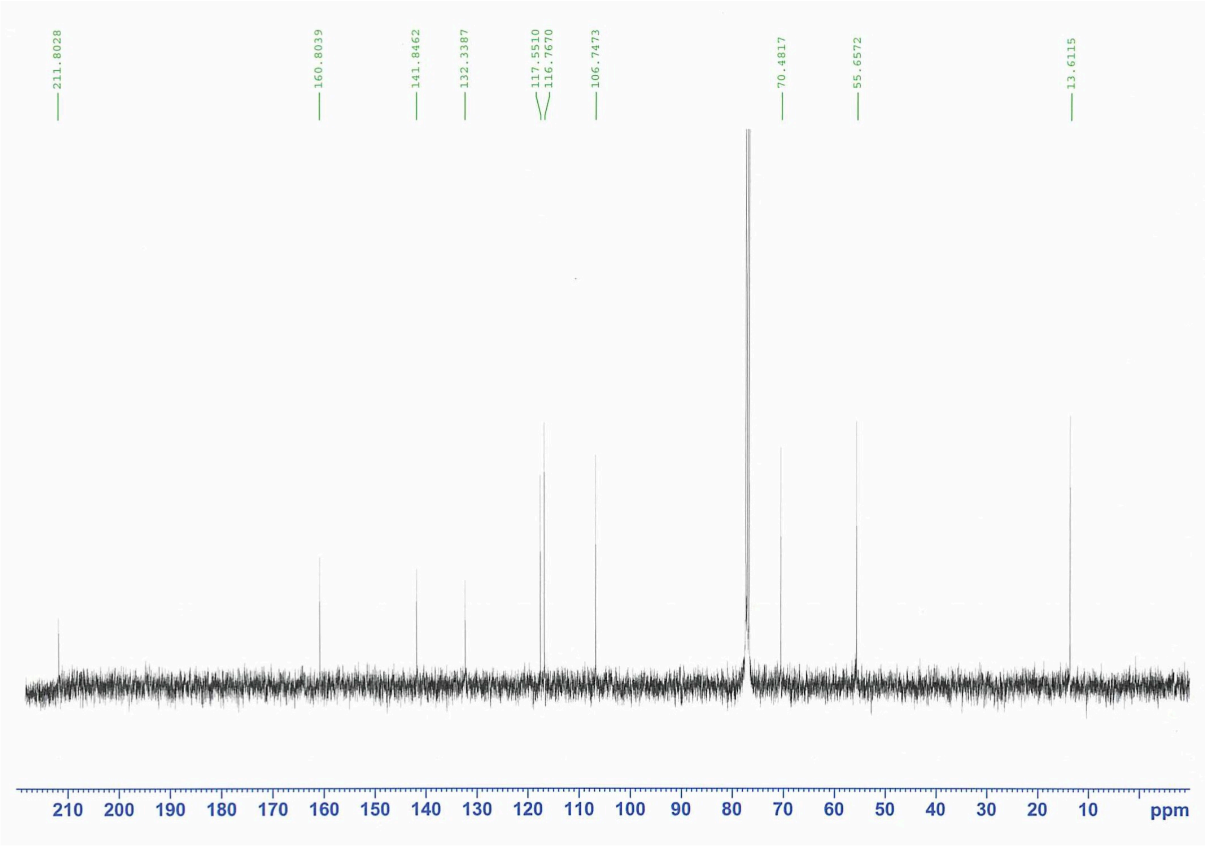

^{1}H NMR (400 MHz) and ^{13}C NMR (101 MHz) spectra of *S*-(3-bromophenyl) *O*-ethyl carbonodithioate (**3g**) ($CDCl_3$)

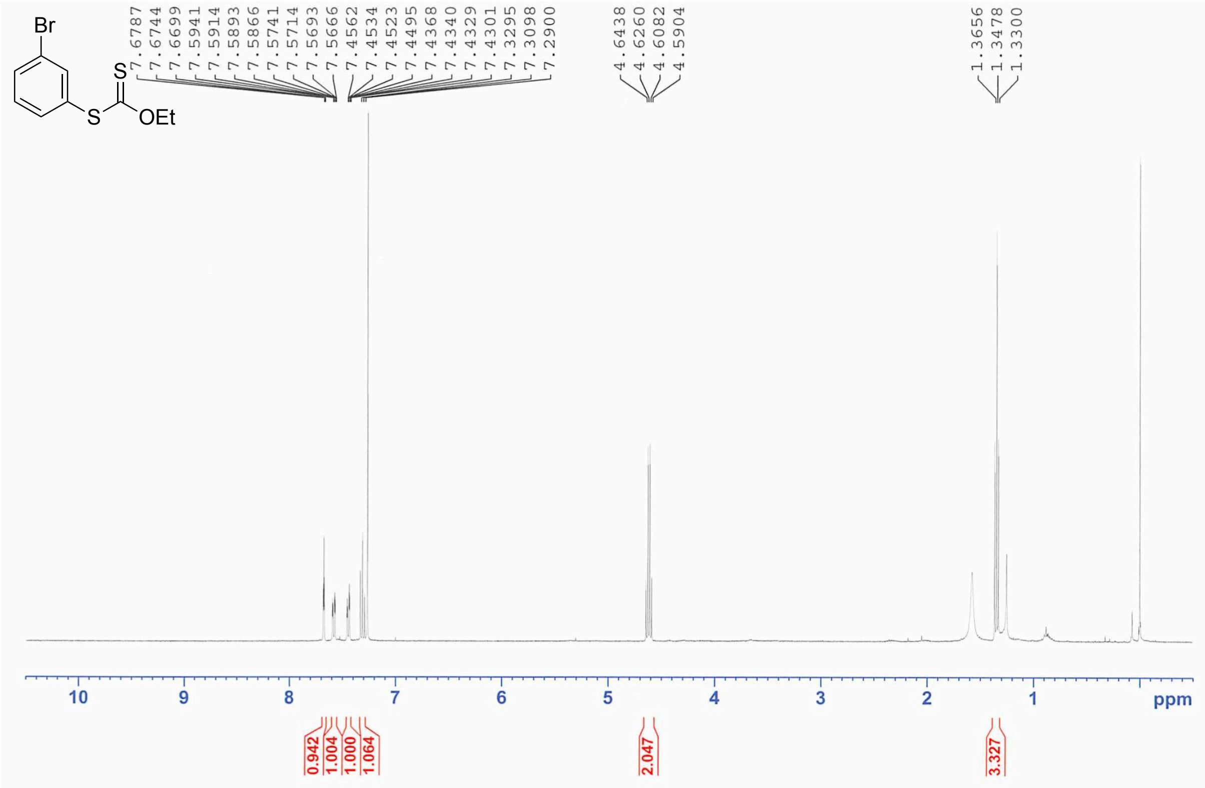

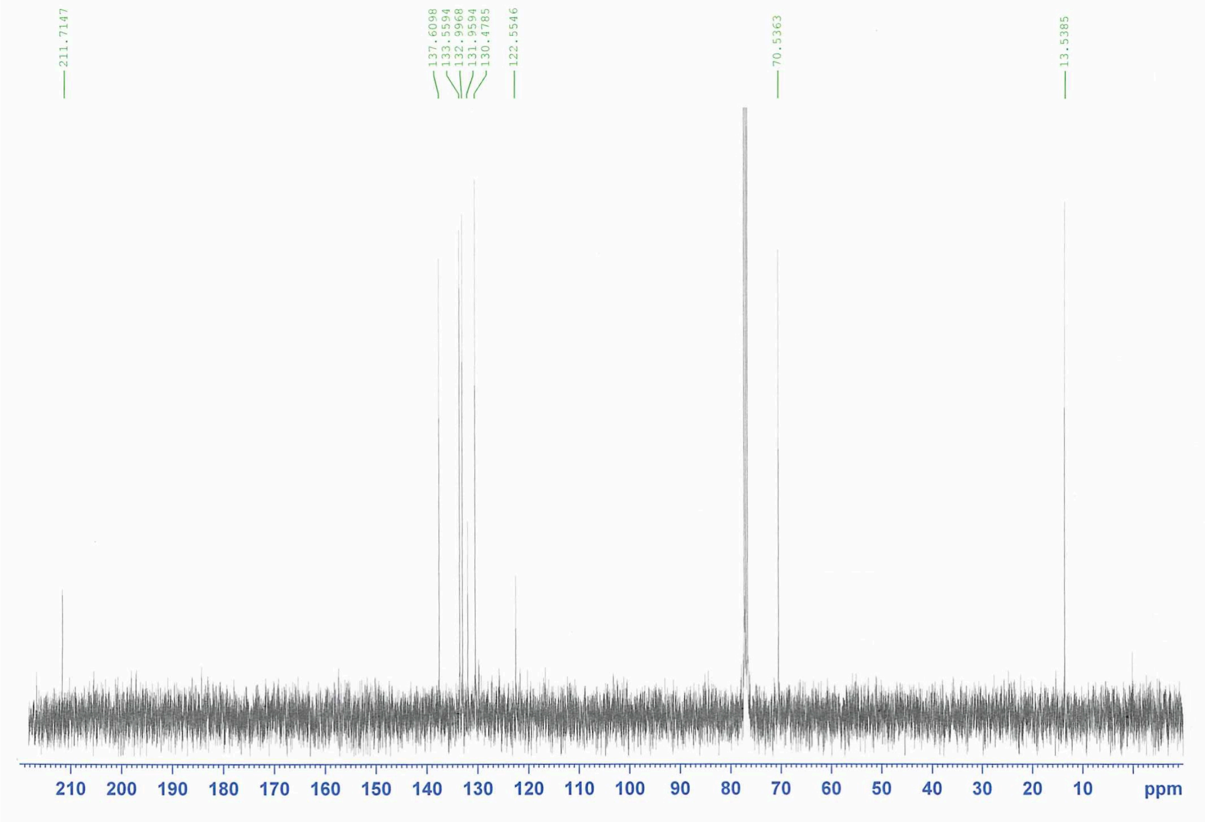

^{1}H NMR (400 MHz) and ^{13}C NMR (101 MHz) spectra of *S*-(3-chlorophenyl) *O*-ethyl carbonodithioate (**3h**) ($CDCl_3$)

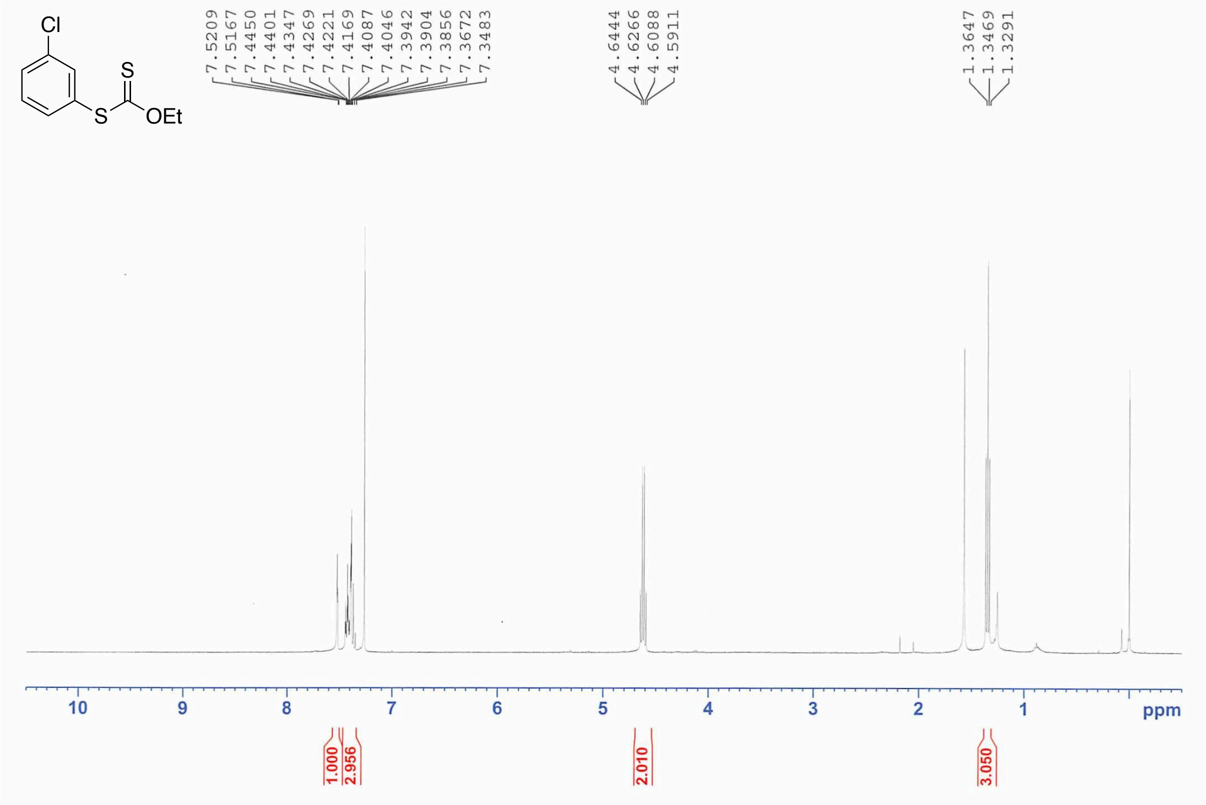

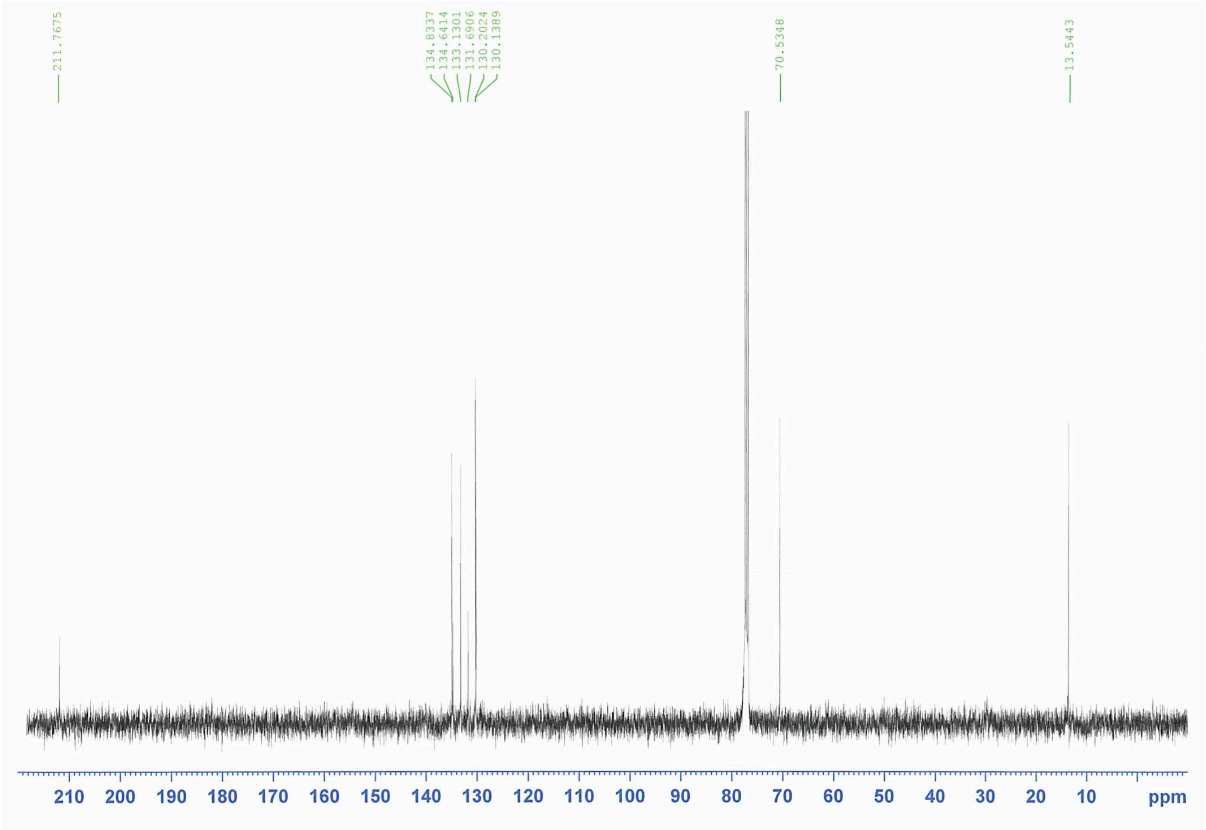

^{1}H NMR (400 MHz) and ^{13}C NMR (101 MHz) spectra of *O*-ethyl *S*-(3-morpholinophenyl) carbonodithioate (**3i**) ($CDCl_3$)

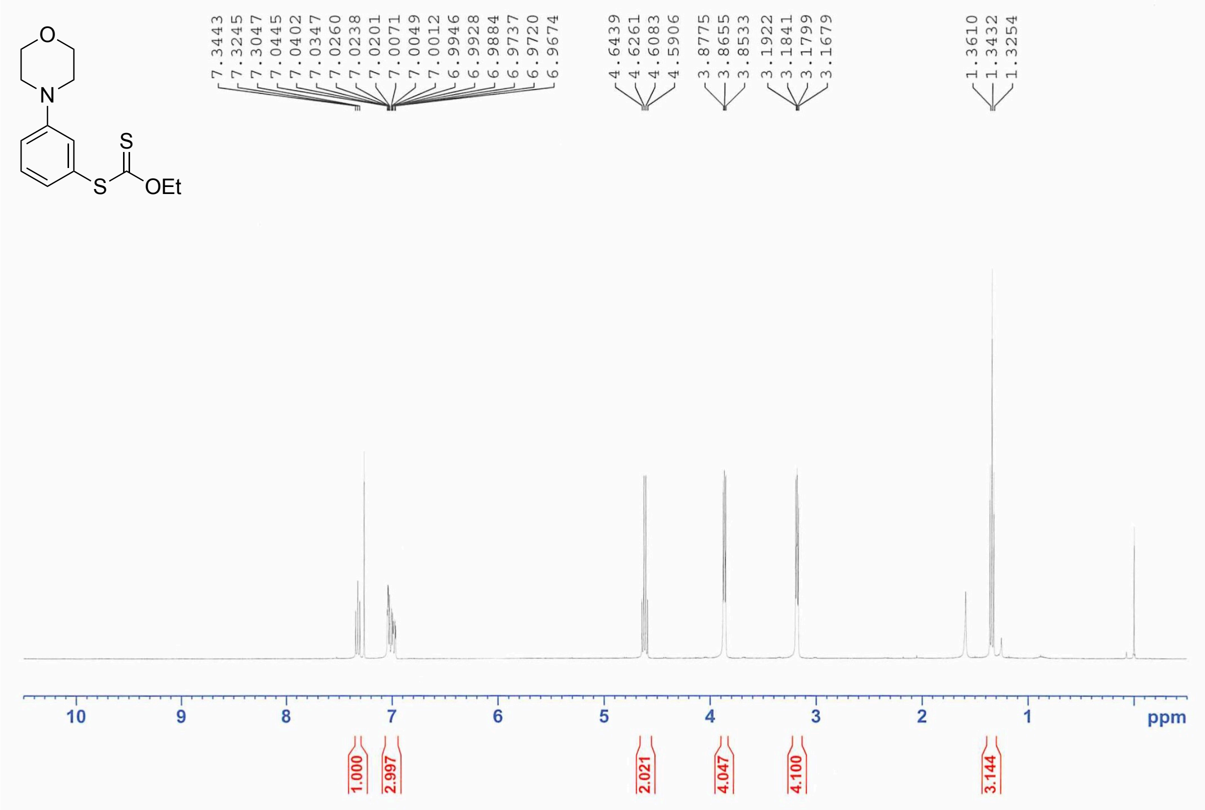

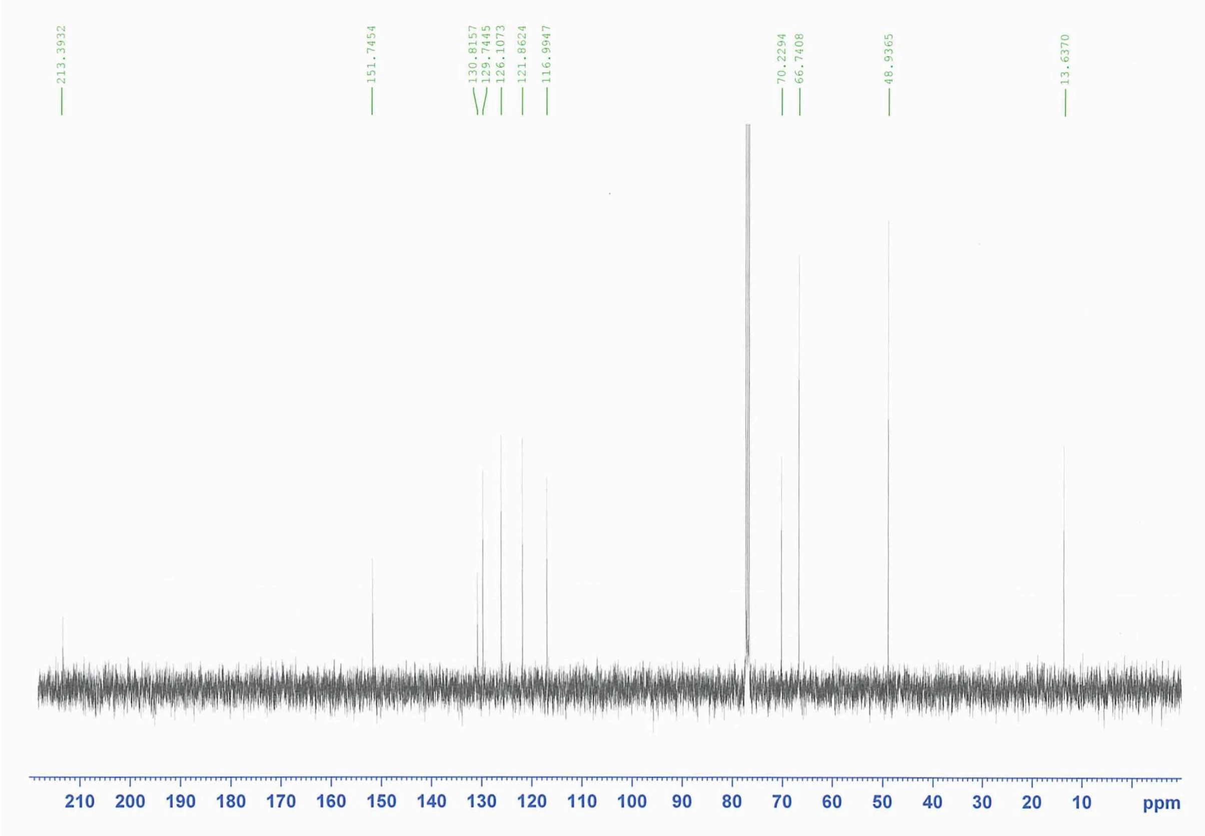

^{1}H NMR (400 MHz) and ^{13}C NMR (101 MHz) spectra of *O*-ethyl *S*-(3-((4-methoxyphenyl)thio)phenyl) carbonodithioate (**3j**) ($CDCl_3$)

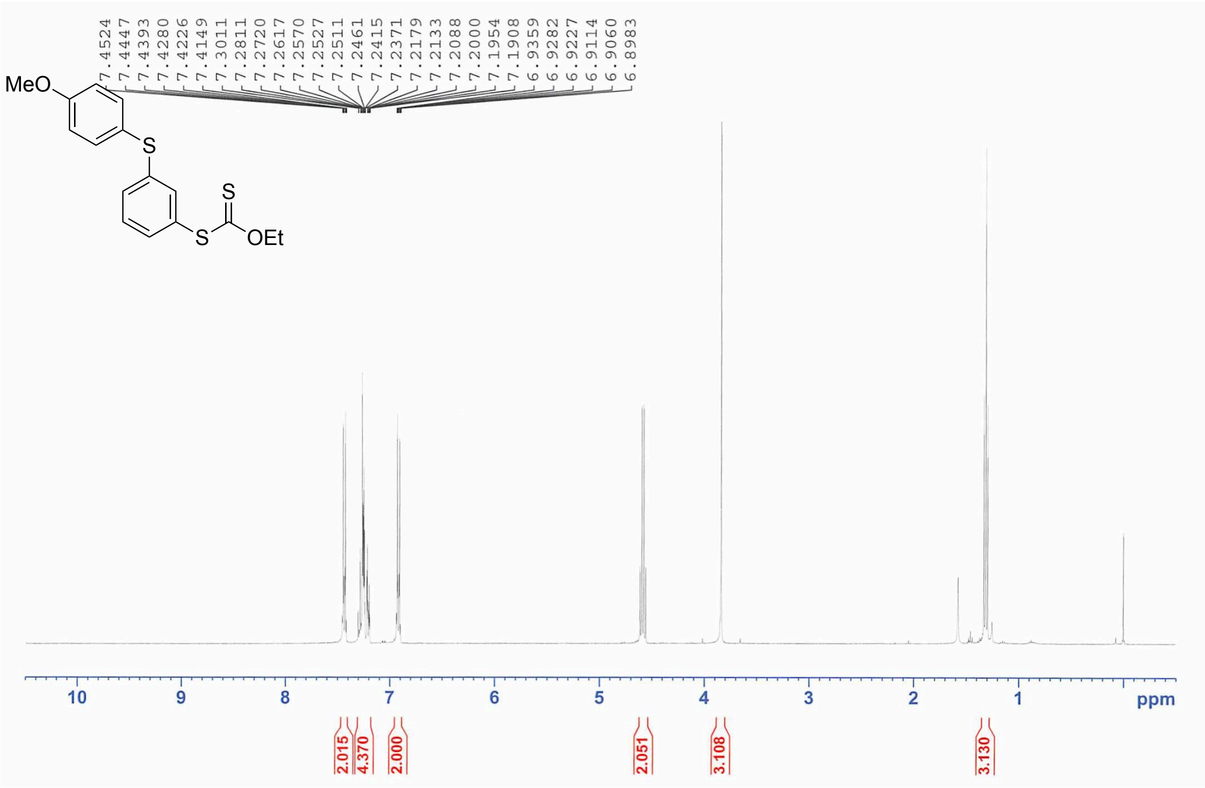

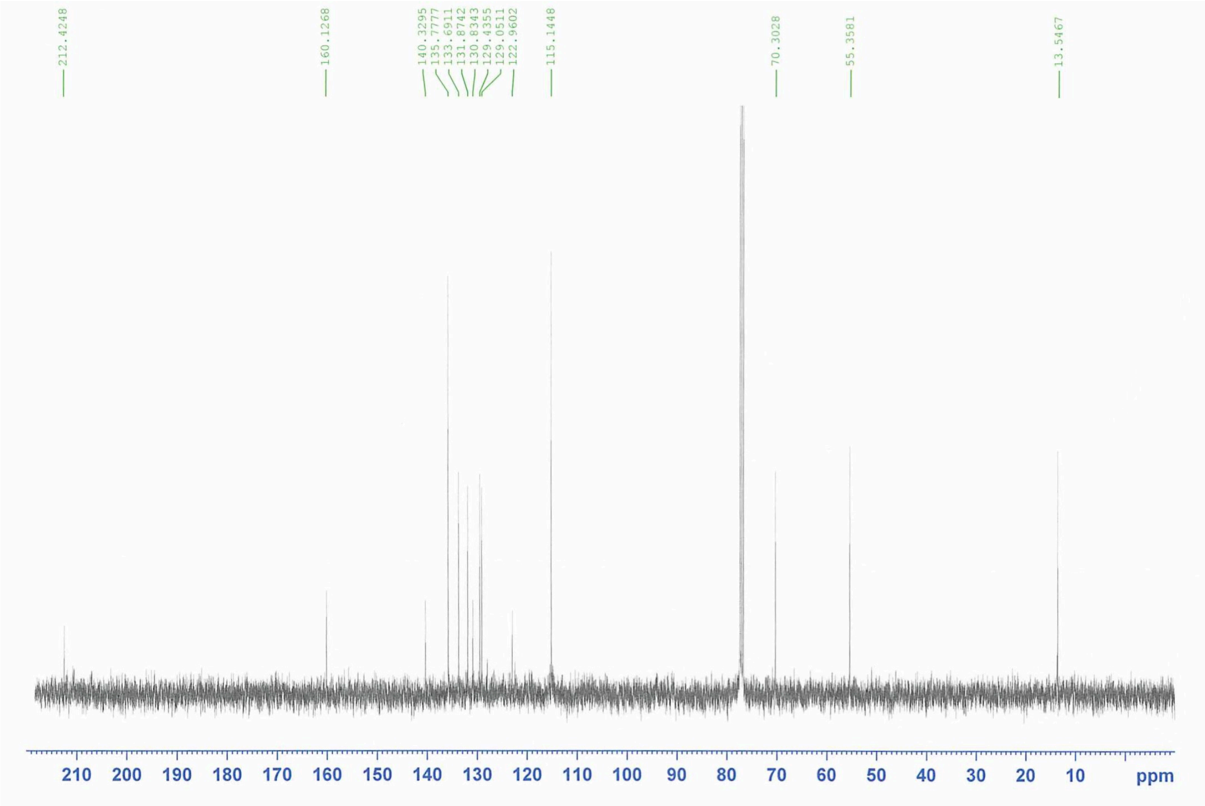

^{1}H NMR (400 MHz) and ^{13}C NMR (101 MHz) spectra of *O*-ethyl *S*-(naphthalen-2-yl) carbonodithioate (**3k**) ($CDCl_3$)

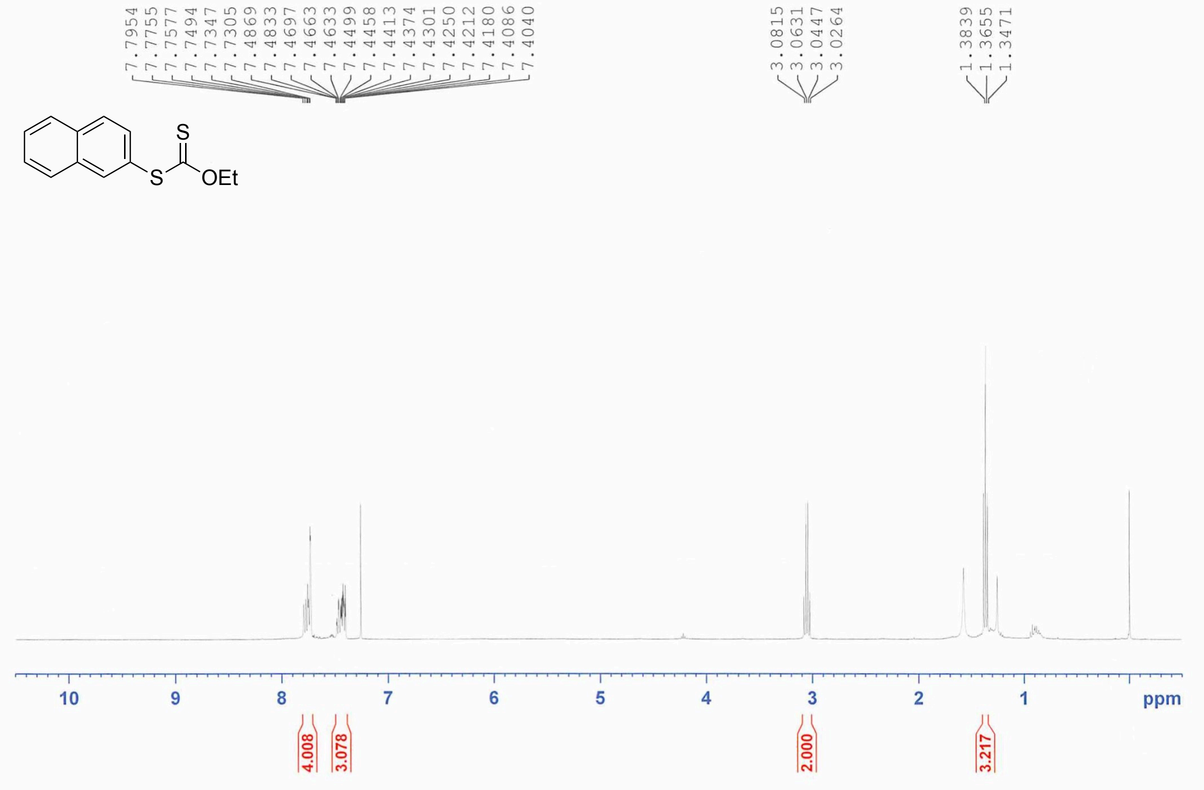

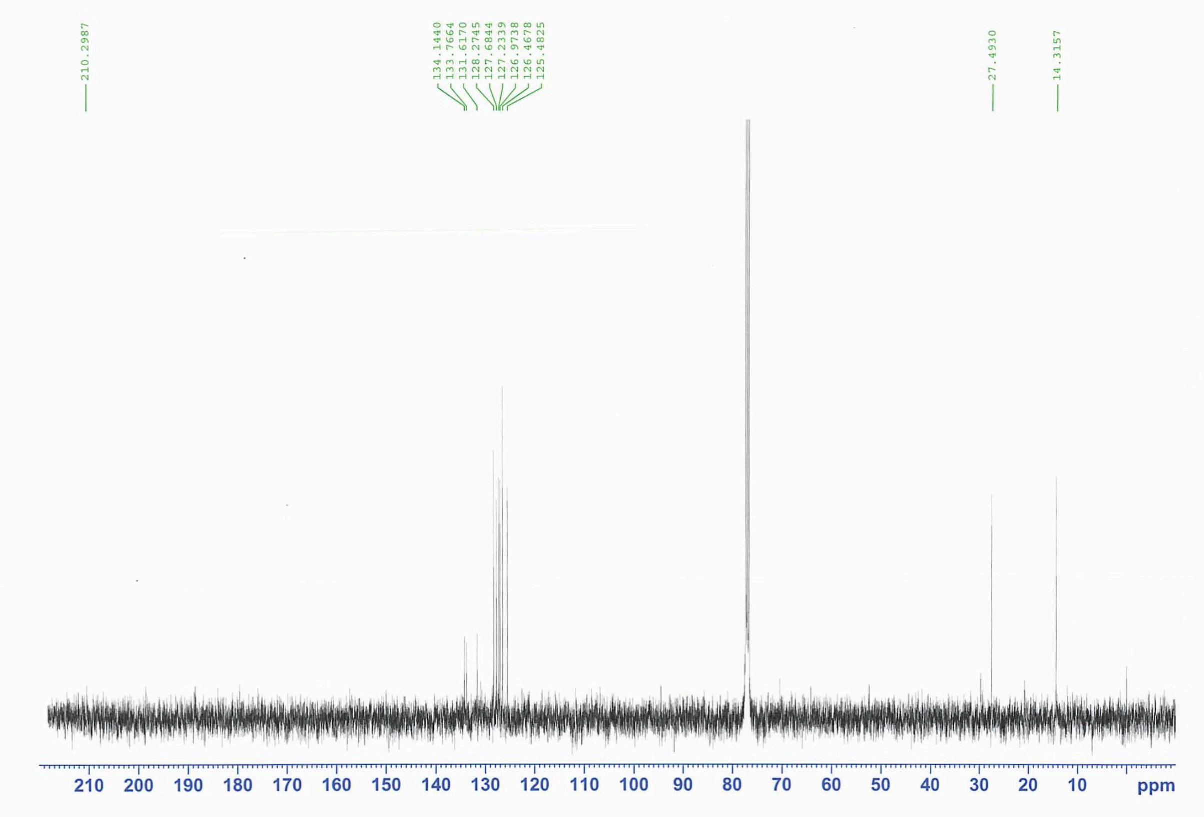

^{1}H NMR (400 MHz) and ^{13}C NMR (101 MHz) spectra of *O*-ethyl *S*-(pyridin-2-yl) carbonodithioate (**3l**) ($CDCl_3$)

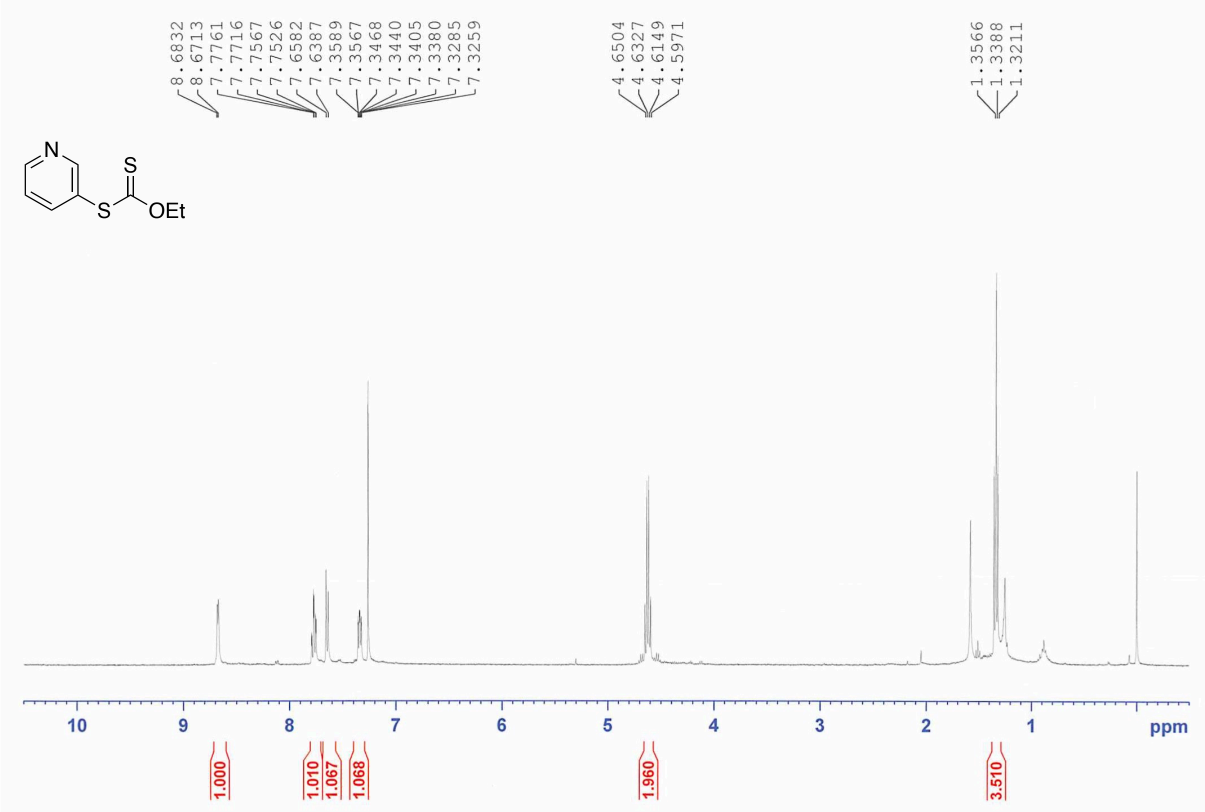

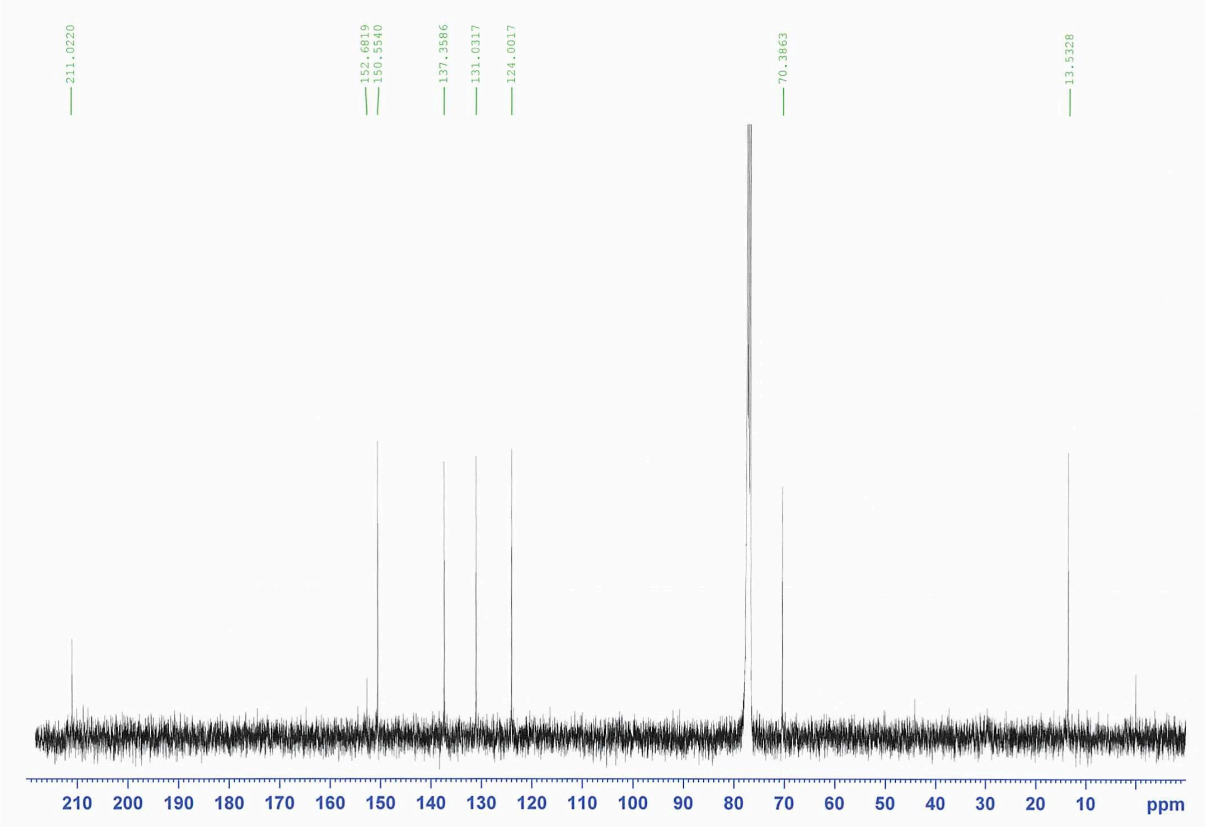

^{1}H NMR (400 MHz) and ^{13}C NMR (101 MHz) spectra of *O*-ethyl *S*-(1-methyl-1*H*-indol-6-yl) carbonodithioate (**3m**) ($CDCl_3$)

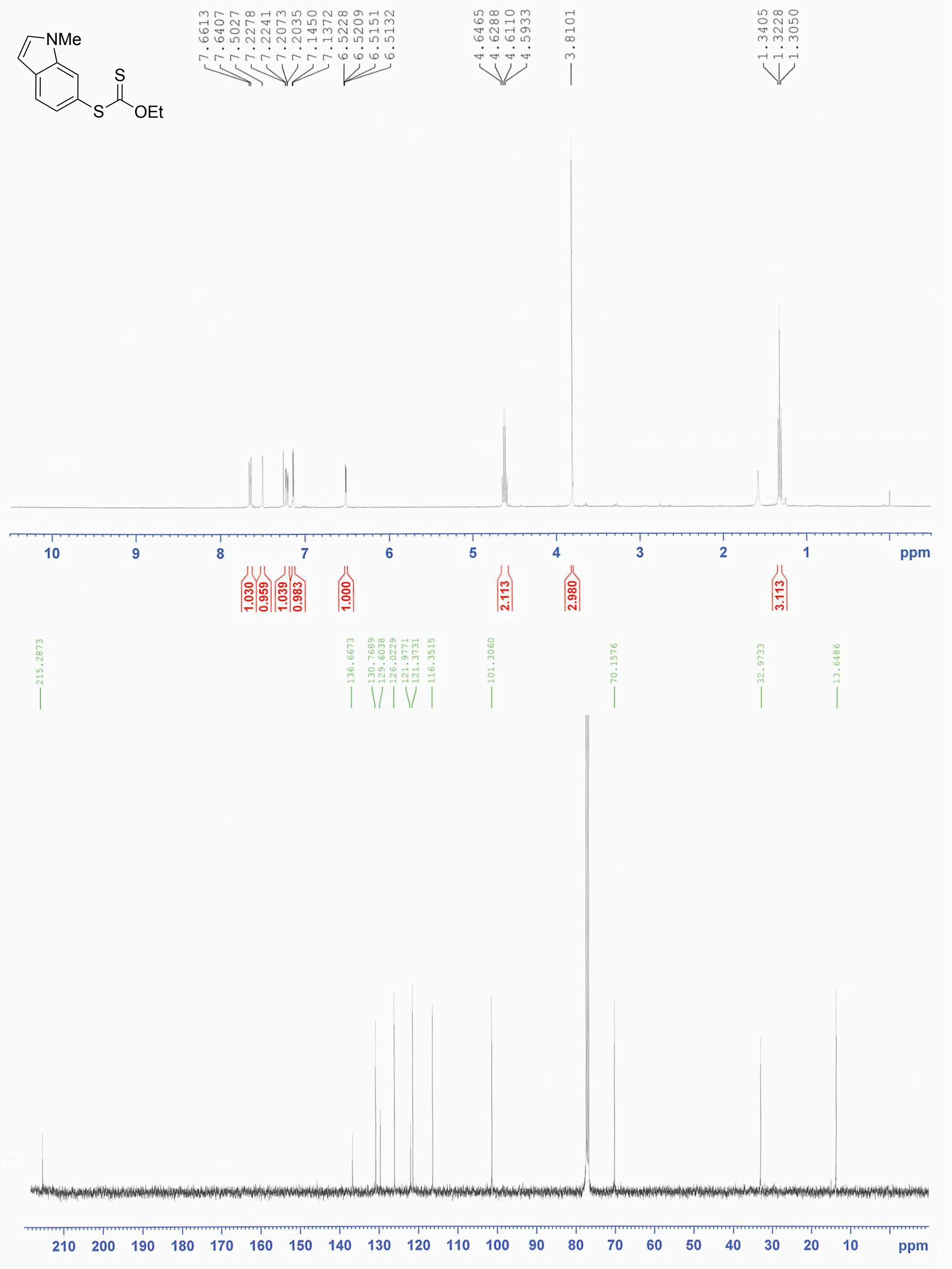

^{1}H NMR (400 MHz) and ^{13}C NMR (101 MHz) spectra of *S*-(2-bromo-4,5-dimethoxyphenyl) *O*-ethyl carbonodithioate (**8a**) ($CDCl_3$)

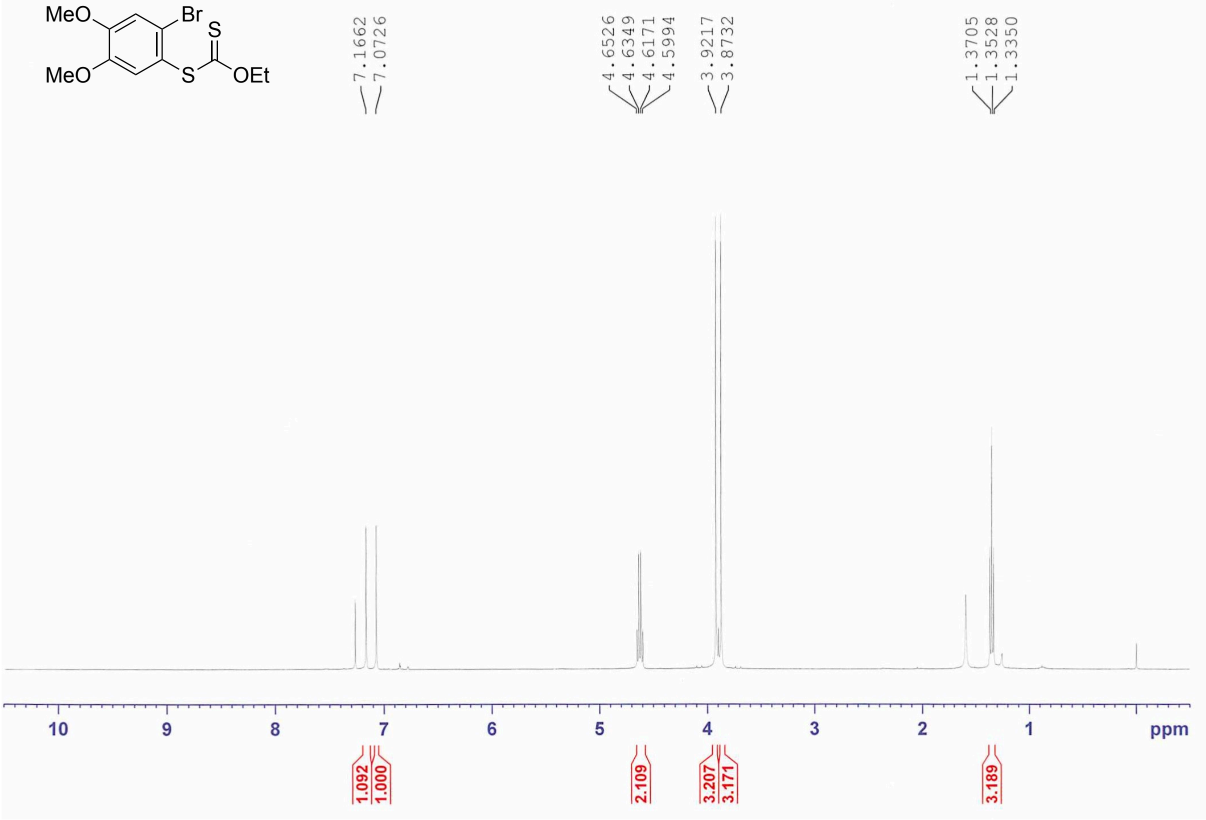

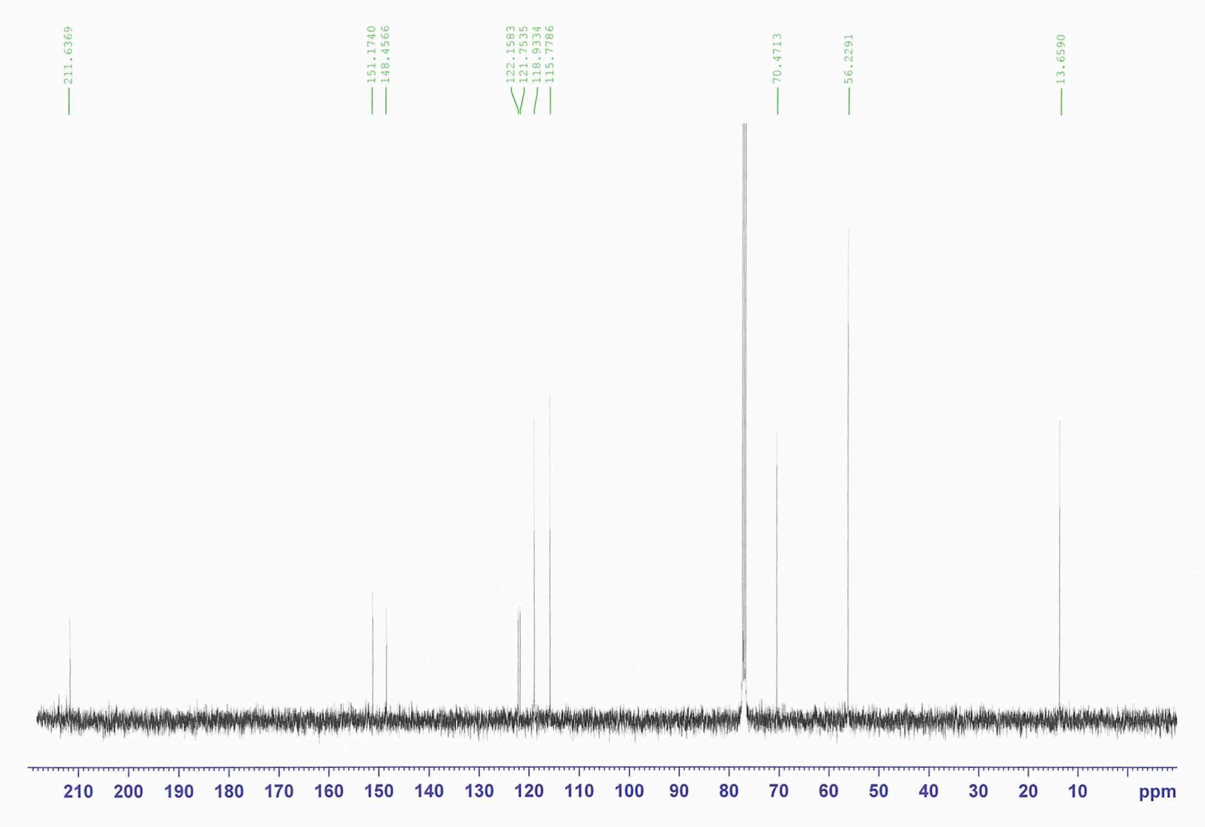

^{1}H NMR (400 MHz) and ^{13}C NMR (101 MHz) spectra of *S*-(6-bromobenzo[*d*][1,3]dioxol-5-yl) *O*-ethyl carbonodithioate (**8c**) ($CDCl_3$)

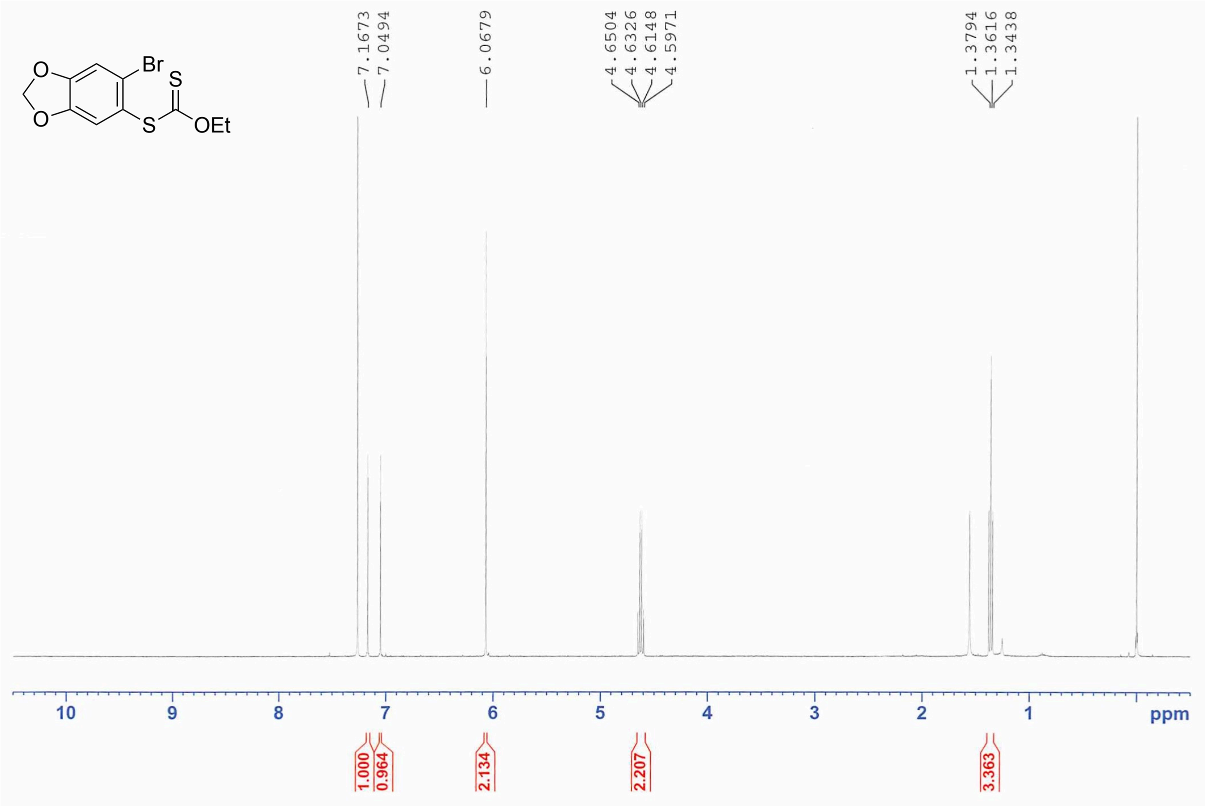

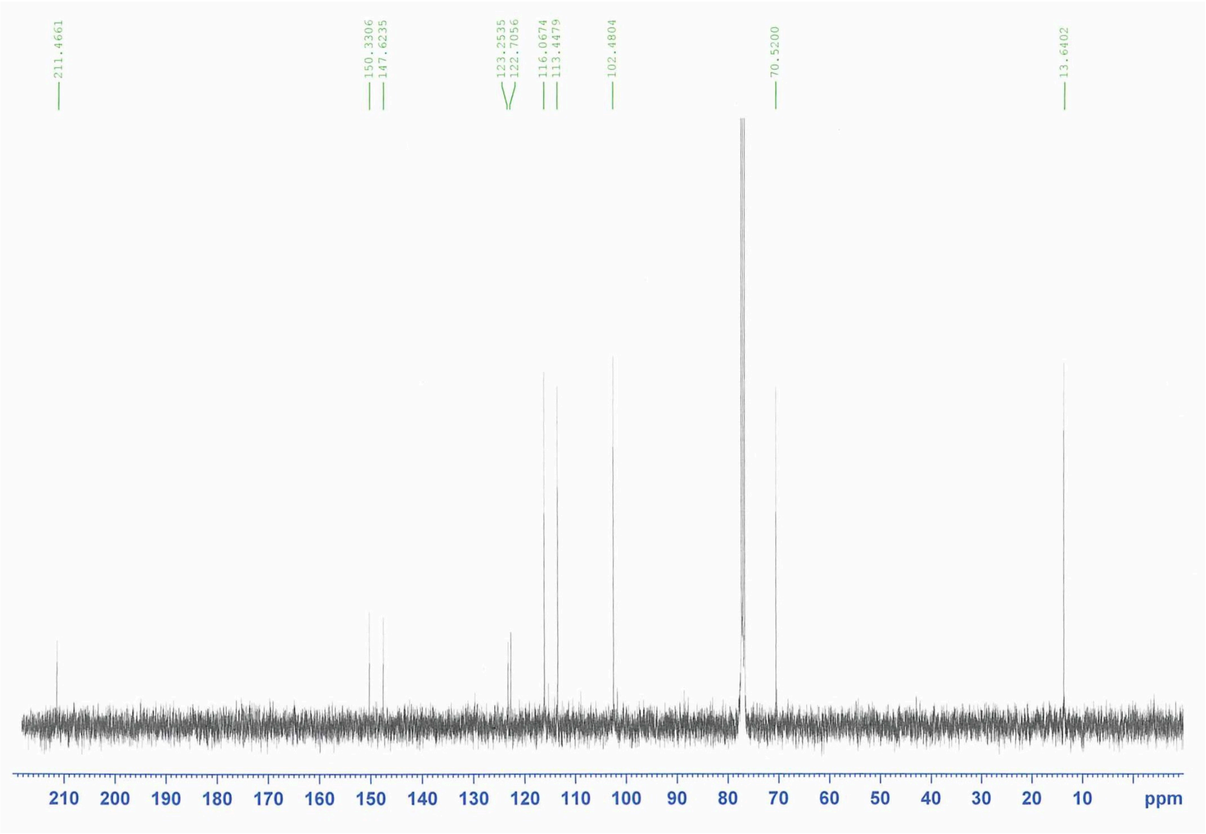

^{1}H NMR (400 MHz) and ^{13}C NMR (101 MHz) spectra of *S*-(3-bromonaphthalen-2-yl) *O*-ethyl carbonodithioate (**8d**) ($CDCl_3$)

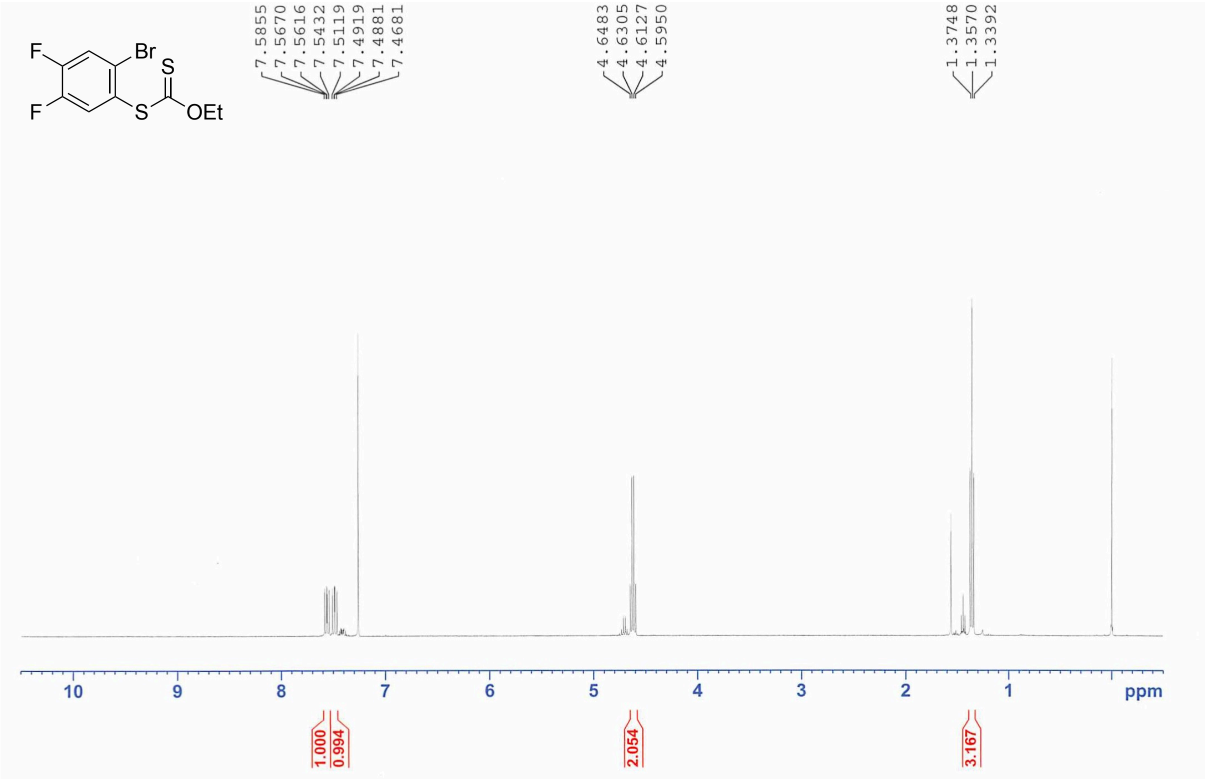

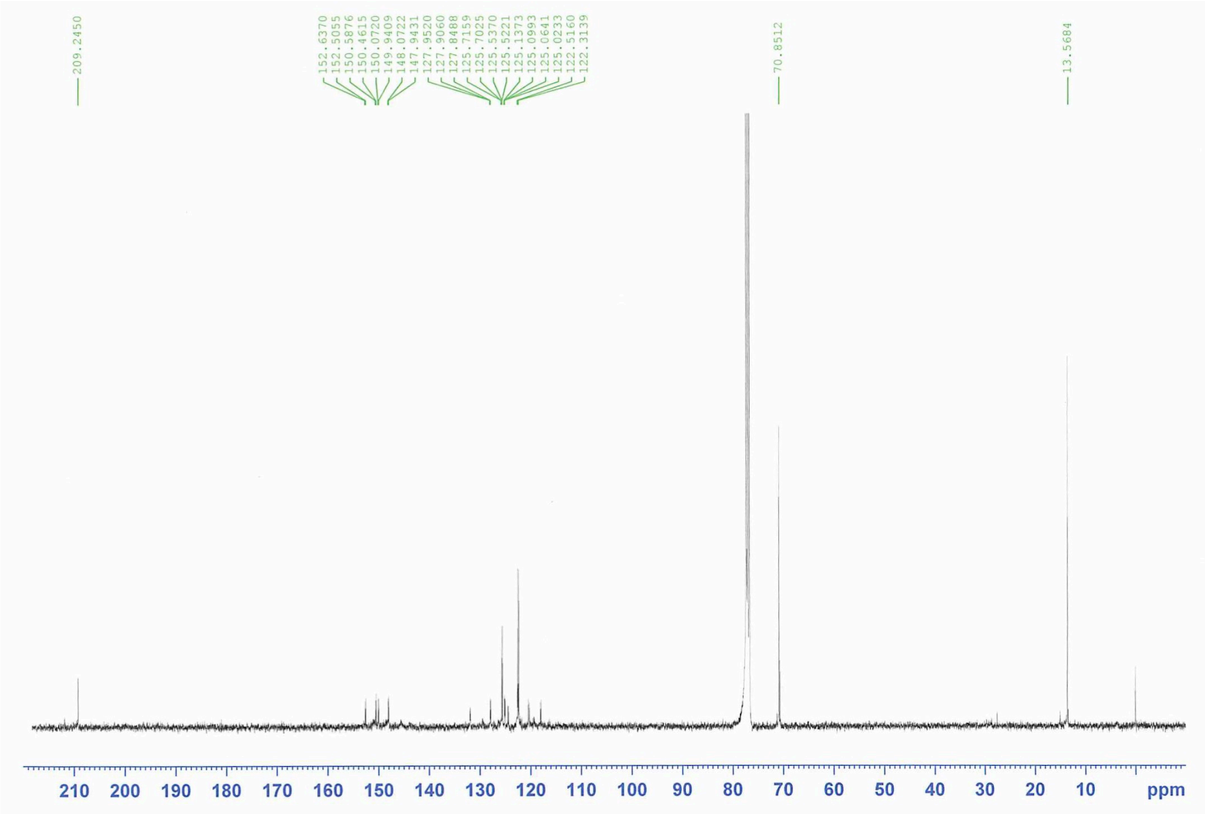

^{1}H NMR (400 MHz) and ^{13}C NMR (101 MHz) spectra of *S*-(2-bromo-3-methoxyphenyl) *O*-ethyl carbonodithioate (**8e**) ($CDCl_3$)

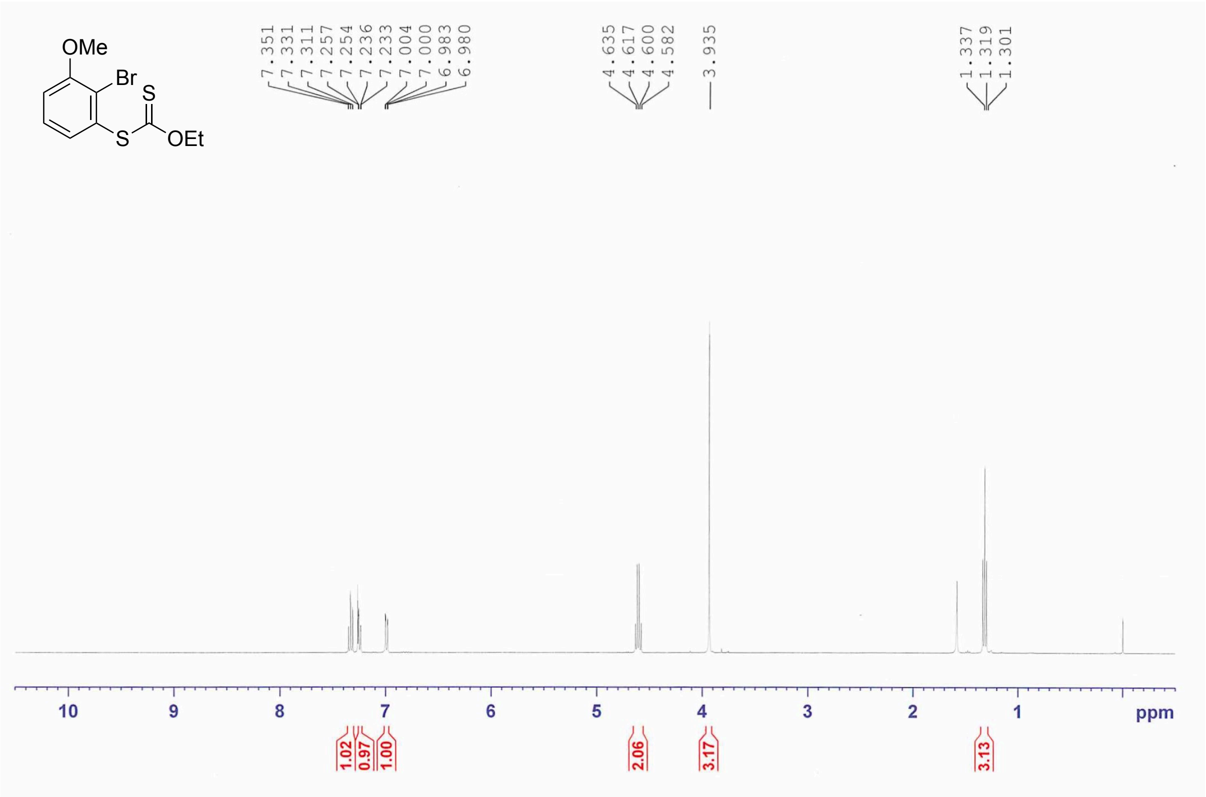

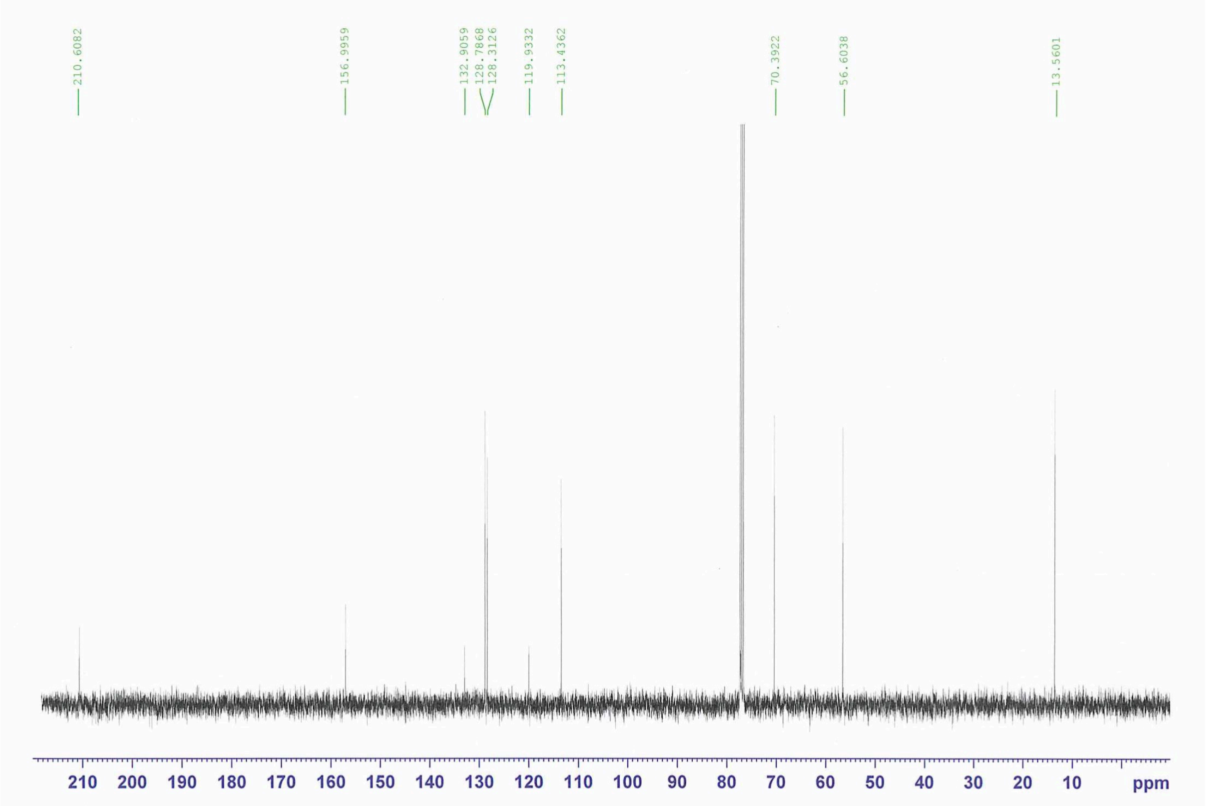

^{1}H NMR (400 MHz) and ^{13}C NMR (101 MHz) spectra of *S*-(5-azido-2-bromo-3-methoxyphenyl) *O*-ethyl carbonodithioate (**8f**) ($CDCl_3$)

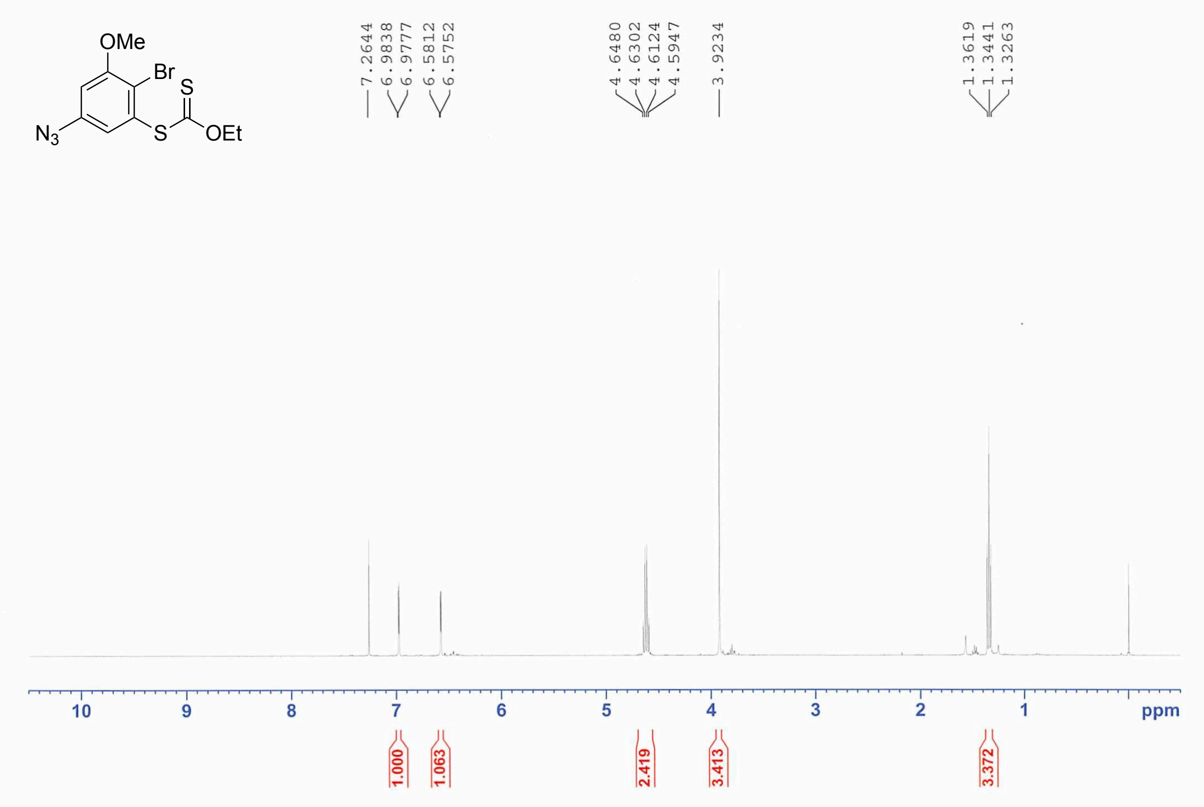

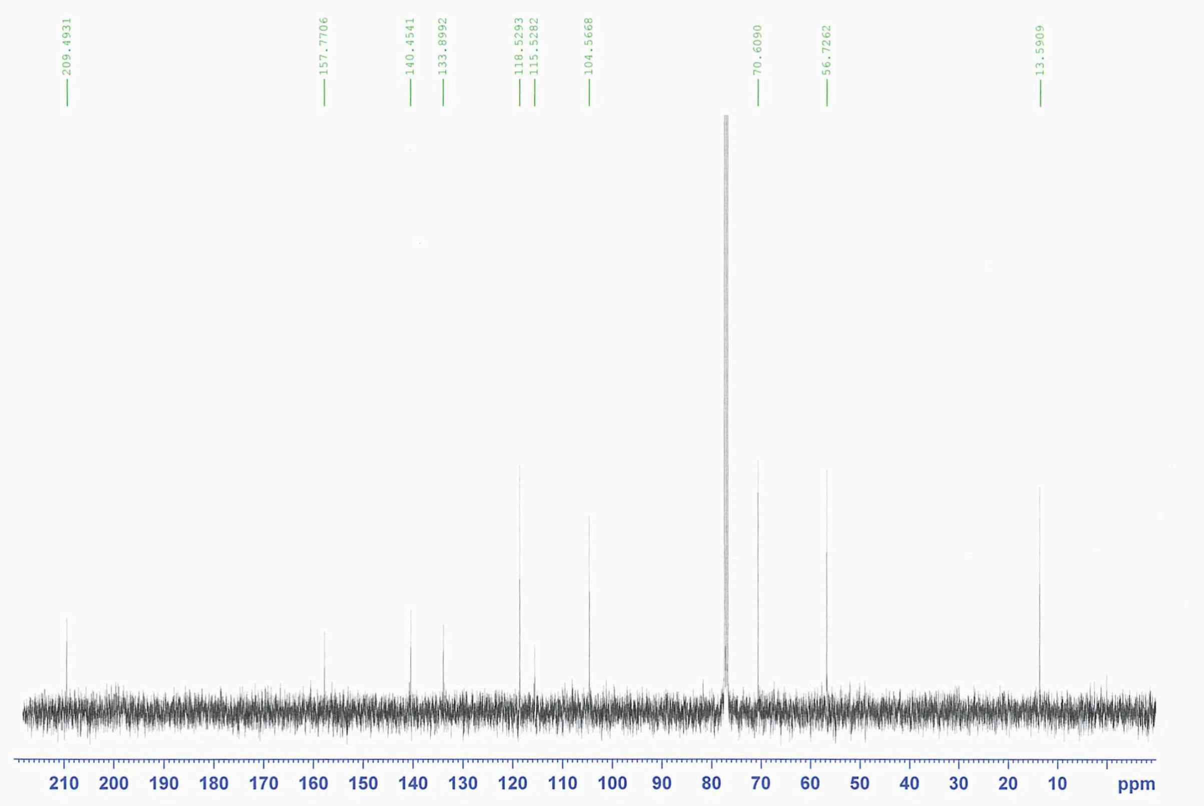

^{1}H NMR (400 MHz) and ^{13}C NMR (101 MHz) spectra of *S*-(2,3-dibromophenyl) *O*-ethyl carbonodithioate (**8g**) ($CDCl_3$)

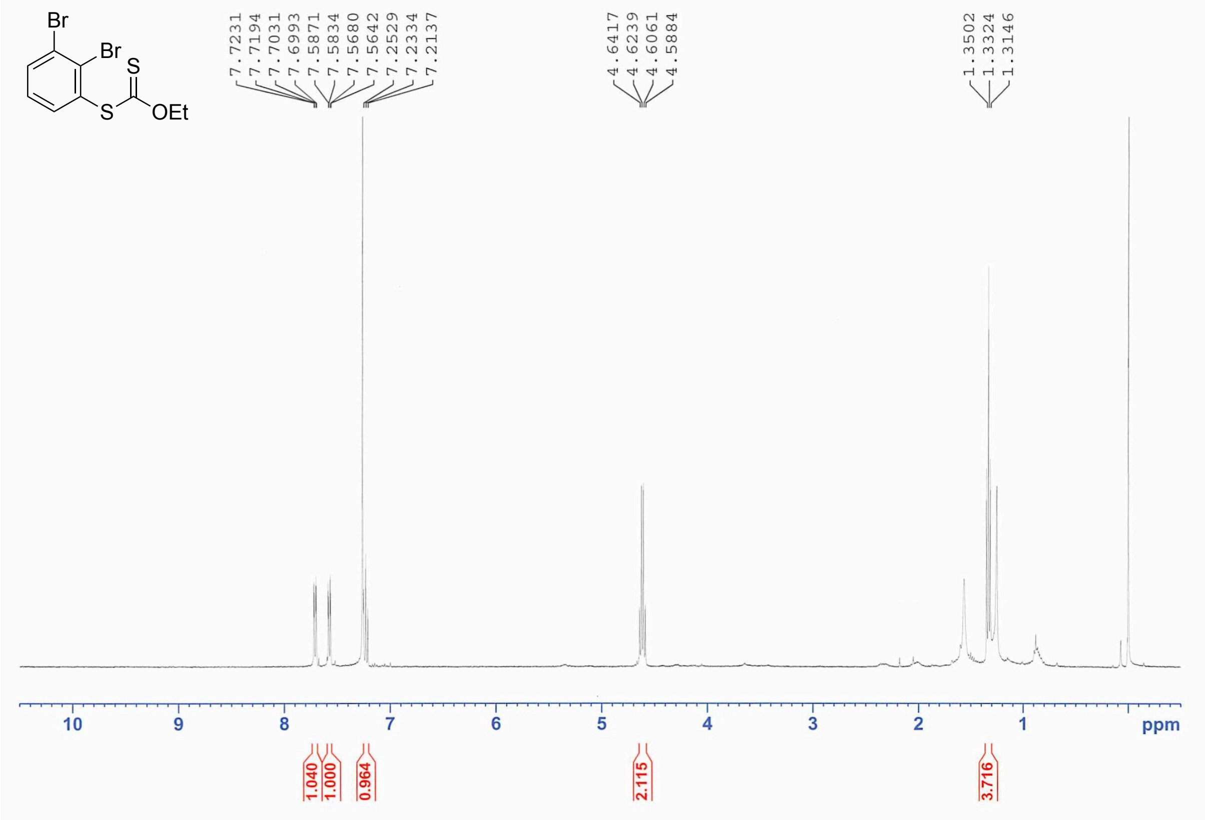

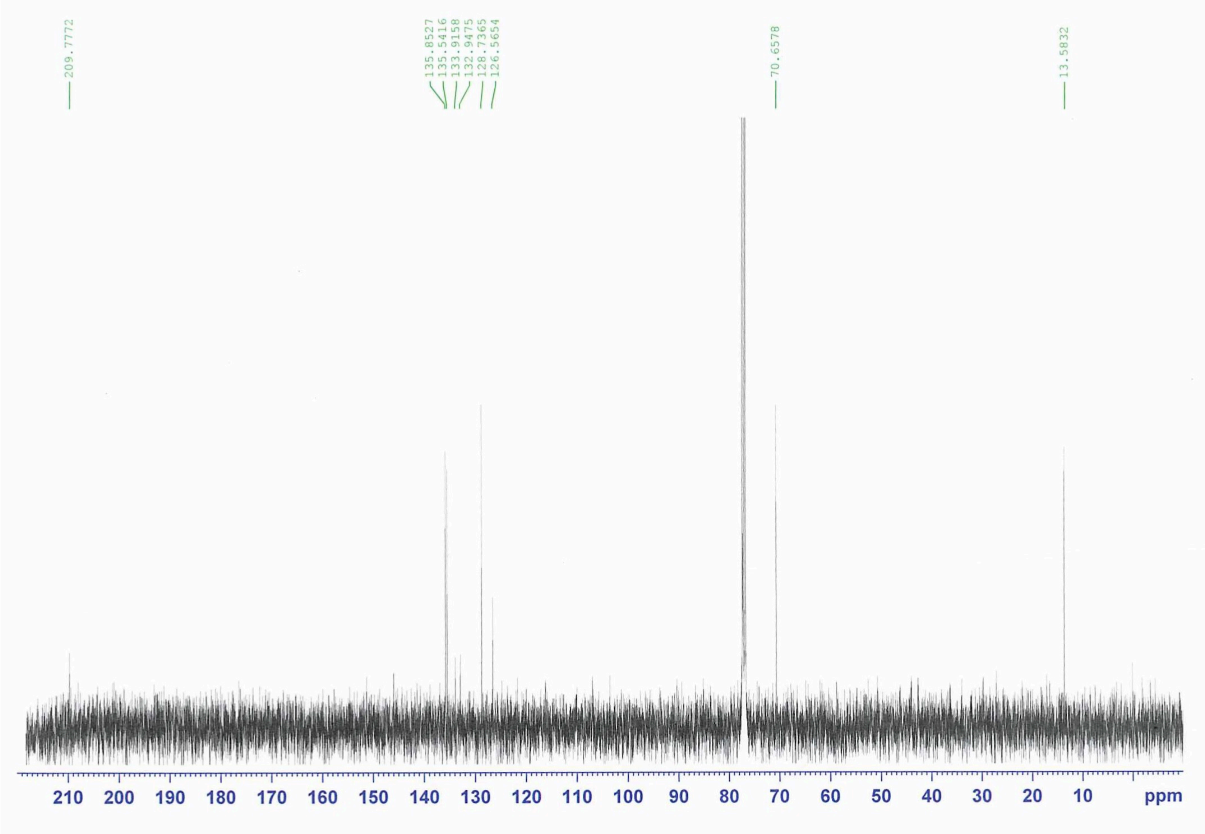

^{1}H NMR (400 MHz) and ^{13}C NMR (101 MHz) spectra of *S*-(2-bromo-3-((4-methoxyphenyl)thio)phenyl) *O*-ethyl carbonodithioate (**8h**) ($CDCl_3$)

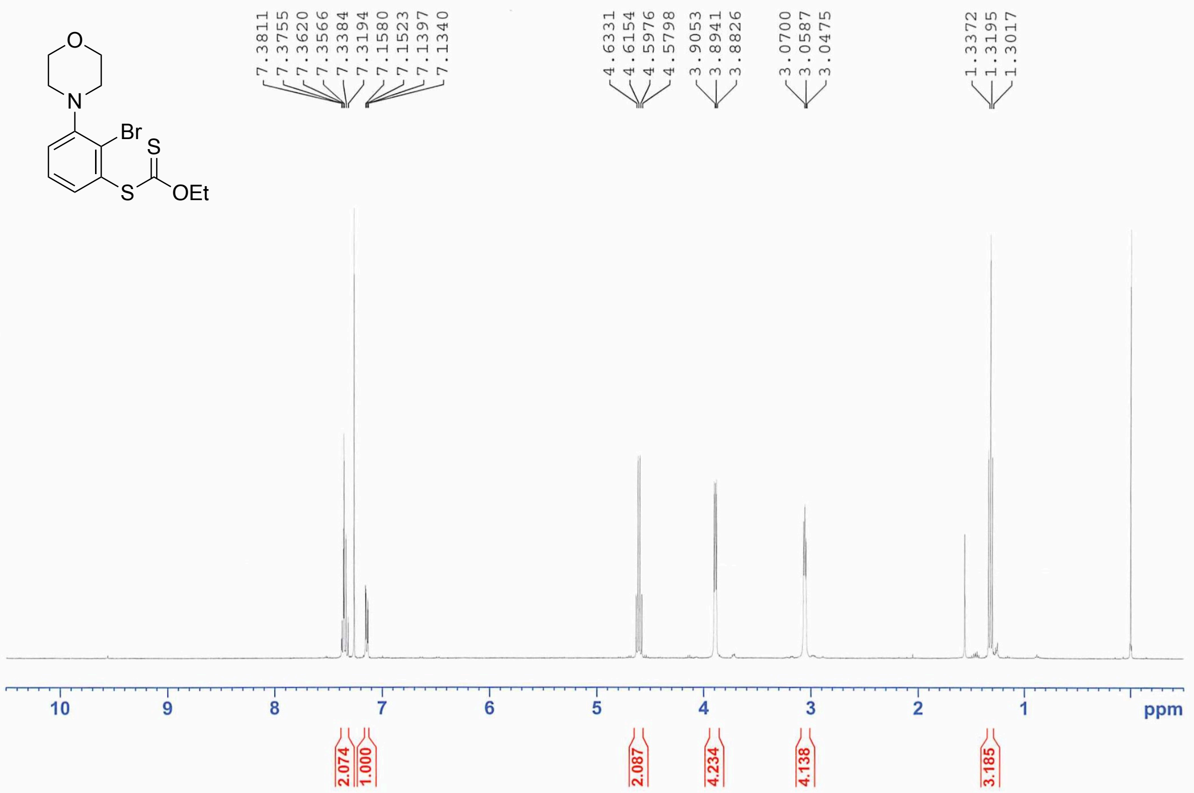

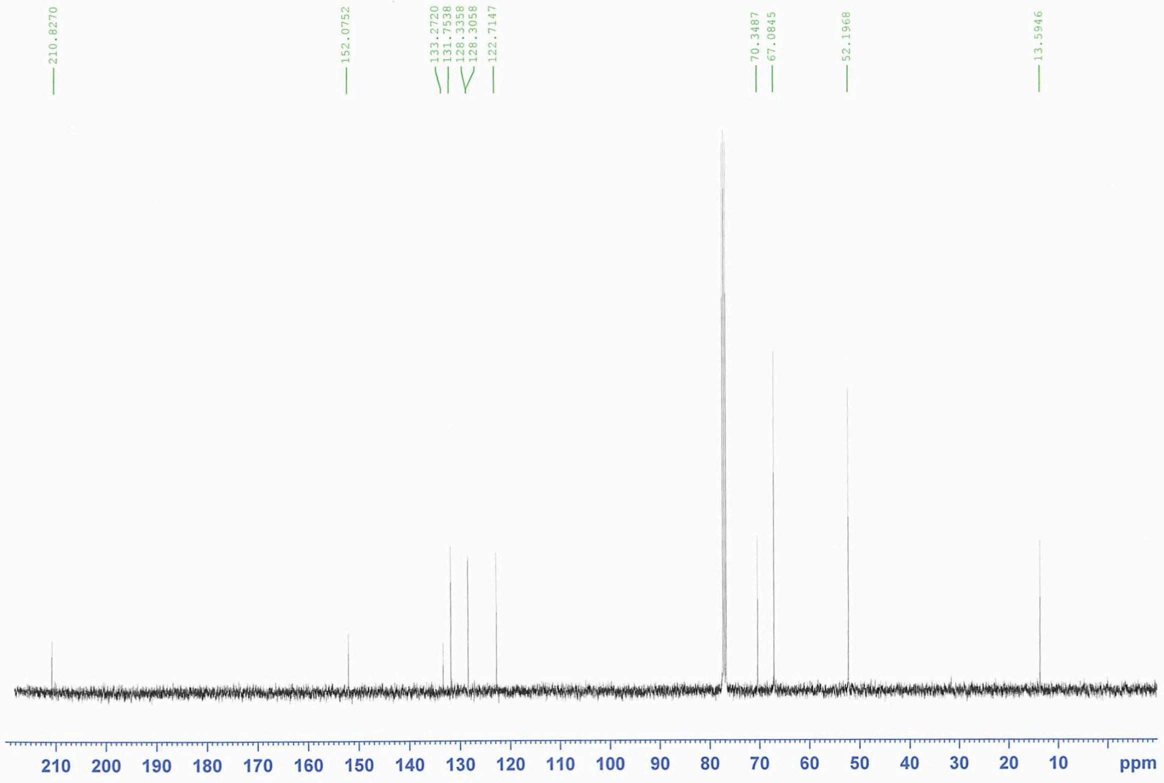

^{1}H NMR (400 MHz) and ^{13}C NMR (101 MHz) spectra of *S*-(2-bromo-3-((4-methoxyphenyl)thio)phenyl) *O*-ethyl carbonodithioate (**8i**) ($CDCl_3$)

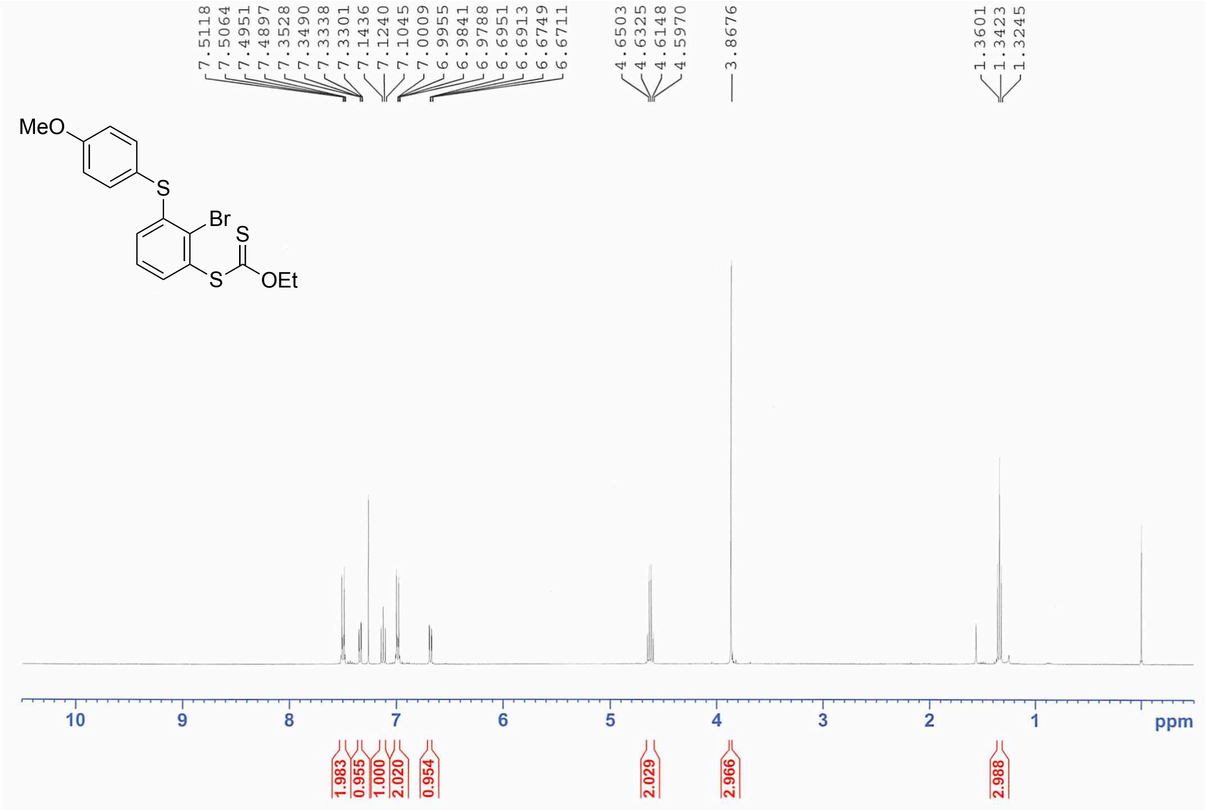

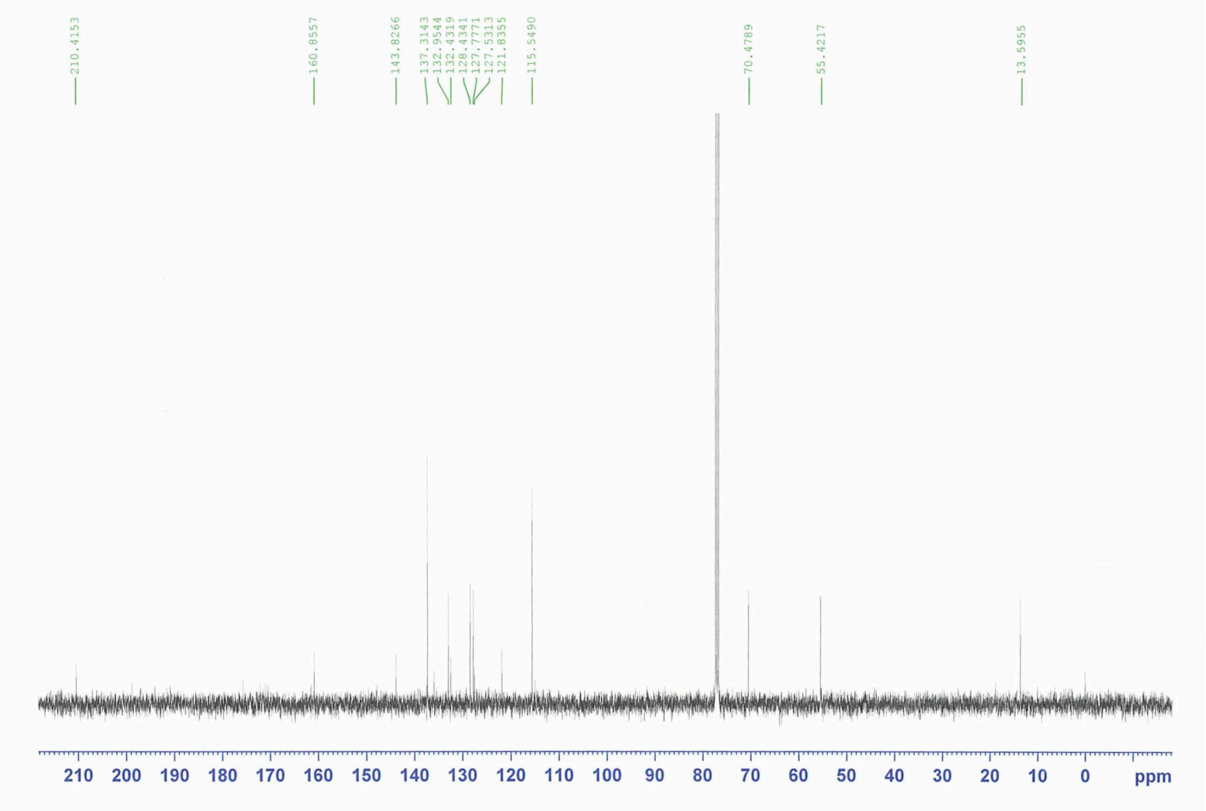

^{1}H NMR (400 MHz) and ^{13}C NMR (101 MHz) spectra of *S*-(7-bromo-1-methyl-1*H*-indol-6-yl) *O*-ethyl carbonodithioate (**8j**) ($CDCl_3$)

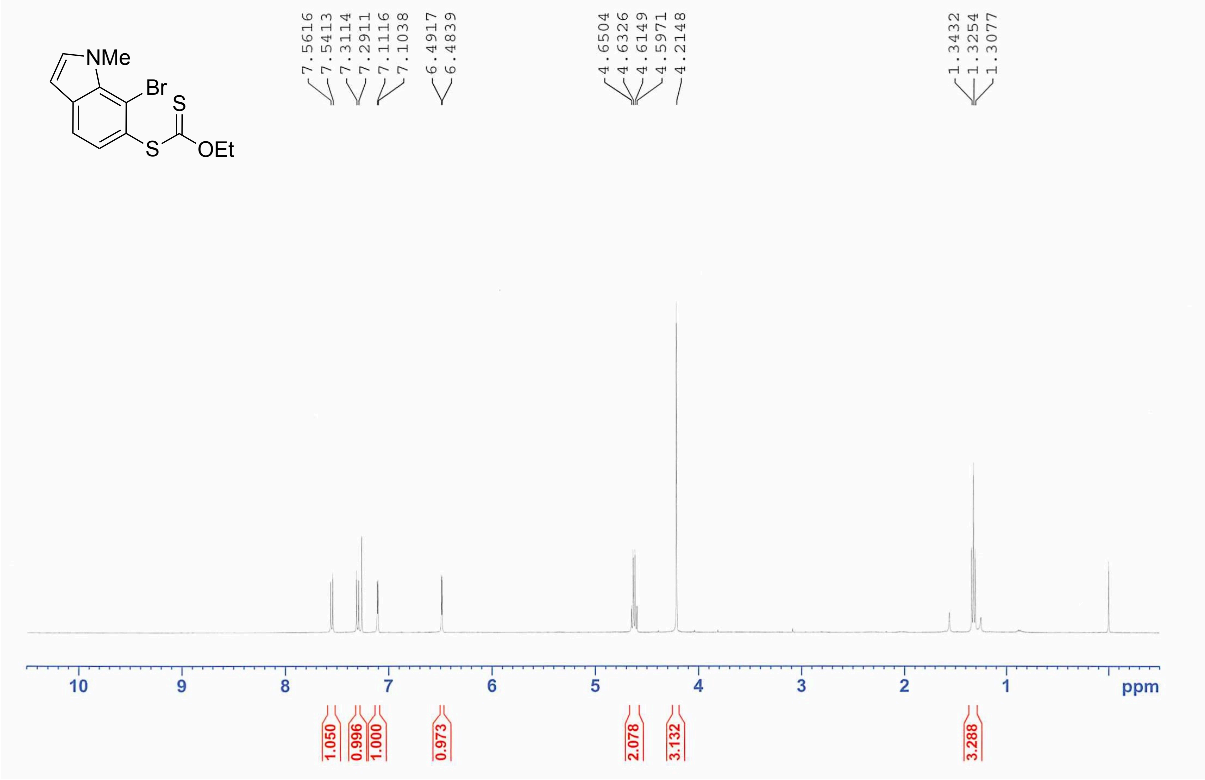

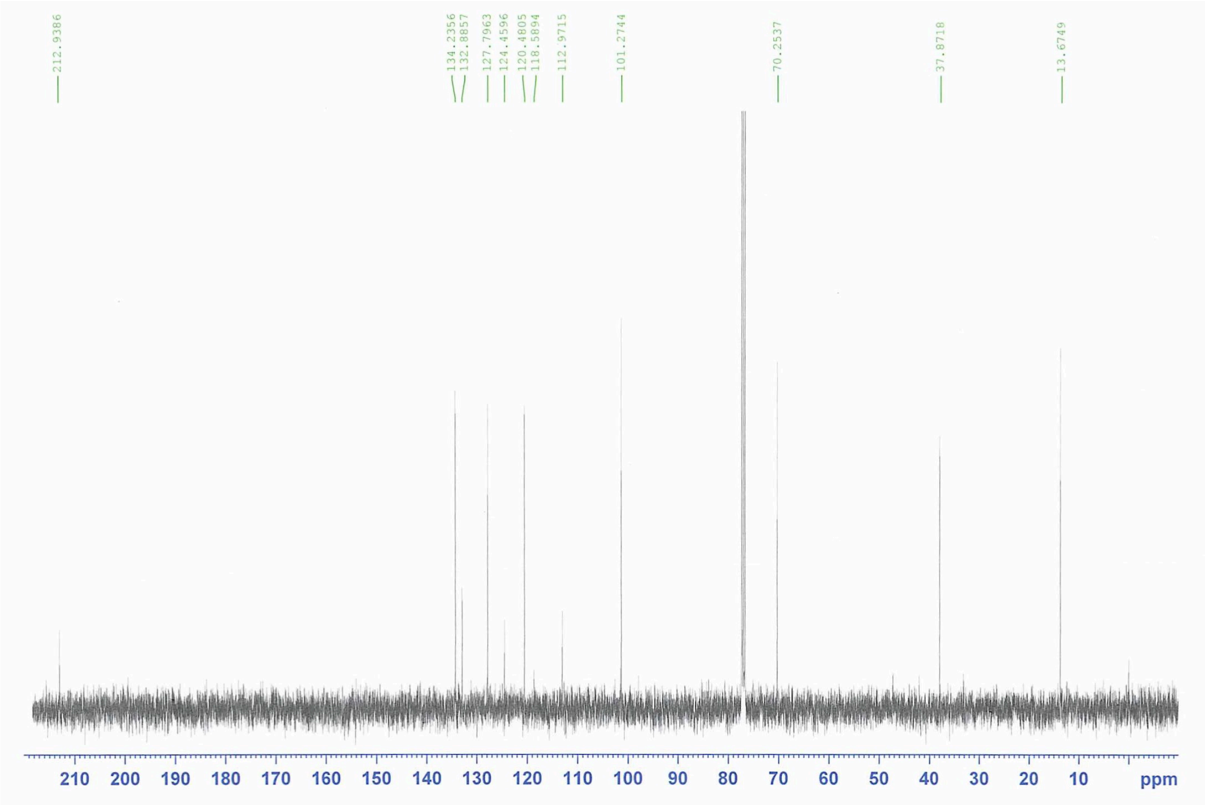

^{1}H NMR (400 MHz) and ^{13}C NMR (101 MHz) spectra of *S*-(2-bromo-4,5-dimethoxyphenyl) *O*-(4-chlorophenethyl) carbonodithioate (**8k**) ($CDCl_3$)

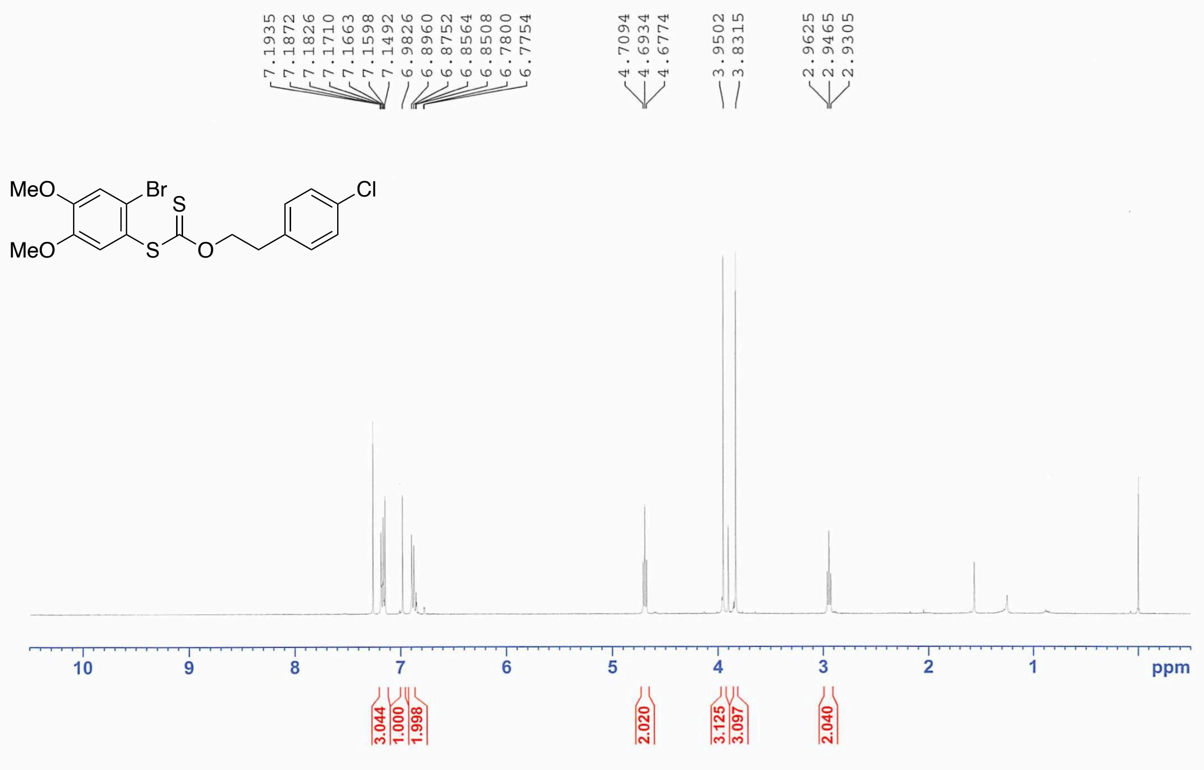

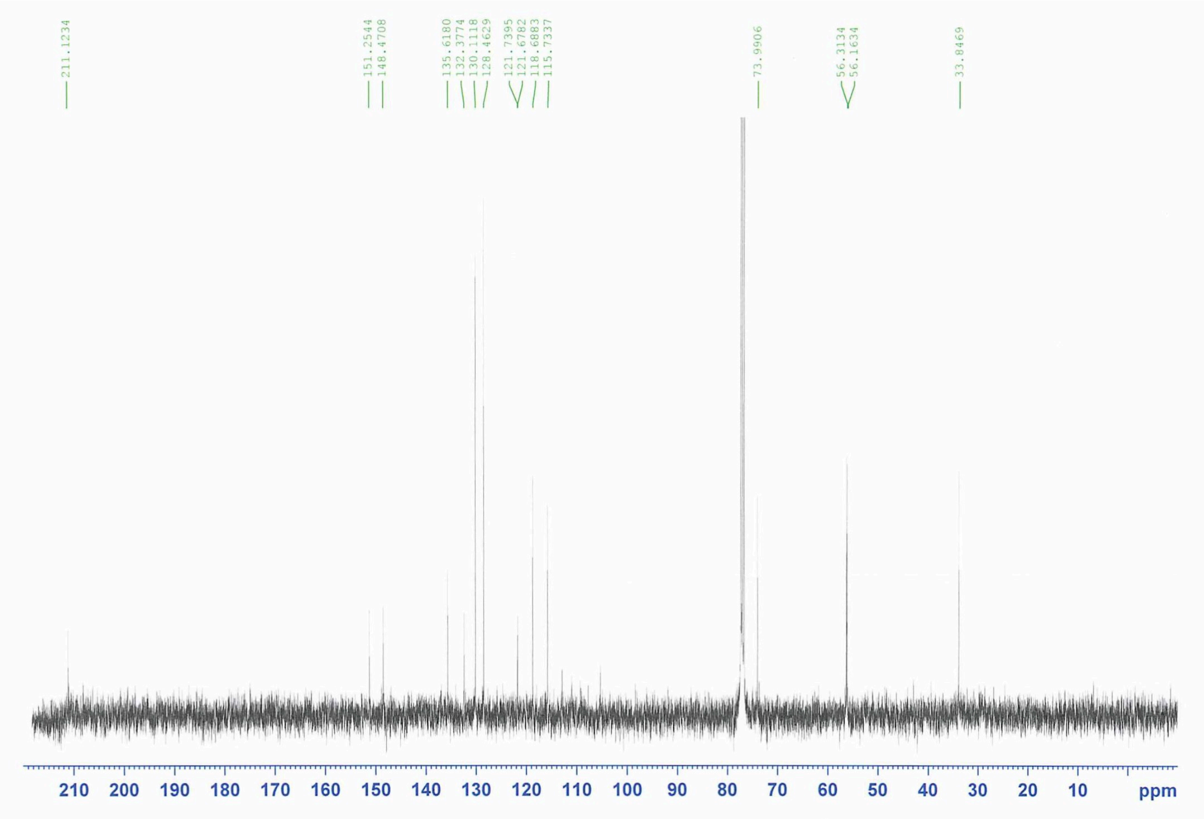

^{1}H NMR (400 MHz) and ^{13}C NMR (101 MHz) spectra of *O*-benzyl *S*-(2-bromo-4,5-dimethoxyphenyl) carbonodithioate (**8l**) ($CDCl_3$)

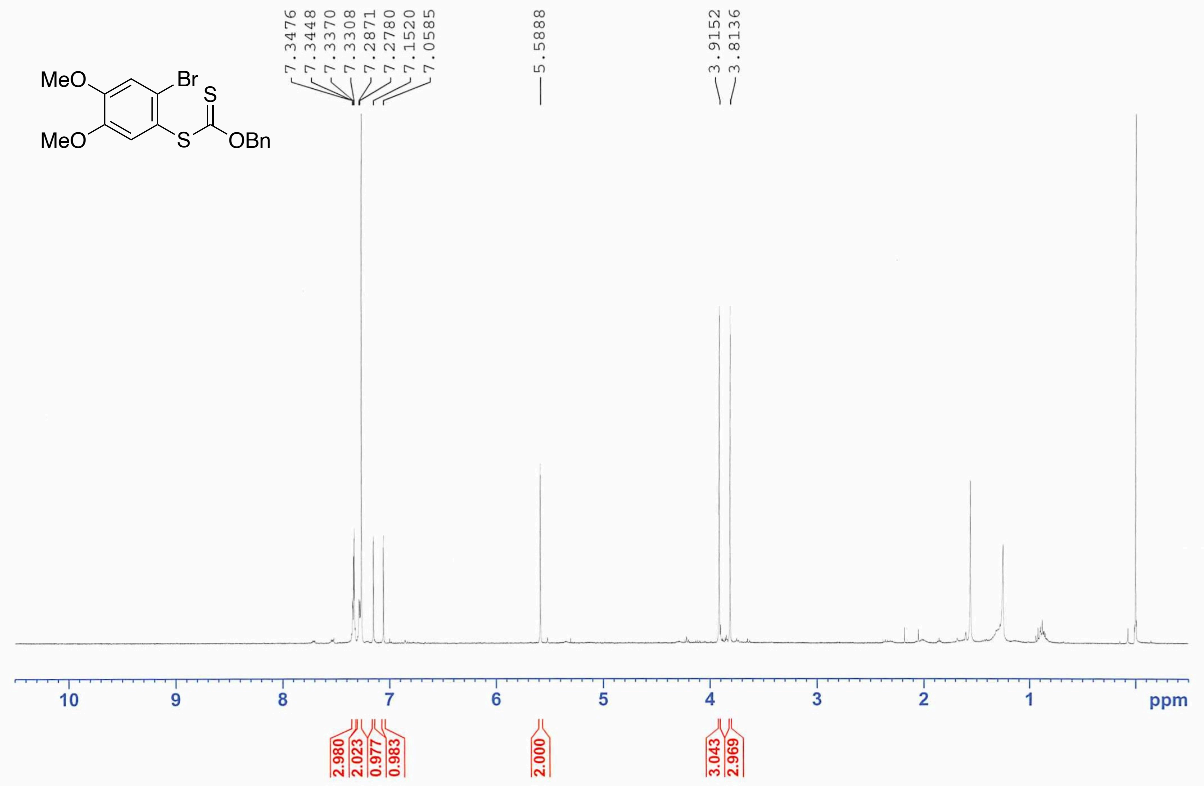

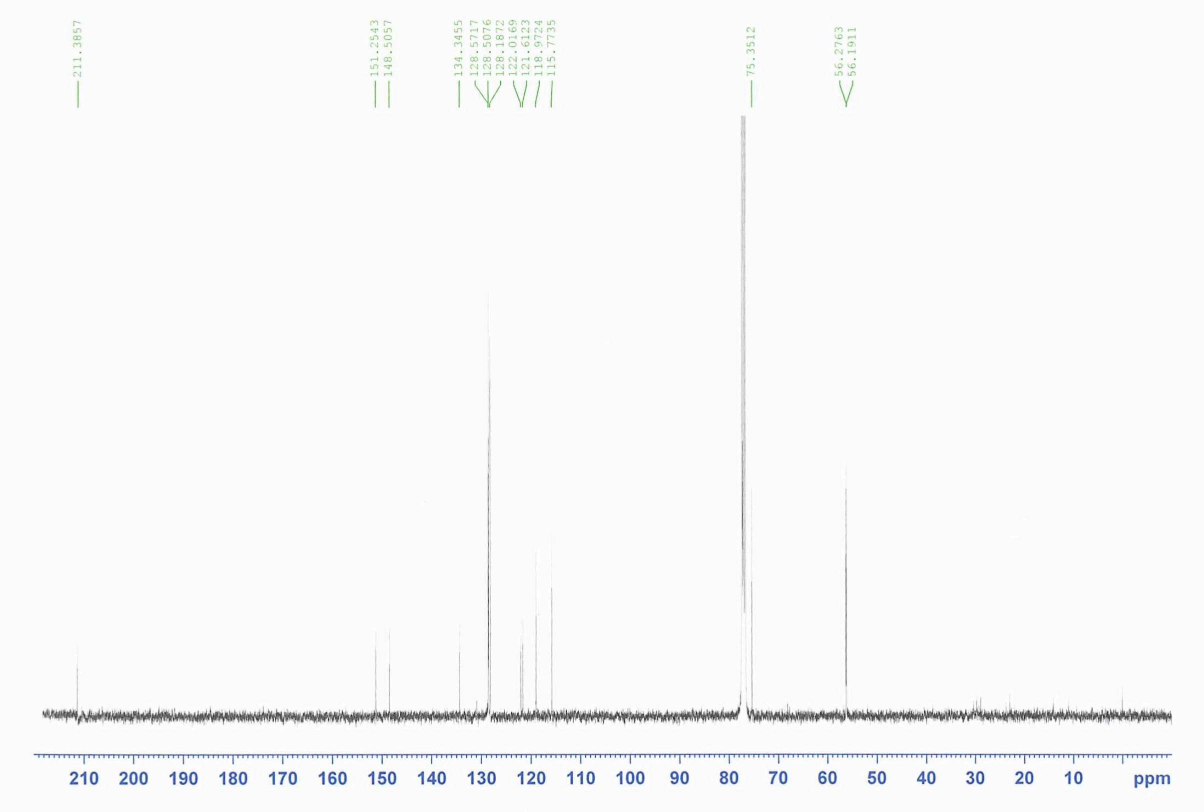

^{1}H NMR (400 MHz) and ^{13}C NMR (101 MHz) spectra of *S*-(2-bromo-4,5-dimethoxyphenyl) *O*-isopropyl carbonodithioate (**8m**) ($CDCl_3$)

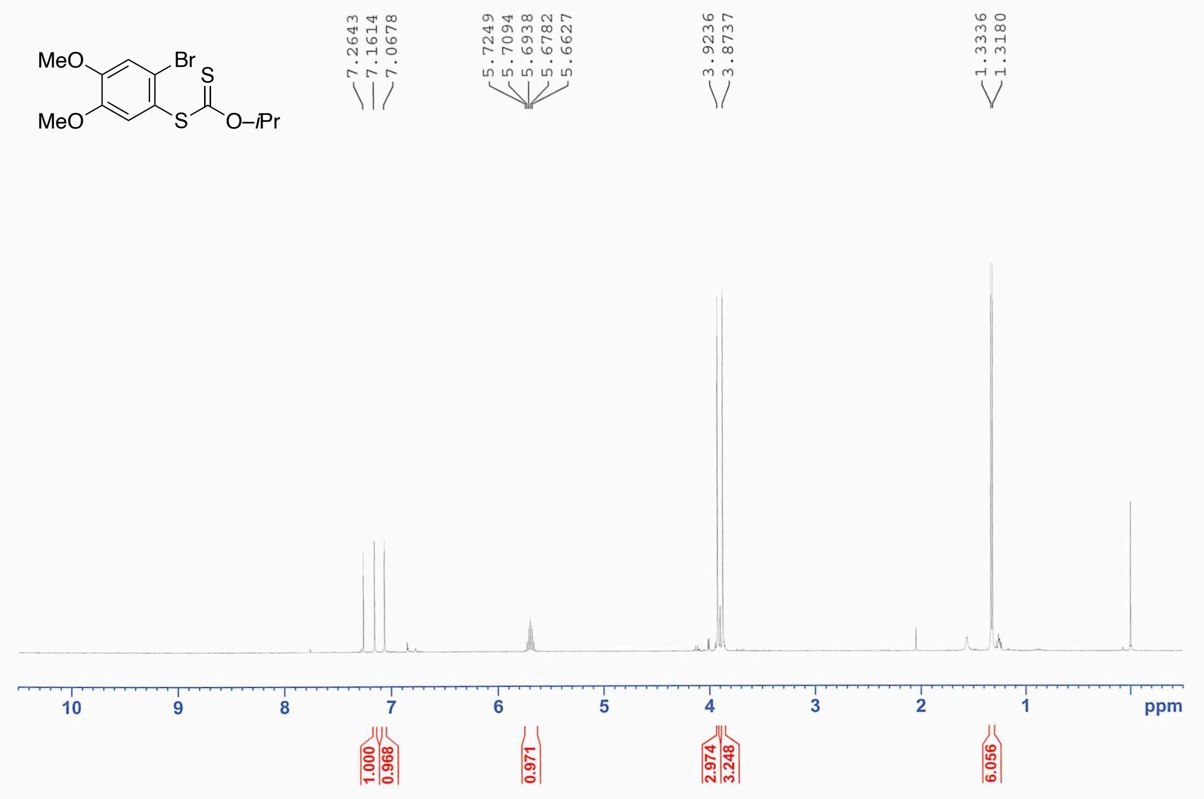

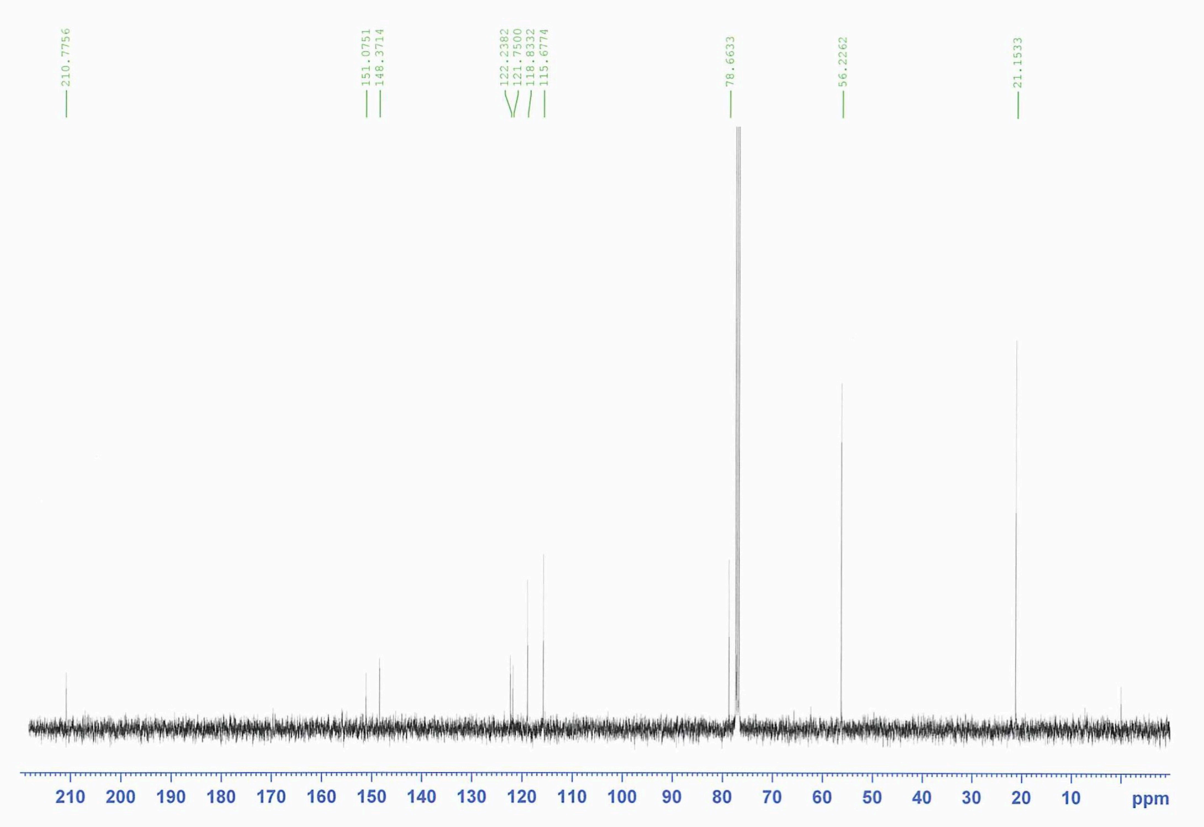

^{1}H NMR (400 MHz) and ^{13}C NMR (101 MHz) spectra of *S*-(2-bromo-4,5-dimethoxyphenyl) *O*-(pent-4-en-1-yl) carbonodithioate (**8n**) ($CDCl_3$)

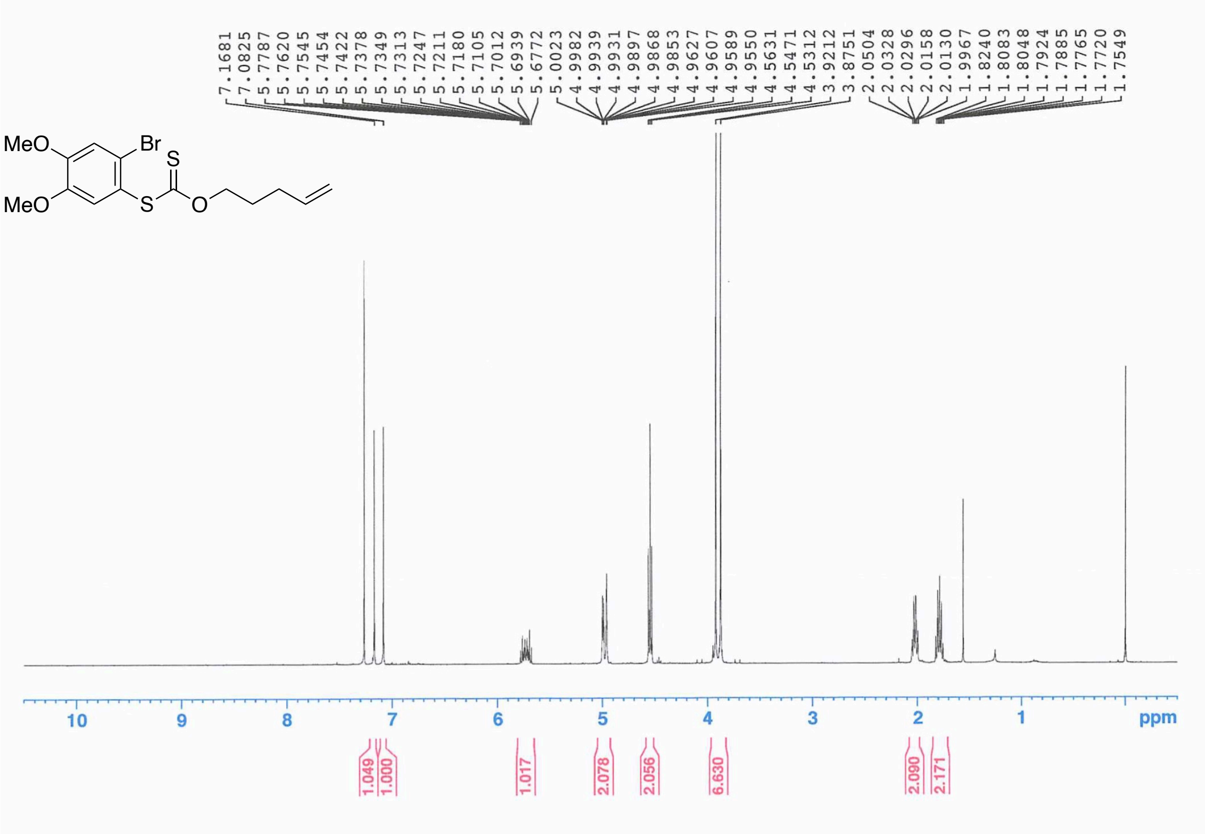

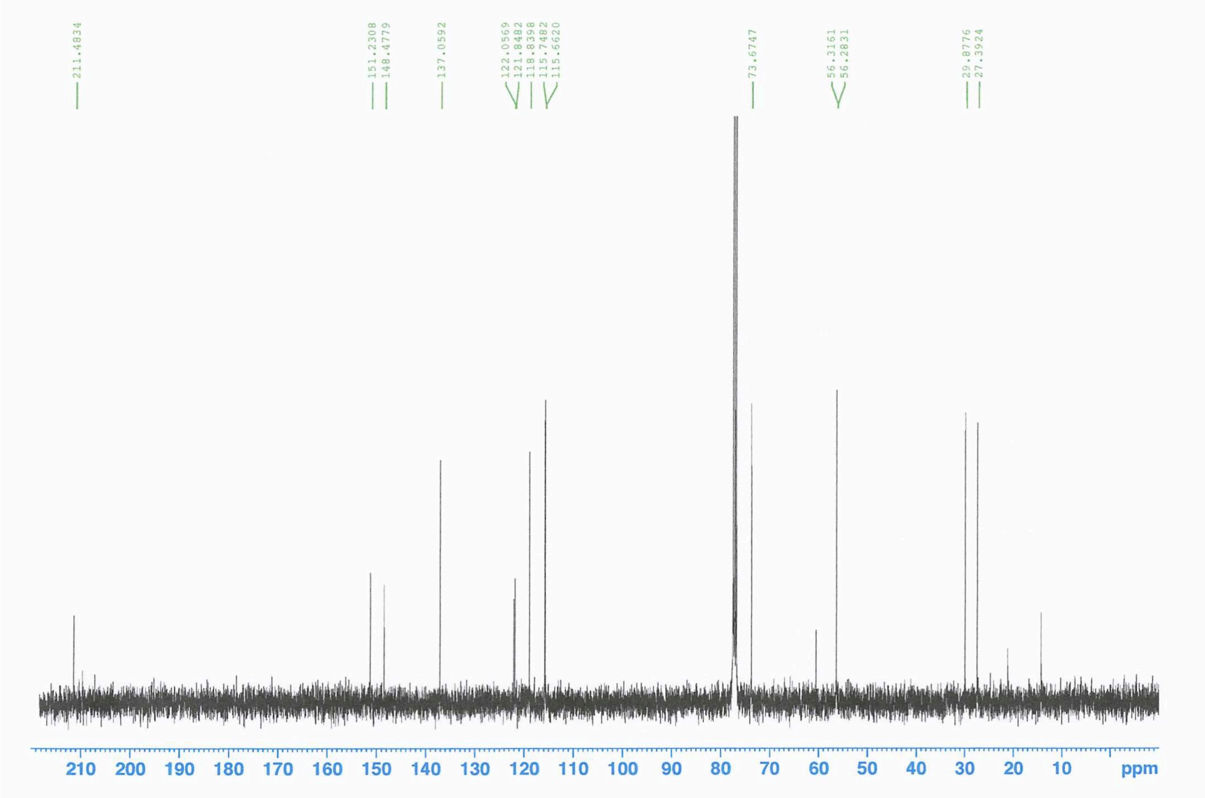

^{1}H NMR (400 MHz) and ^{13}C NMR (101 MHz) spectra of *S*-(2-bromo-4,5-dimethoxyphenyl) *N*,*N*-diethylcarbamodithioate (**8o**) ($CDCl_3$)

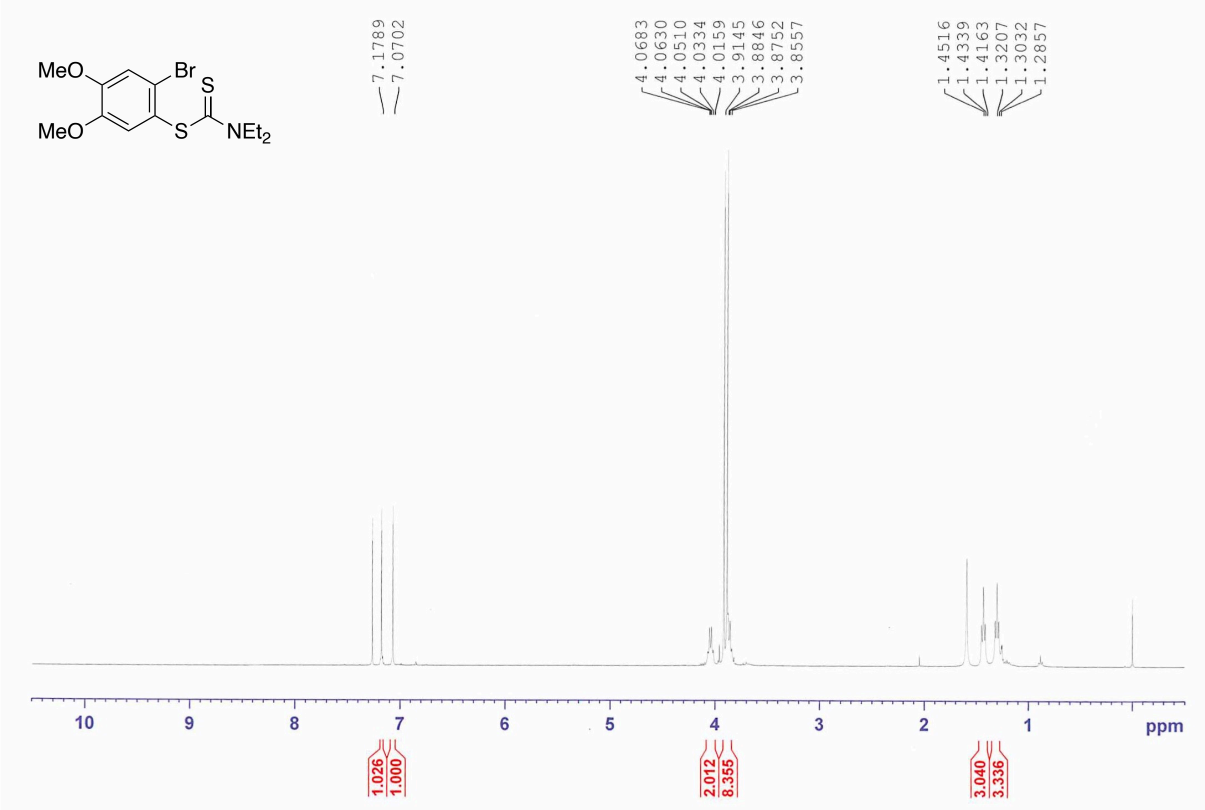

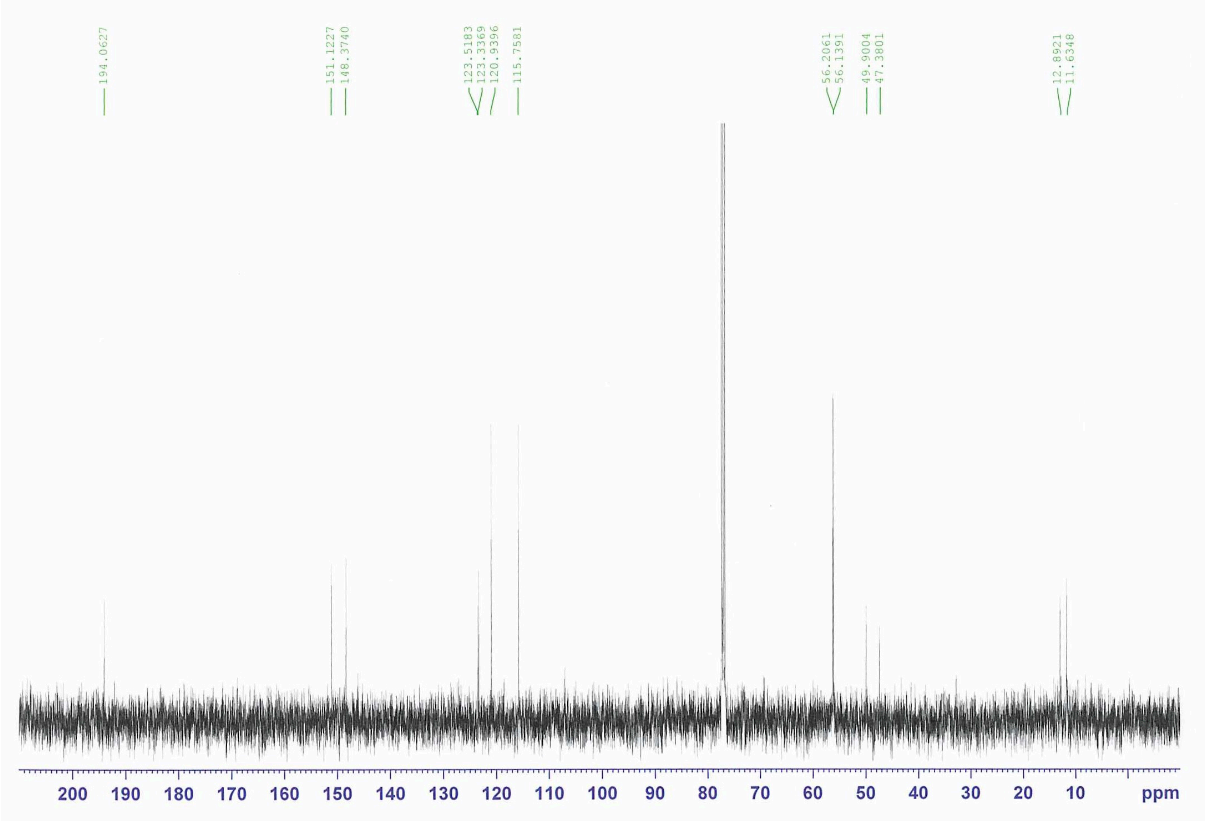

^{1}H NMR (400 MHz) and ^{13}C NMR (101 MHz) spectra of 2-bromo-4,5-dimethoxyphenyl benzyl(methyl)carbamodithioate (**8p**) ($CDCl_3$)

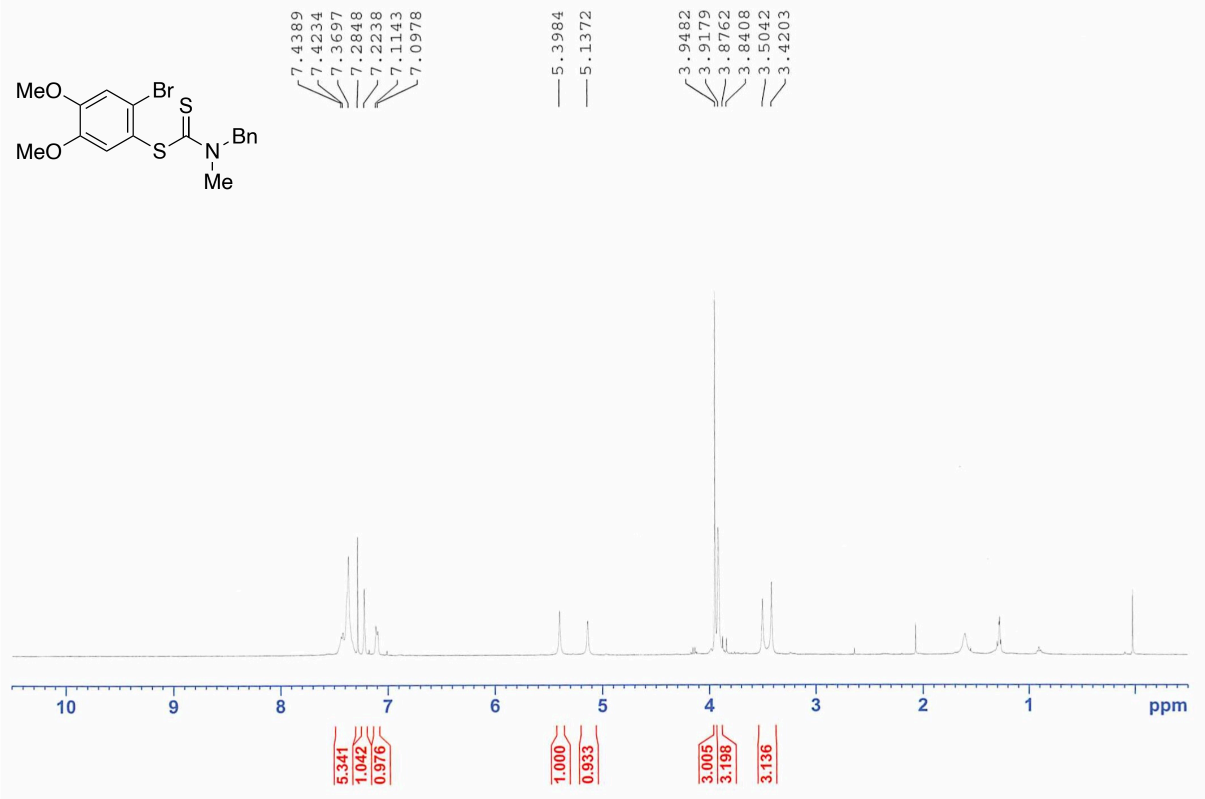

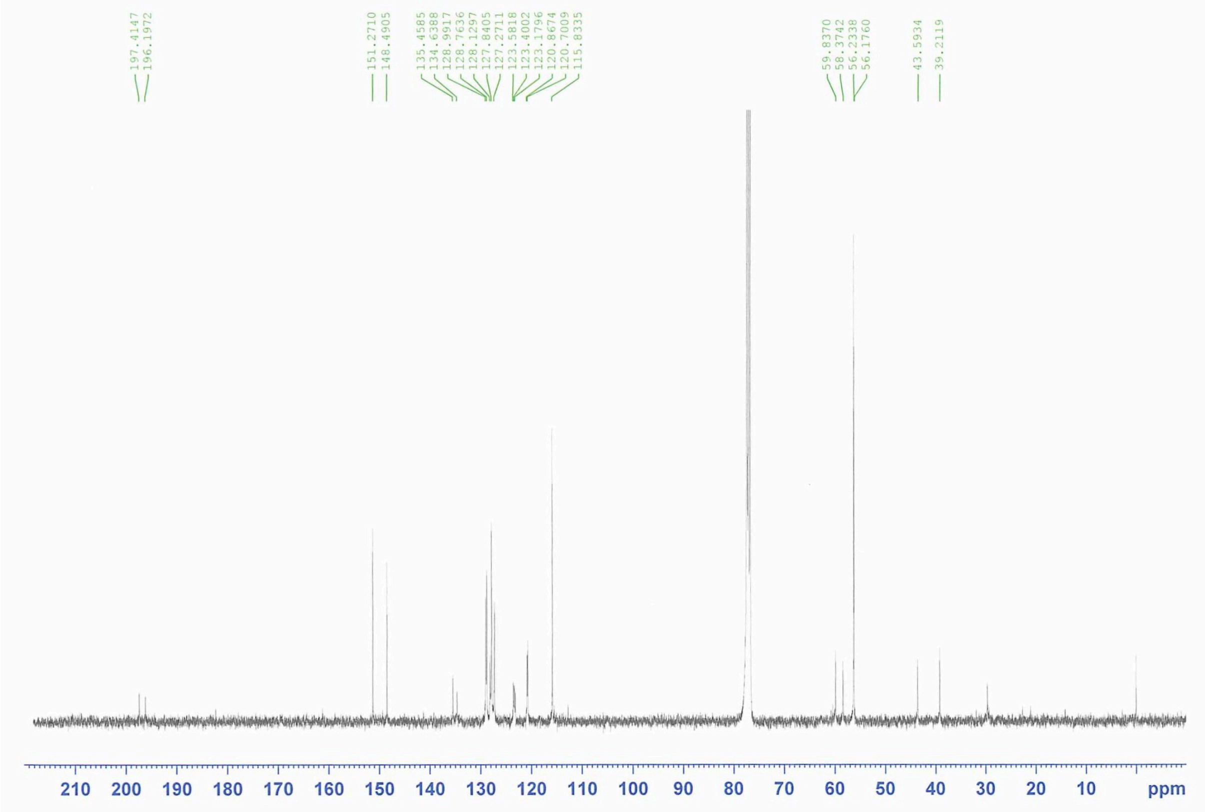

^{1}H NMR (400 MHz) and ^{13}C NMR (101 MHz) spectra of *O*-ethyl *S*-(2-iodo-4,5-dimethoxyphenyl) carbonodithioate (**10**) ($CDCl_3$)

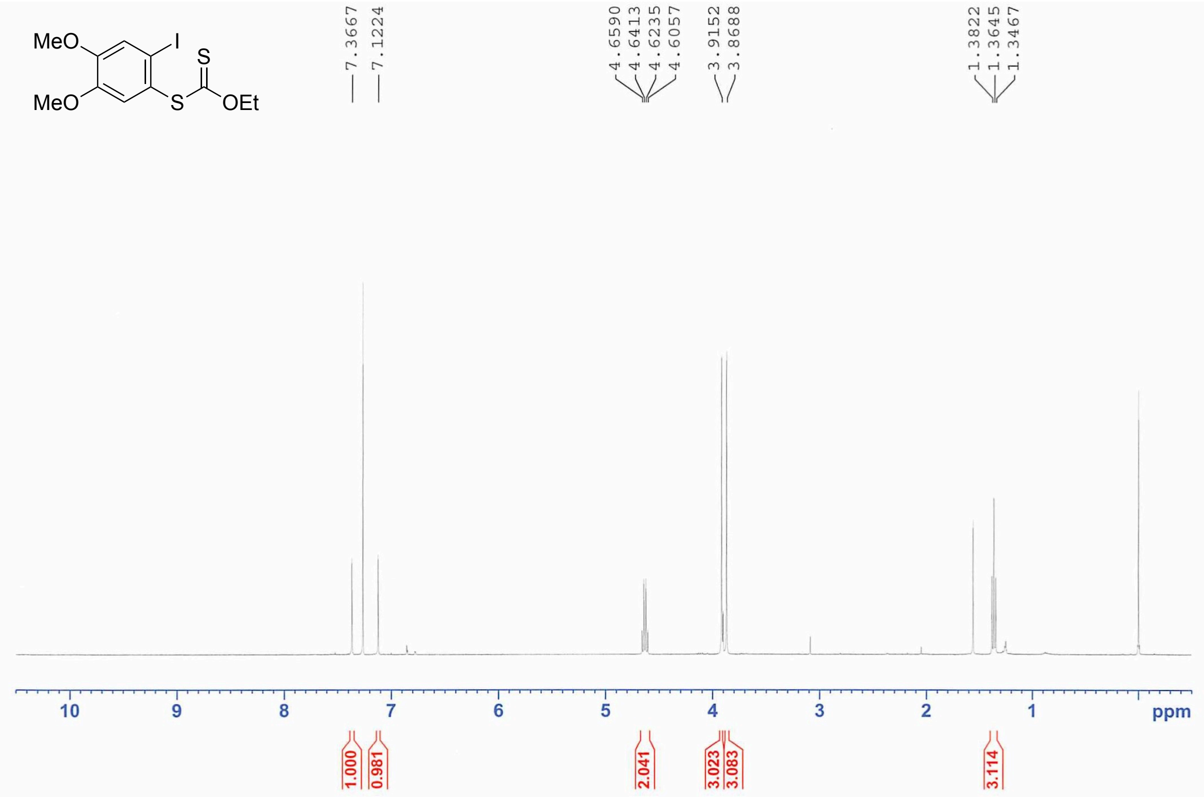

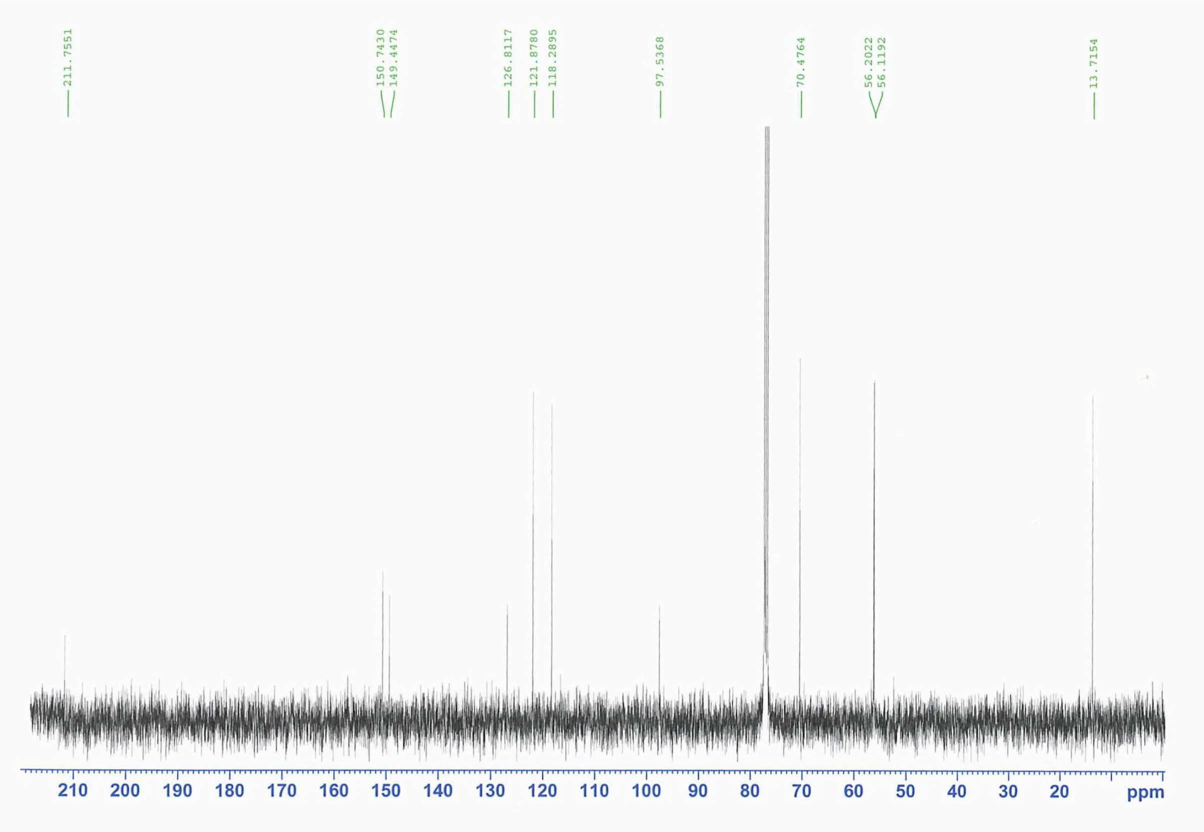

^{1}H NMR (400 MHz) and ^{13}C NMR (101 MHz) spectra of 3,4-dimethoxyphenyl 2-nitrophenyl sulfide (**13b**) ($CDCl_3$)

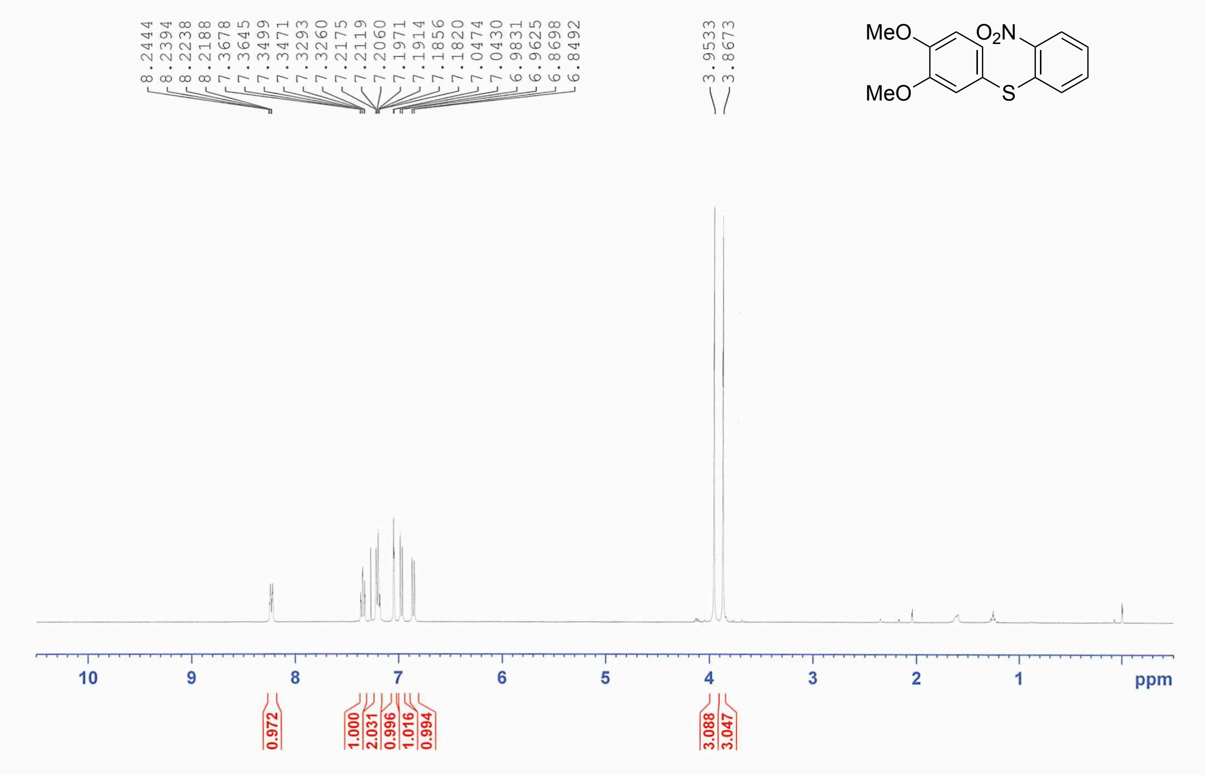

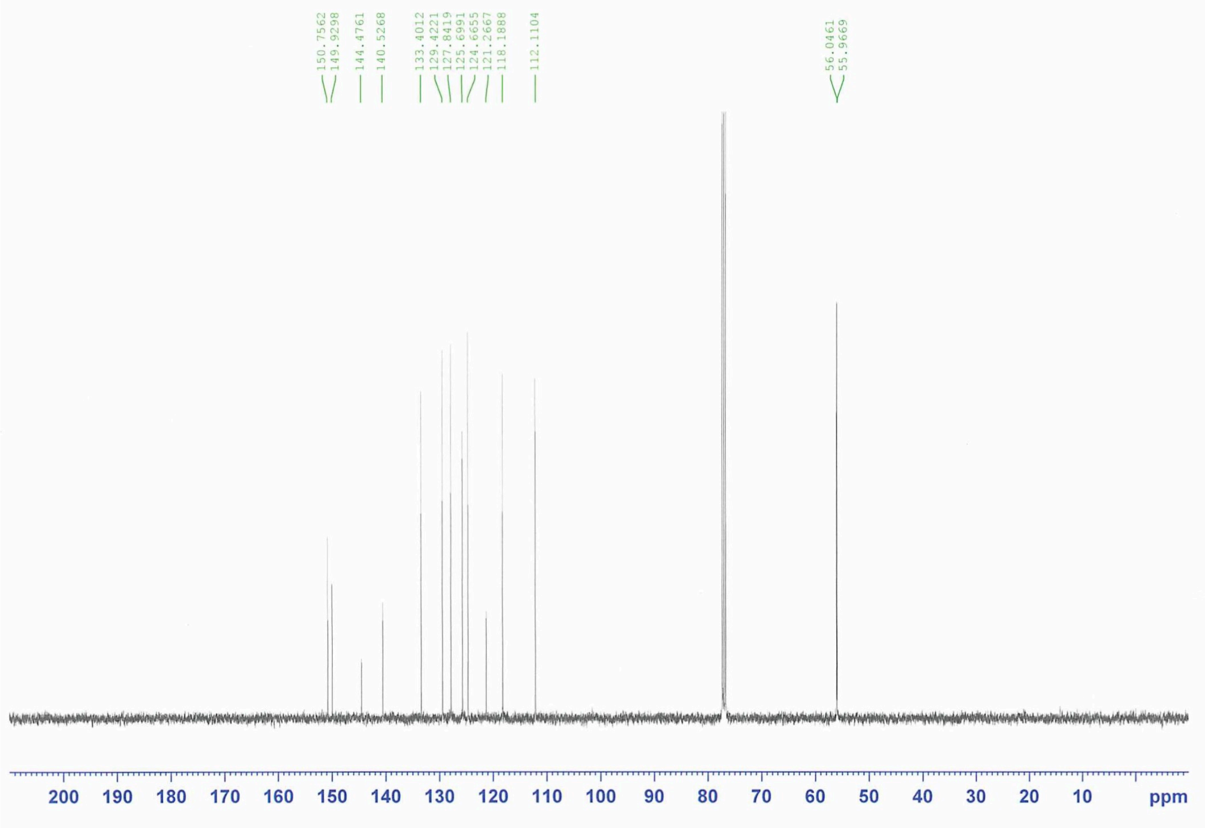

^{1}H NMR (400 MHz) and ^{13}C NMR (101 MHz) spectra of 2-bromo-4,5-difluorophenyl 2-bromonaphthalen-1-yl sulfide (**16**) ($CDCl_3$)

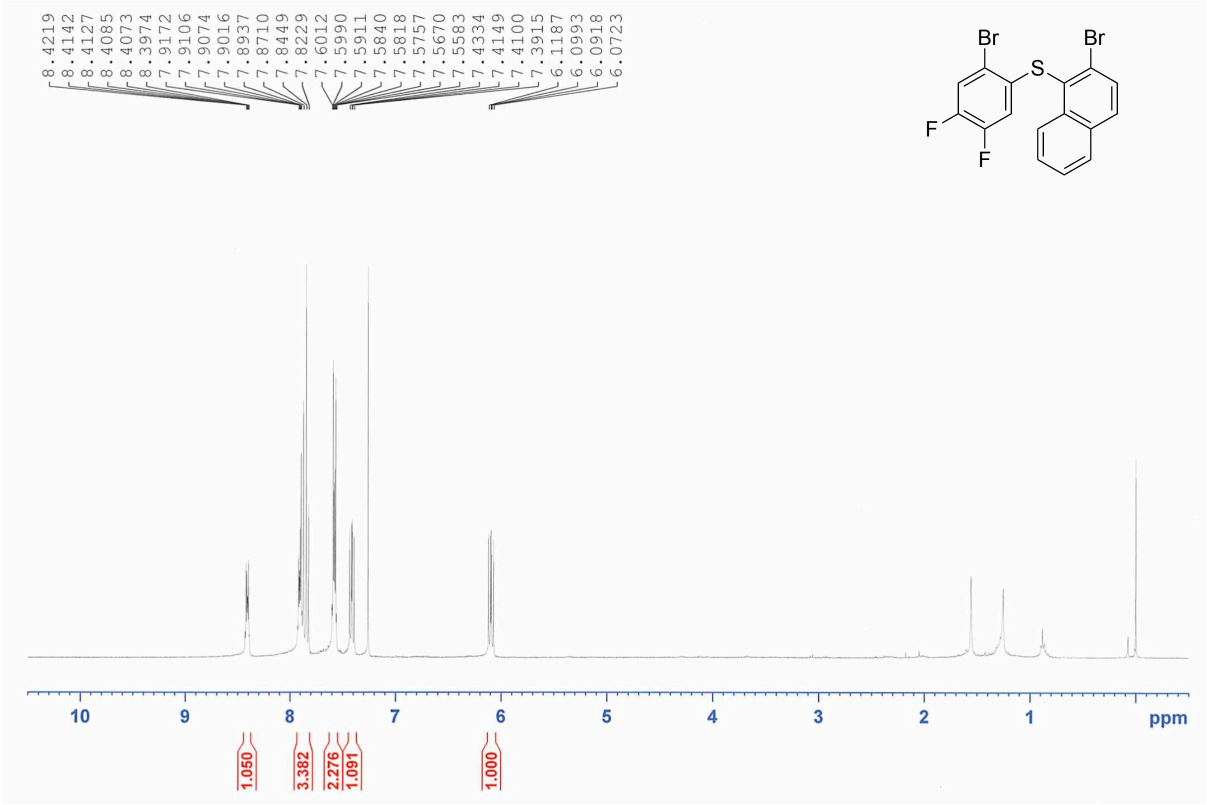

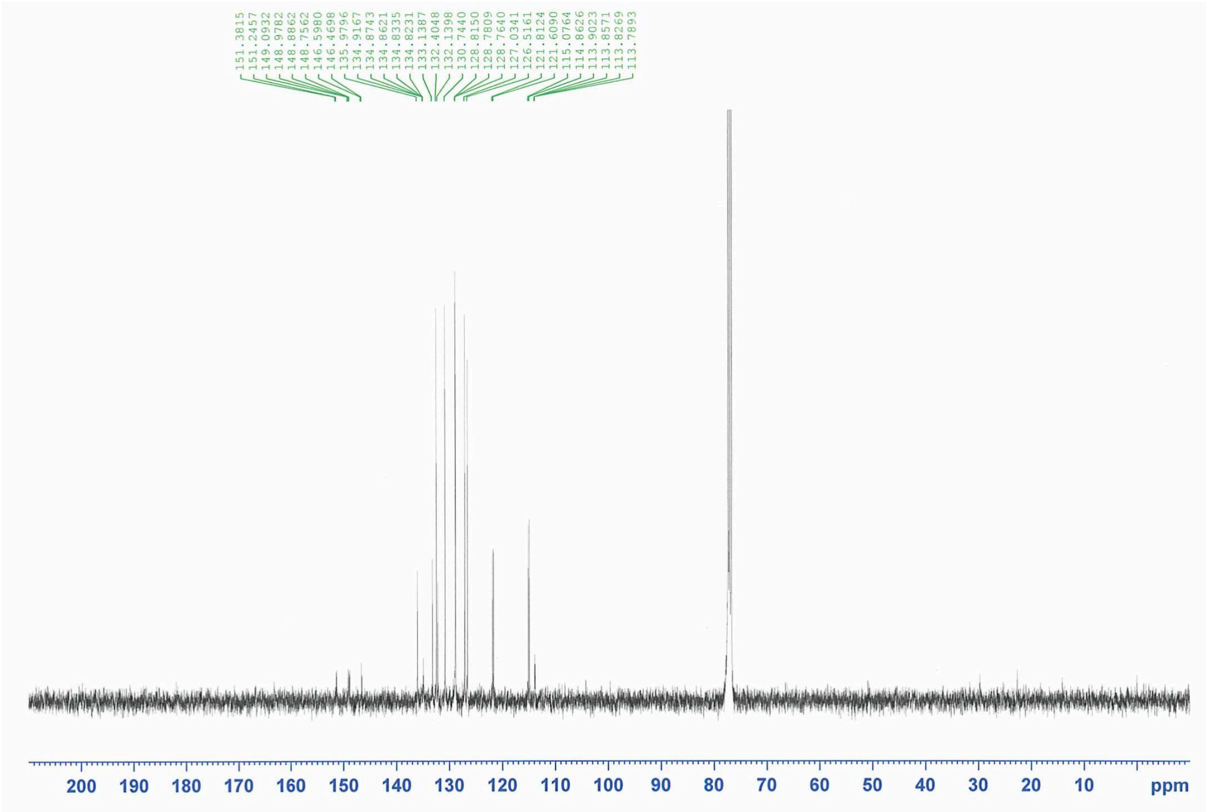

^{1}H NMR (400 MHz) and ^{13}C NMR (101 MHz) spectra of 9,10-difluoro-7-phenyl-7*H*-benzo[*c*]phenothiazine (**17**) (Acetone-d_6)

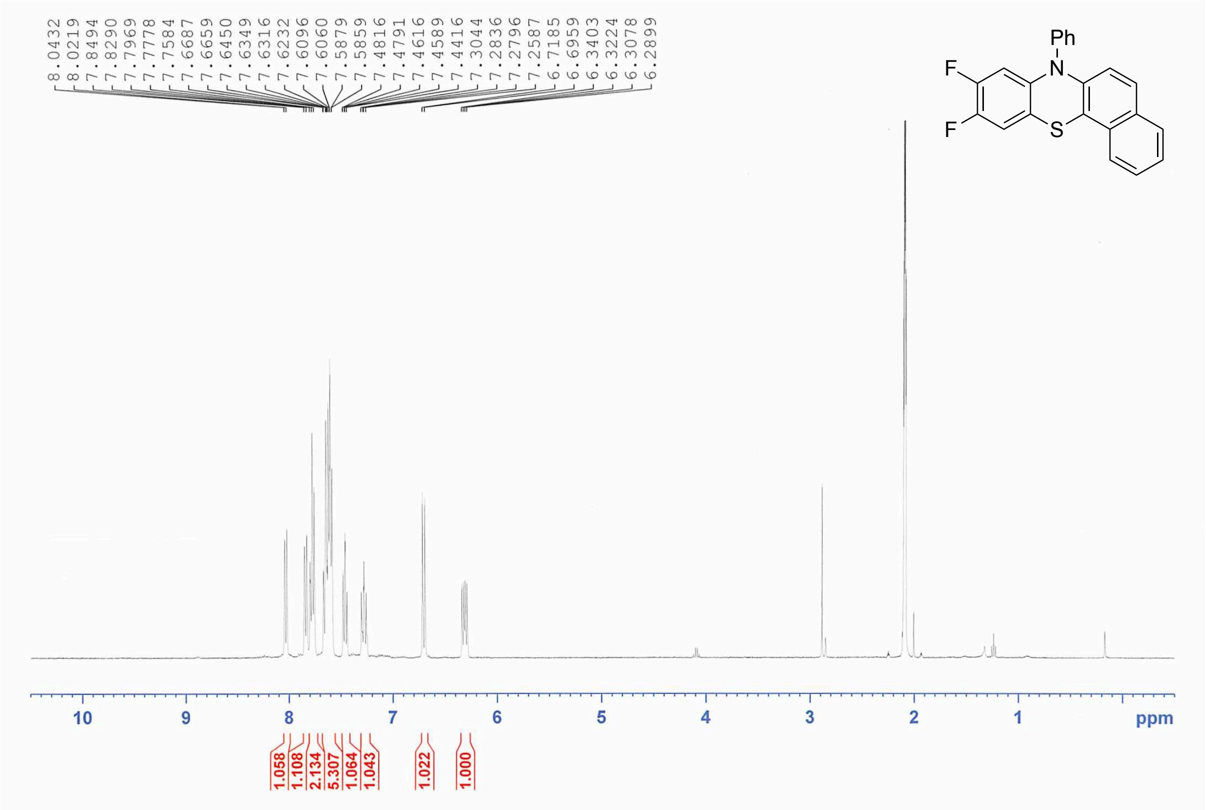

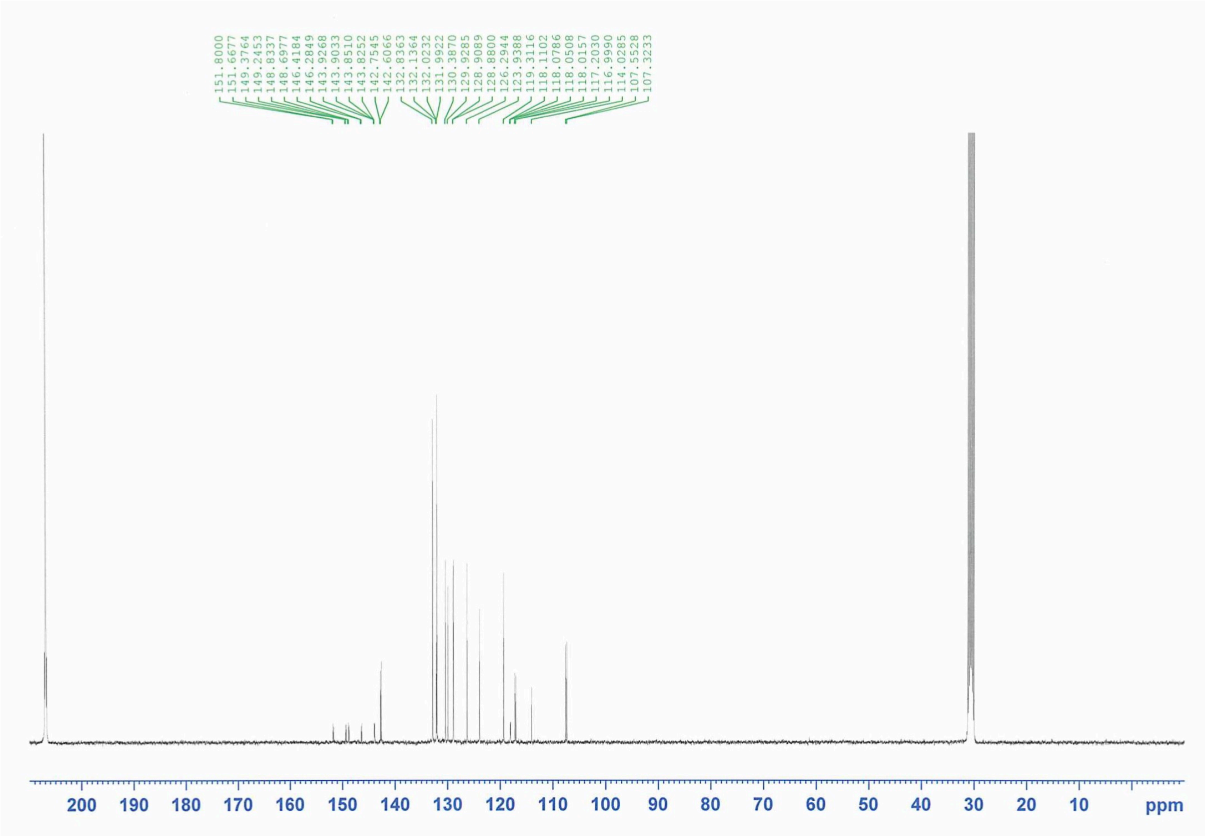

^{1}H NMR (400 MHz, $CDCl_3$) and ^{13}C NMR (101 MHz, Acetone-d_6,) spectra of 9,10-difluorobenzo[*a*]thianthrene (**18**)

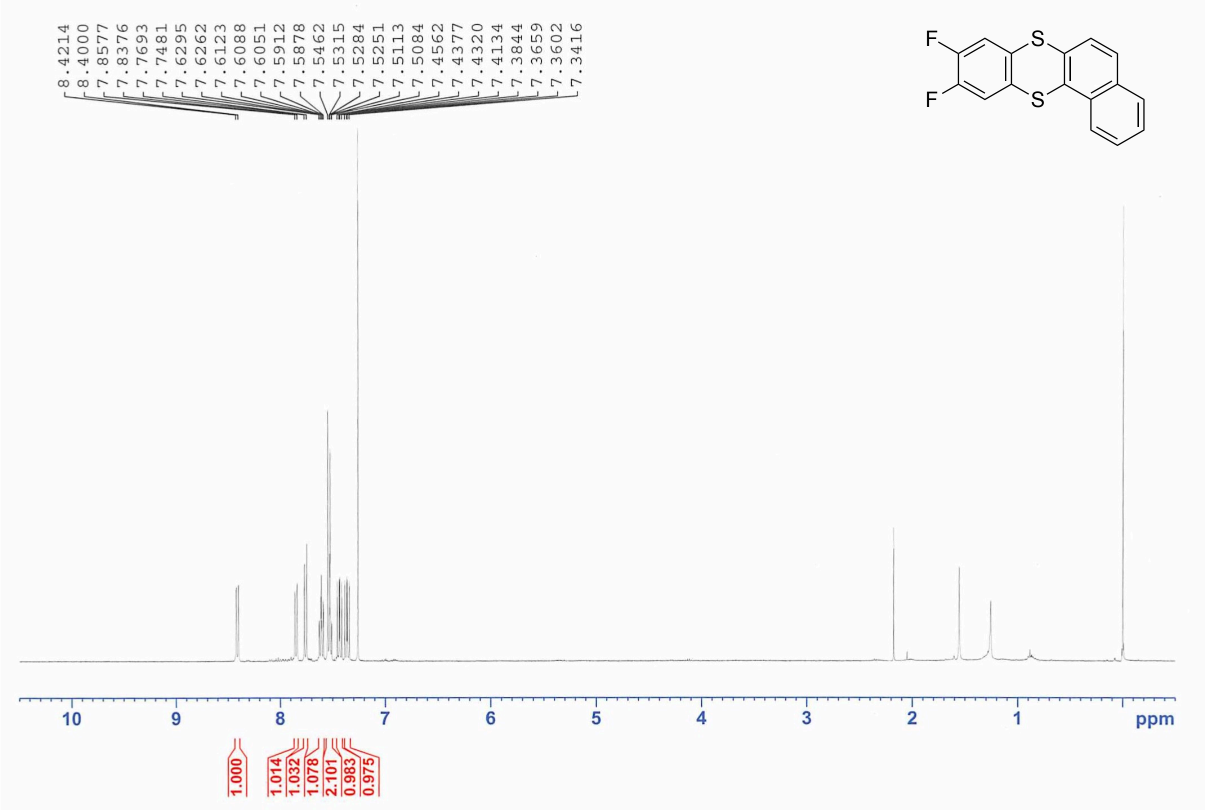

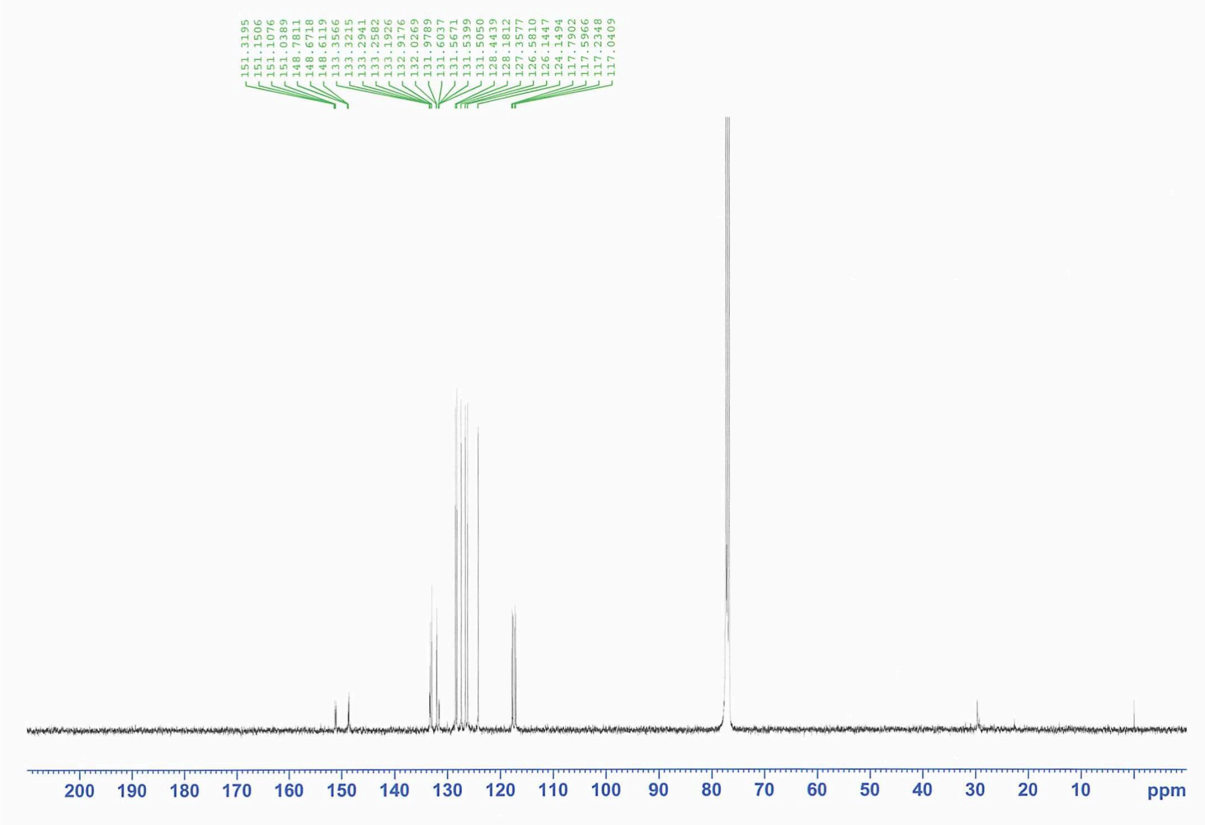

^{1}H NMR (400 MHz) and ^{13}C NMR (101 MHz) spectra of potassium *O*-(4-chlorophenethyl) carbonodithioate (**2e**) (DMSO-d_6)

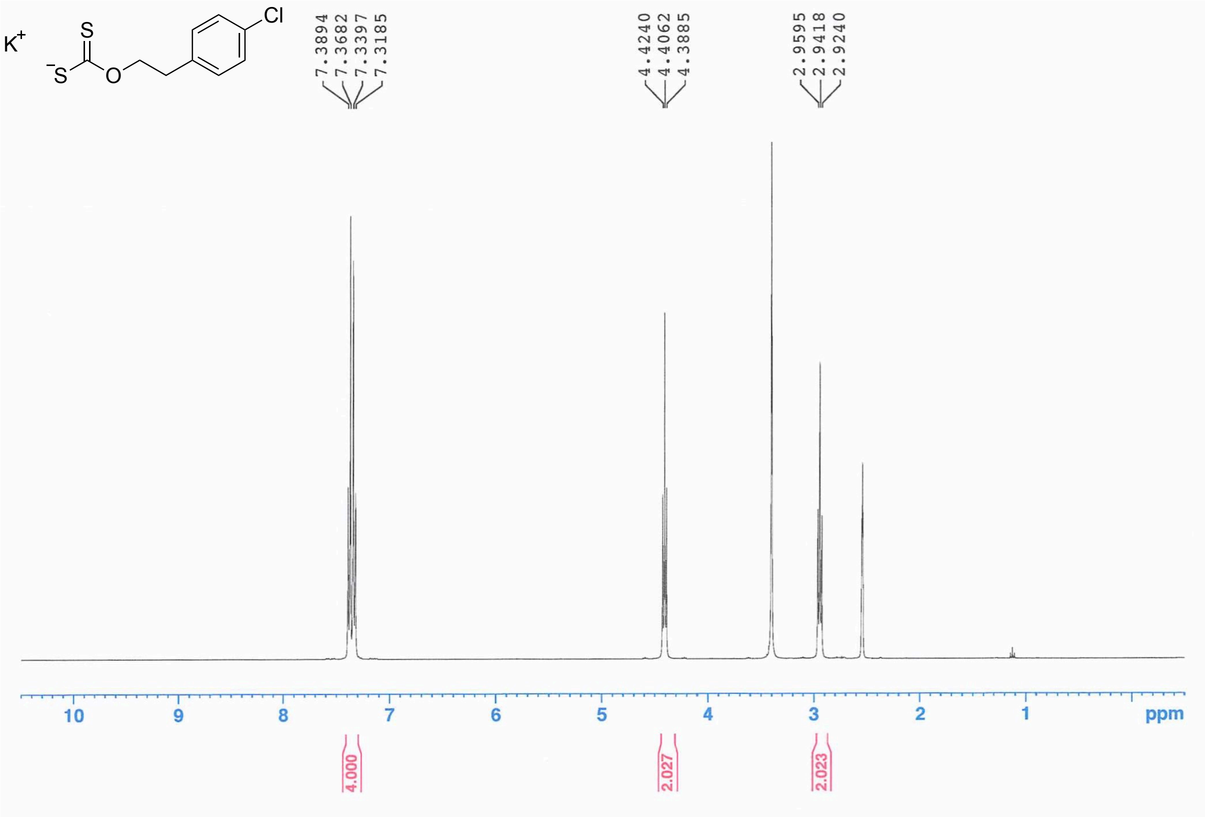

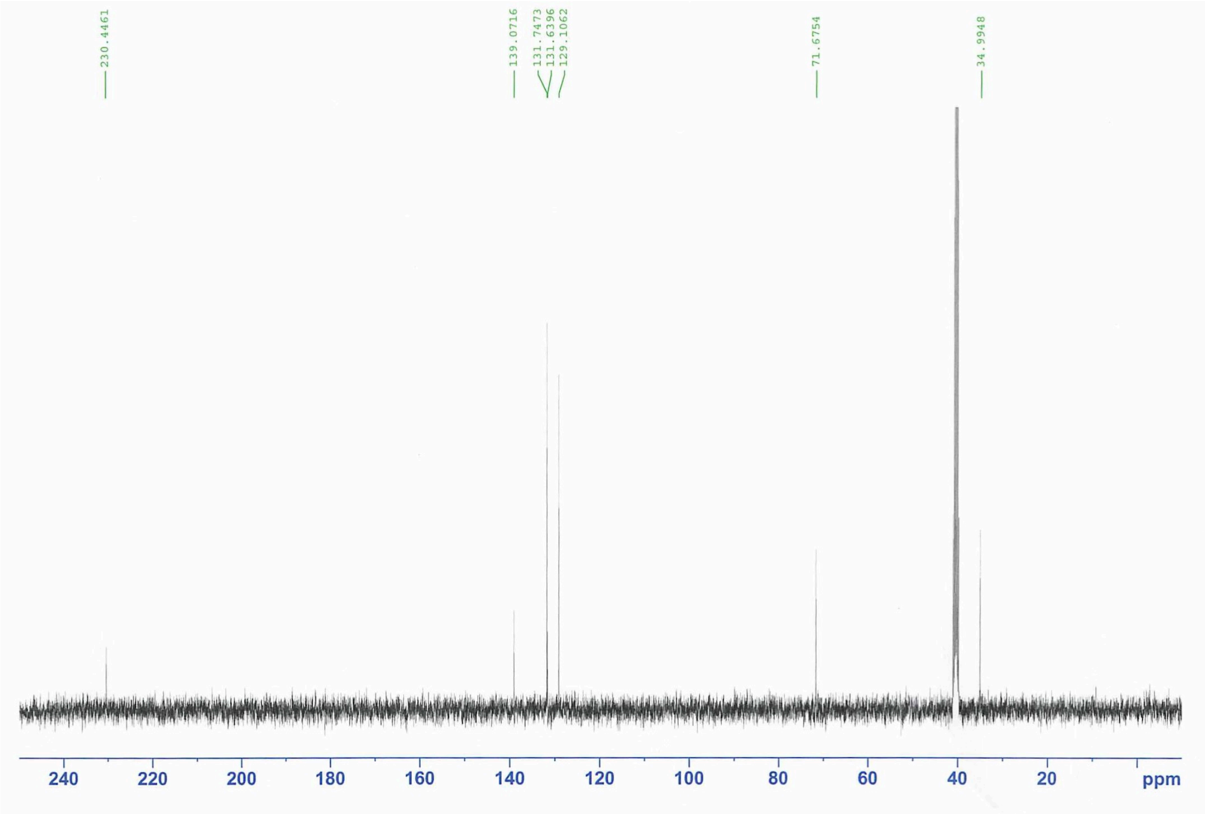

^{1}H NMR (400 MHz) and ^{13}C NMR (101 MHz) spectra of Potassium *O*-(pent-4-en-1-yl) carbonodithioate (**2h**) (DMSO-d_6)

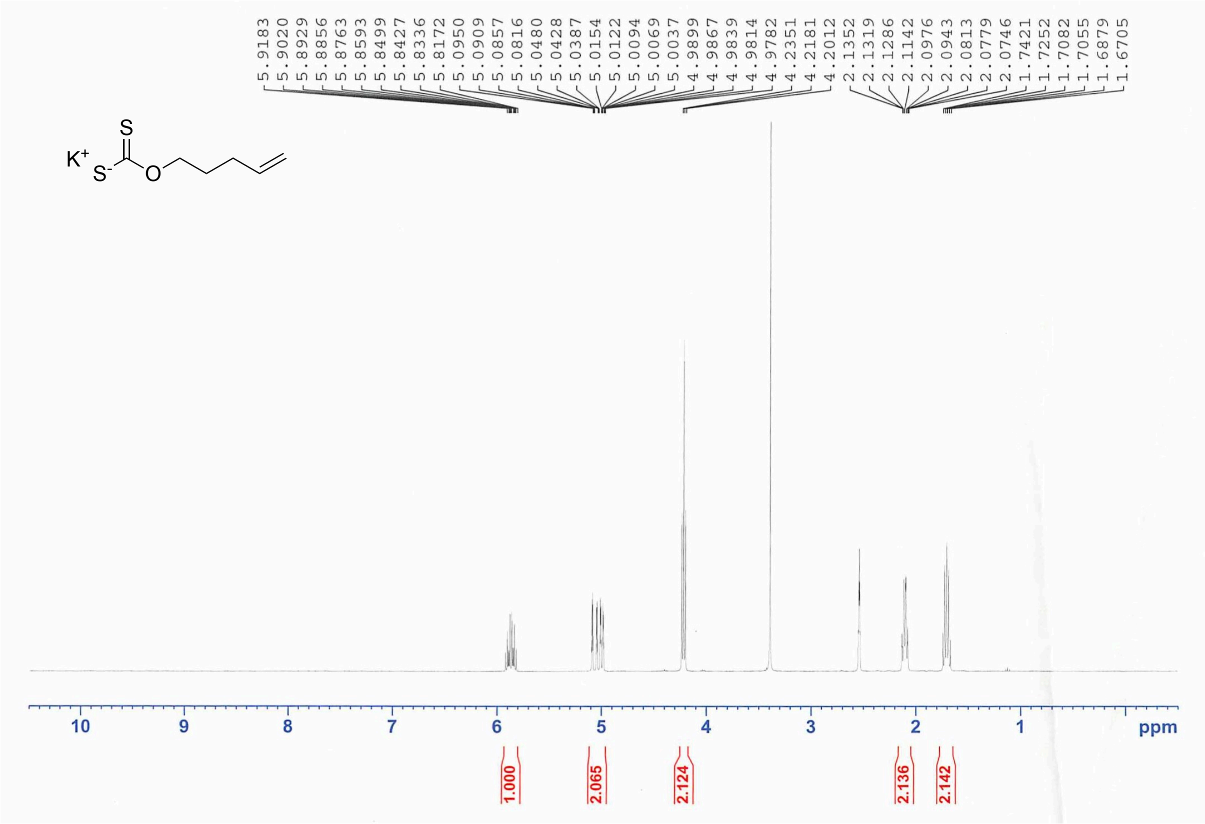

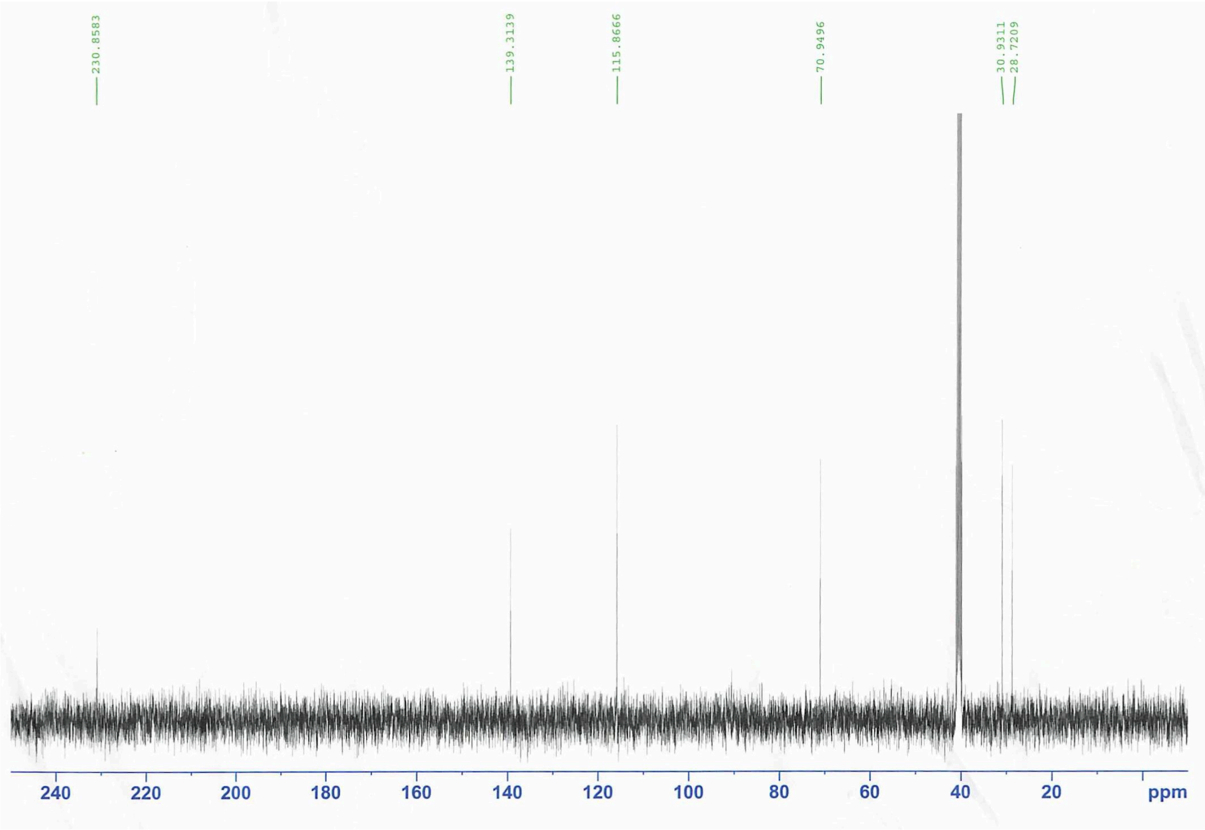

^{1}H NMR Spectra of Reported Compounds

^{1}H NMR (400 MHz) spectrum of *O*-ethyl *S*-phenyl carbonodithioate (**3b**) ($CDCl_3$)

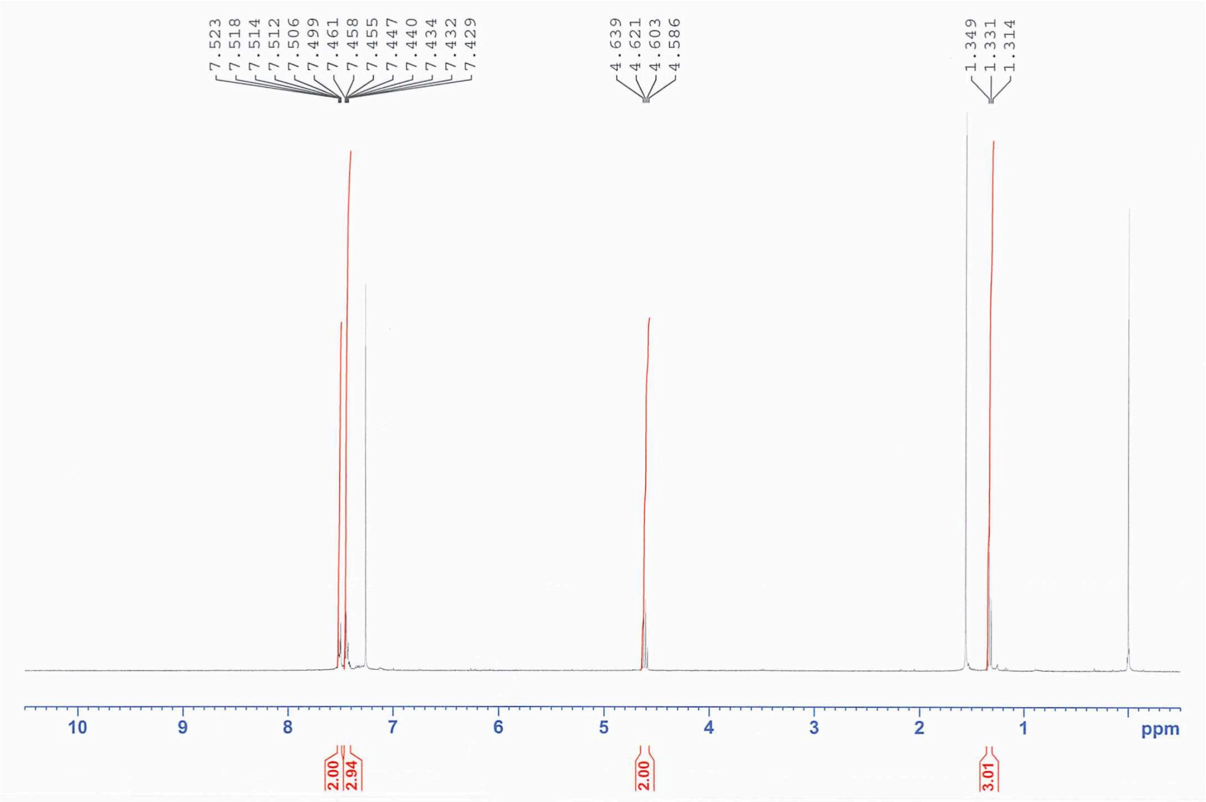

^{1}H NMR (400 MHz) spectrum of *O*-ethyl *S*-(3-methoxyphenyl) carbonodithioate (**3e**) ($CDCl_3$)

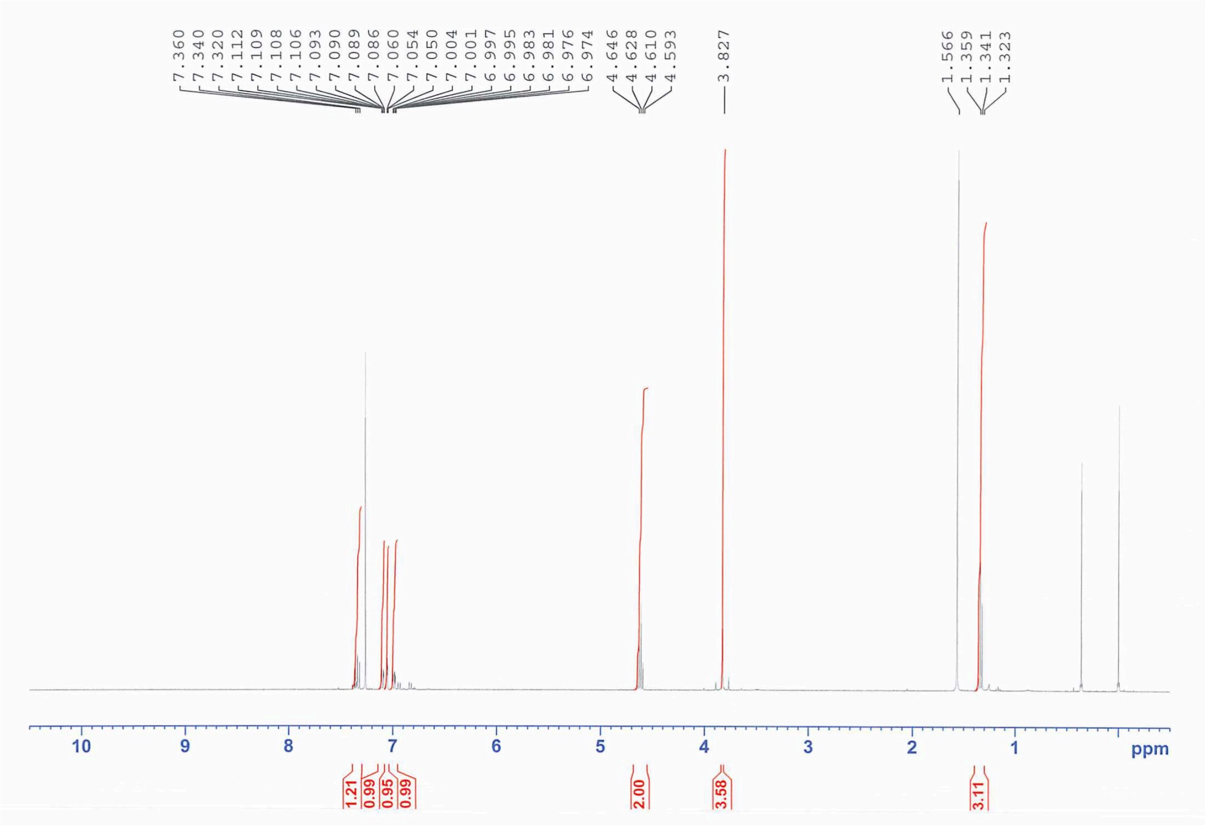

^{1}H NMR (400 MHz) spectrum of *S*-(2-bromophenyl) *O*-ethyl carbonodithioate (**8b**) ($CDCl_3$)

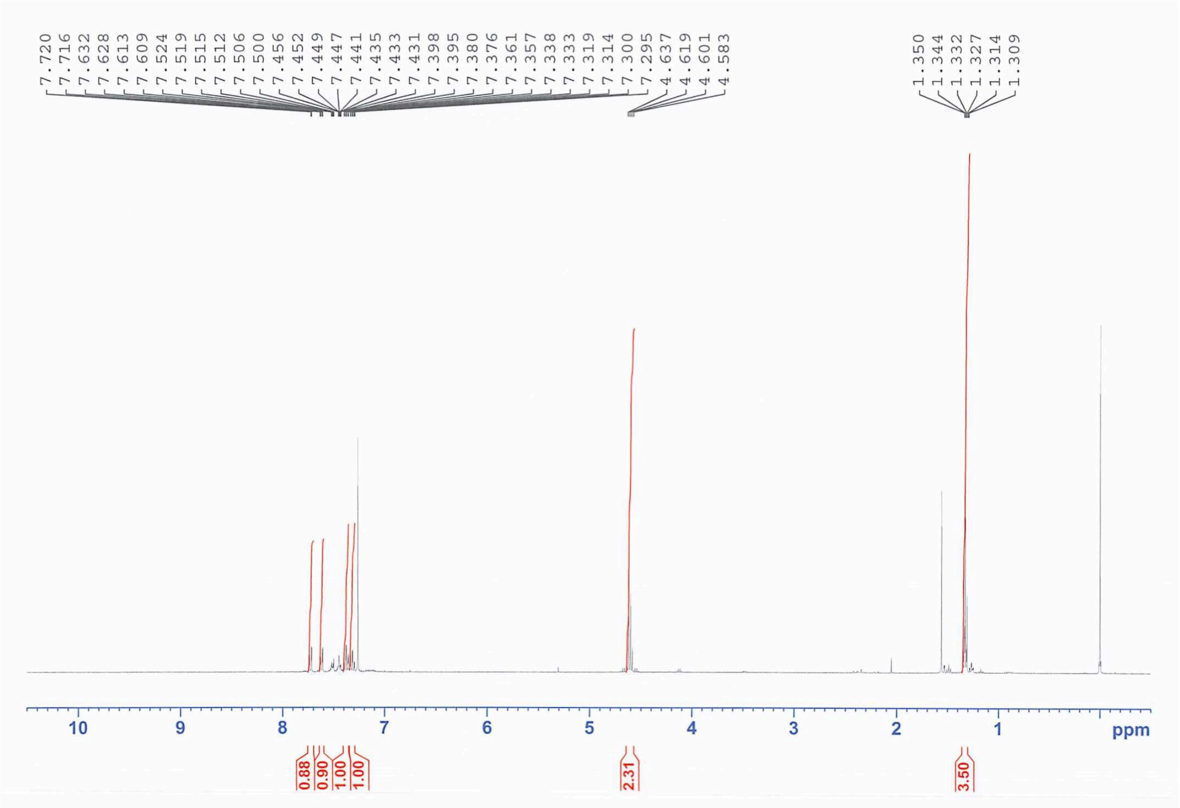

^{1}H NMR (400 MHz) spectrum of bis(3,4-dimethoxyphenyl) disulfide (**12**) ($CDCl_3$)

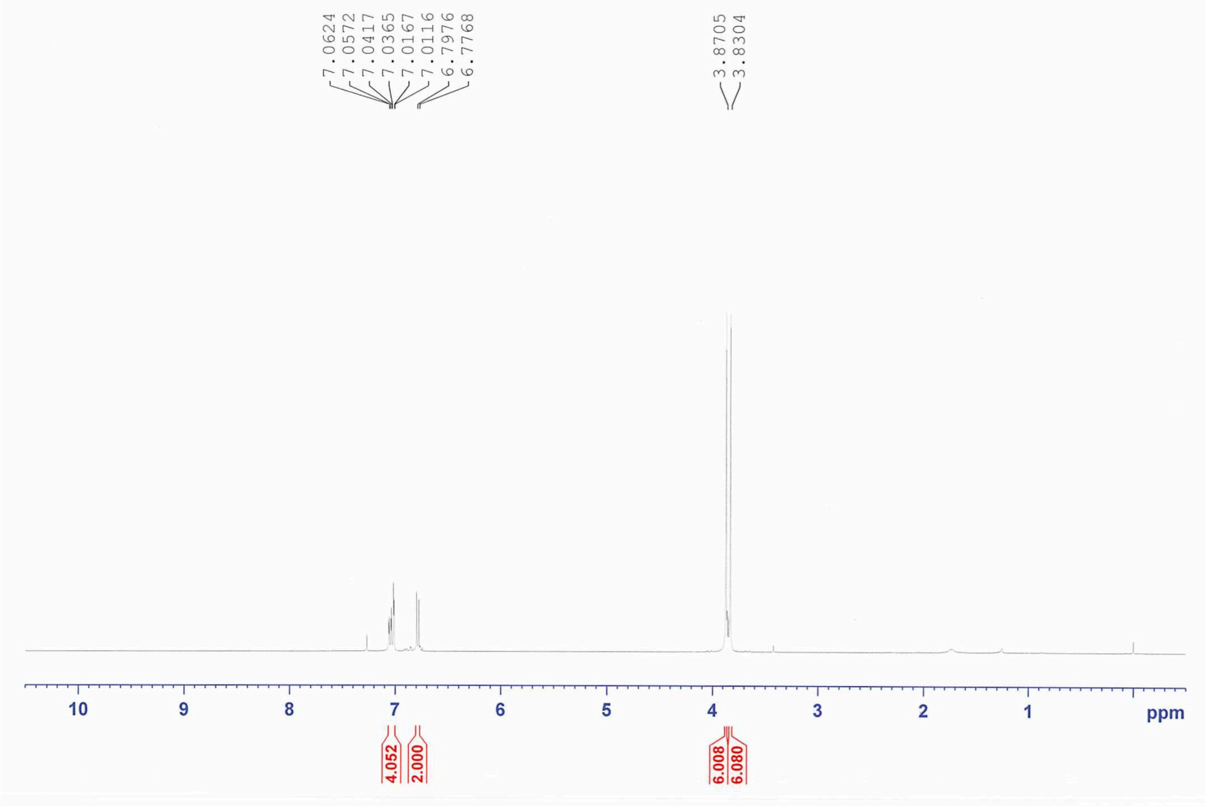

^{1}H NMR (400 MHz) spectrum of 3,4-dimethoxyphenyl methyl sulfide (**13a**) ($CDCl_3$)

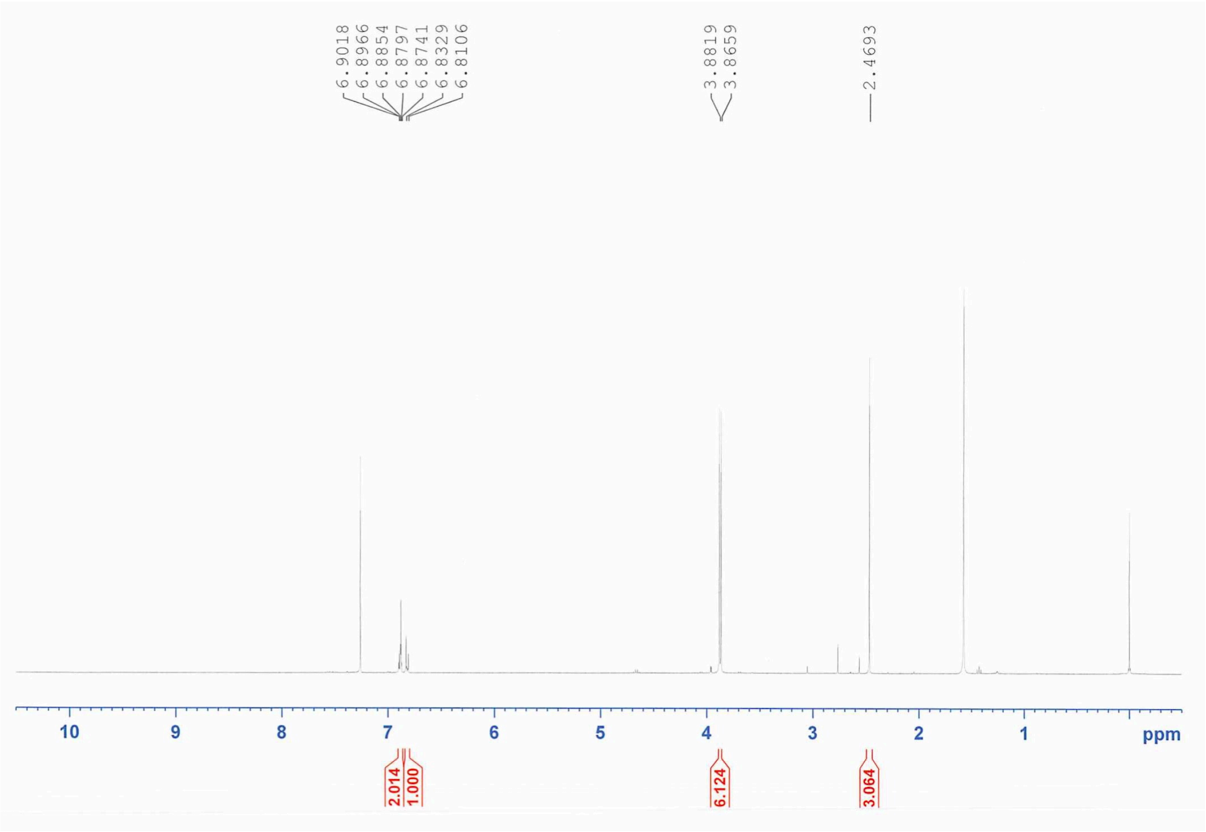

^{1}H NMR (400 MHz) spectrum of 3,4-dimethoxybenzenesulfonyl chloride (**14a**) ($CDCl_3$)

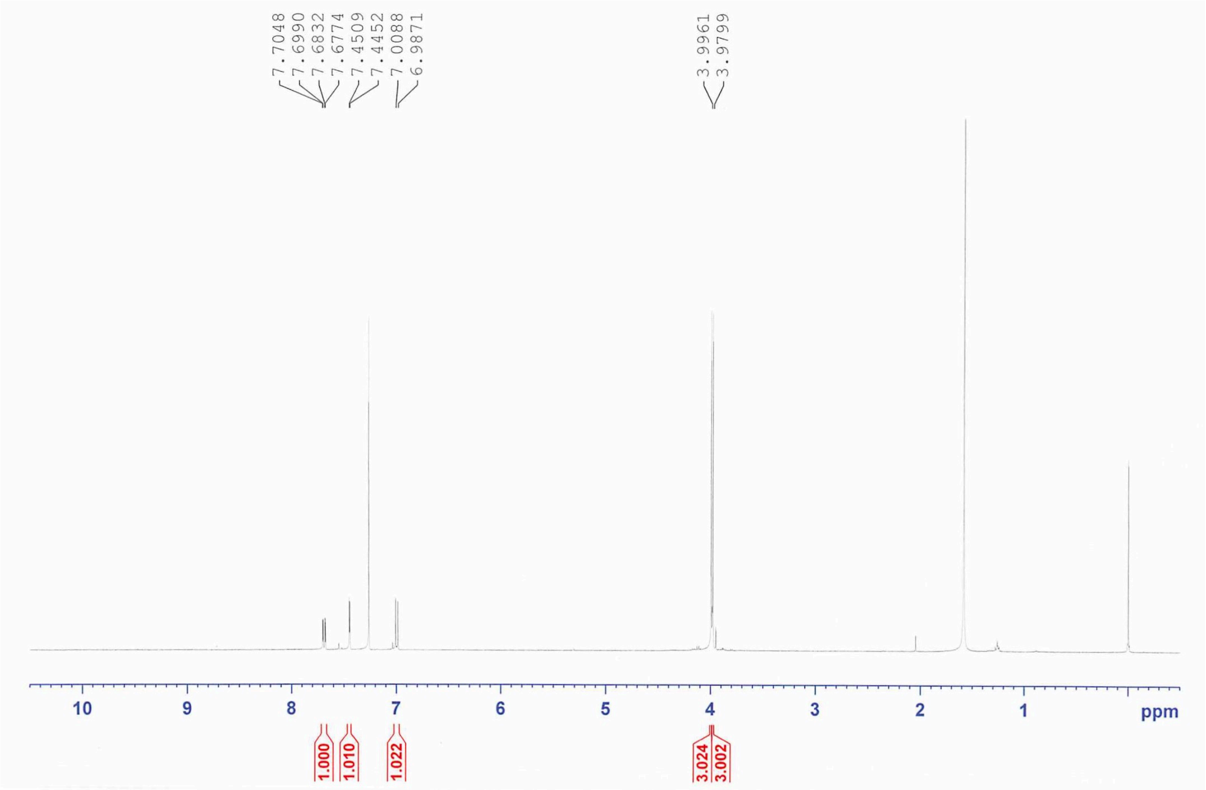

^{1}H NMR (400 MHz) spectrum of 3,4-dimethoxybenzenesulfonyl fluoride (**14b**) ($CDCl_3$)

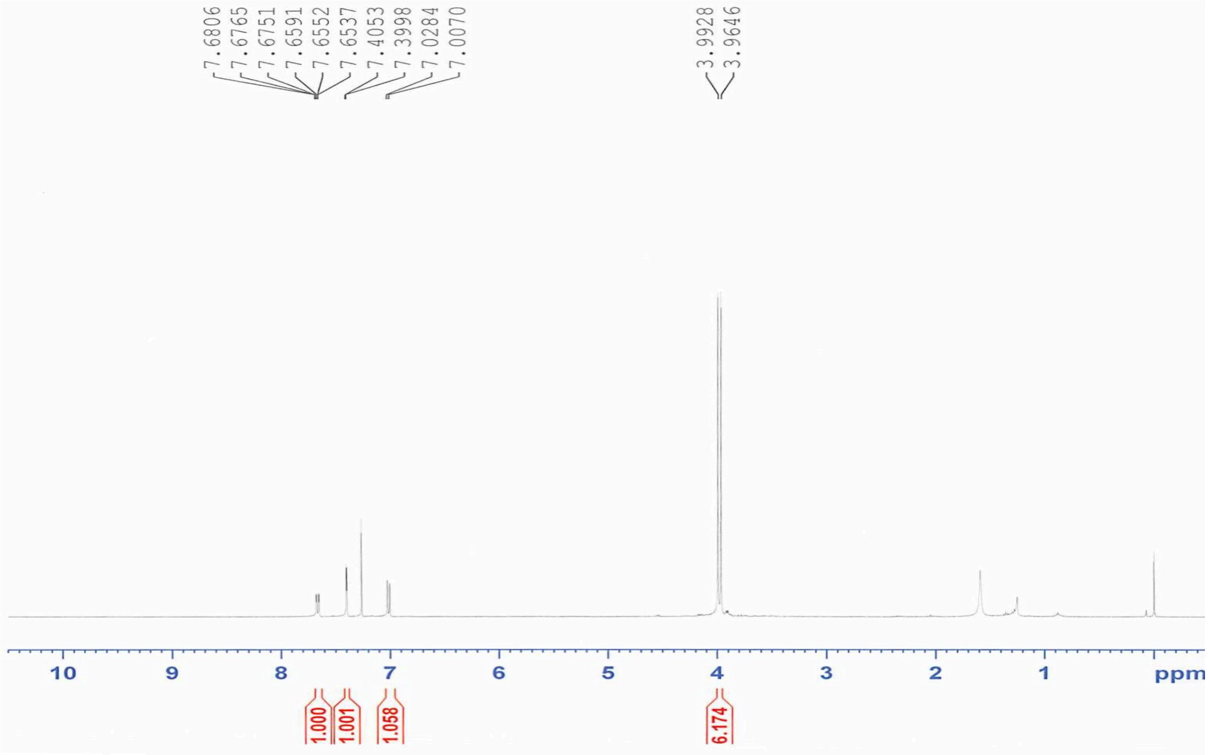

Supplement: Supplementary file 1 — ol4c00944_si_001.pdf [file ol4c00944_si_001.pdf]
